# Synthesis, Antitumor Evaluation and Molecular Docking of New Morpholine Based Heterocycles

**DOI:** 10.3390/molecules22071211

**Published:** 2017-07-20

**Authors:** Zeinab A. Muhammad, Mastoura M. Edrees, Rasha A. M. Faty, Sobhi M. Gomha, Seham S. Alterary, Yahia N. Mabkhot

**Affiliations:** 1 Department of Organic Chemistry, National Organization for Drug Control and Research (NODCAR), Giza 12311, Egypt; zeinab.a.muhammad@gmail.com (Z.A.M.); mmohamededrees@yahoo.com (M.M.E.); 2 Department of Chemistry, Faculty of Science, King Khalid University, Abha 61413, Saudi Arabia; 3 Department of Chemistry, Faculty of Science, Cairo University, Giza 12613, Egypt; rashafaty@yahoo.com; 4 Department of Chemistry, College of Science, King Saud University, P.O. Box 2455, Riyadh 11451, Saudi Arabia; salterary@ksu.edu.sa

**Keywords:** morpholine, chalcones, thiones, hydrazonoyl halides, anticancer activity, molecular docking

## Abstract

A series of new morpholinylchalcones was prepared and then used as building blocks for constructing a series of 7-morpholino-2-thioxo-2,3-dihydropyrido[2,3-*d*]pyrimidin-4(1*H*)-ones via their reaction with 6-aminothiouracil. The latter thiones reacted with the appropriate hydrazonoyl chloride to give the corresponding pyrido[2,3-*d*][1,2,4]triazolo[4,3-*a*]pyrimidin-5(1*H*)-ones. The assigned structures for all the newly synthesized compounds were confirmed on the basis of elemental analyses and spectral data and the mechanisms of their formation were also discussed. Most of the synthesized compounds were tested for in vitro activity against human lung cancer (A-549) and human hepatocellular carcinoma (HepG-2) cell lines compared with the employed standard antitumor drug (cisplatin) and the results revealed that compounds **8**, **4e** and **7b** have promising activities against the A-549 cell line (IC_50_ values of 2.78 ± 0.86 μg/mL, 5.37 ± 0.95 μg/mL and 5.70 ± 0.91 μg/mL, respectively) while compound **7b** has promising activity against the HepG-2 cell lines (IC_50_ = 3.54 ± 1.11 μg/mL). Moreover, computational studies using MOE 2014.09 software supported the biological activity results.

## 1. Introduction

In recent years, interest in the chemistry of hydrazonoyl halides has been renewed because of the development of novel synthetic routes and their use as synthons for compounds that find many applications [1,2,3,4,5,6,7,8,9,10]. The literature reveals that the presence of a morpholine ring on a heterocyclic system contributes to enhanced pharmacological activities in many cases [11,12,13,14,15,16]. In addition, many pyridopyrimidine derivatives have a variety of effects of chemical and biological significance such as antimicrobial, analgesic, anti-allergic, antitumor, antihypertensive, antileishmanial, antifolate, anti-inflammatory, ant-tuberculostatic, anticonvulsant, diuretic, potassium sparing, and anti-aggressive activities [17,18,19,20,21,22,23,24,25,26]. In view of all these reports and in continuation of our previous work on the synthesis of bioactive heterocyclic compounds [27,28,29,30,31,32,33], herein, we were interested in synthesizing a new series of morpholinylchalcones and utilizing them as precursors for the synthesis of pyridopyrimidines and triazolopyridopyrimidines to evaluate their anticancer activities. 

## 2. Results and Discussion

### 2.1. Chemistry

**Scheme 1:**
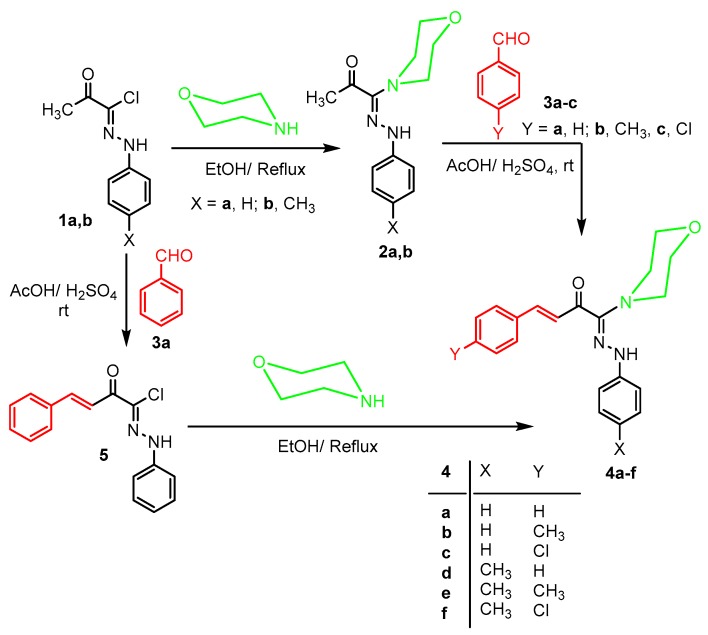
Synthesis of morpholinylchalcones **4a**–**f**.

The required starting compounds, namely 1-morpholino-1-(2-phenylhydrazono)propan-2-one (**2a**) and 1-morpholino-1-(2-(*p*-tolyl)hydrazono)propan-2-one (**2b**) were prepared by a previously reported method (Scheme 1) [34]. The morpholinohydrazonopropanone derivatives **2a**,**b** were next used as starting compounds for preparation of a number of novel chalcone derivatives. Thus stirring a mixture of 1-morpholino-1-(2-arylhydrazono)propan-2-ones **2a**,**b** and the appropriate benzaldehyde derivatives **3a**–**c** in glacial acetic acid in the presence of a catalytic amount of concentrated H_2_SO_4_, gave the morpholinylchalcone derivatives **4a**–**f** in good yield (Scheme 1). The assigned structures of the products **4a**–**f** was confirmed based on both elemental analyses and spectral data (IR, ^1^H-NMR and MS). The IR spectra of compounds **4a**–**f** revealed in each case two absorption bands in the regions υ 3250–3236, 1683–1676 cm^−1^ attributed to the NH and C=O groups. The ^1^H-NMR spectra of compounds **4a**–**f** showed in each case three signals assigned for the CH=CH and NH in addition to the expected signals for the morpholine and aromatic protons (see Experimental). For example, the ^1^H-NMR spectrum of compound **4a** taken as a typical example of the products **4**, revealed three signals at δ = 7.45 (d, *J* = 8 Hz, 1H, CH=CH), 7.79 (d, *J* = 8 Hz, 1H, CH=CH), 10.58 (brs, 1H, NH) and morpholine protons ppm in addition to the characteristic signals of the aromatic protons. The ^13^C-NMR spectrum of compound **4a** showed three signals at δ = 25.76, 68.04, 188.22 ppm assignable for the morpholine-C and the carbonyl-C, in addition to twelve aromatic and olifinic carbons. Moreover, the mass spectrum of **4a** revealed a molecular ion peak at *m*/*z* = 335 which is consistent with its expected molecular weight.

The structures assigned for products **4** were further evidenced via alternative method. Thus, condensation of 2-oxo-*N*-phenylpropanehydrazonoyl chloride (**1**) with benzaldehyde **3a** in glacial acetic acid in the presence of catalytic amount of concentrated H_2_SO_4_, afforded 2-oxo-*N*,4-diphenylbut-3-enehydrazonoyl chloride (**5**). Refluxing an equimolar amounts of **5** and morpholine in ethanol for 3 h, gave a product identical in all respects (m.p., mixed m.p. and IR spectra) with compound **4a** obtained from the **2a** + **3a** reaction (Scheme 1).

**Scheme 2:**
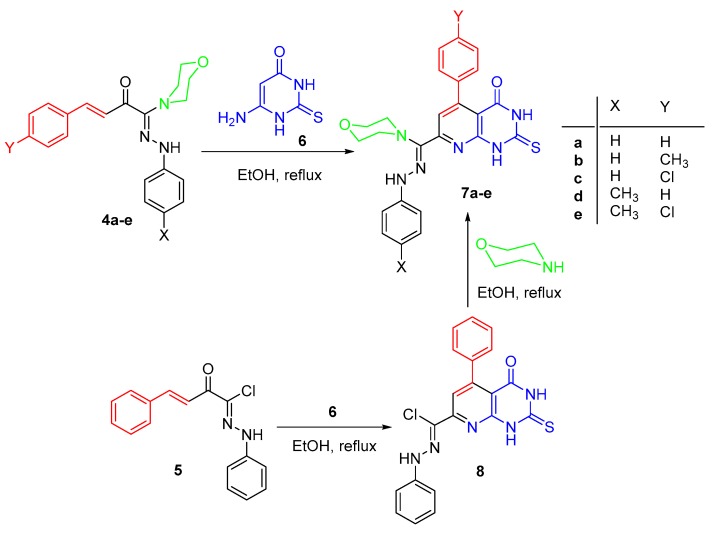
Synthesis of pyridopyrimidinethiones **7a**–**e**.

The morpholinylchalcone derivatives **4a**–**e** were then used for the preparation of novel series of 2-thioxo-2,3-dihydropyrido[2,3-*d*]pyrimidin-4(1*H*)-one derivatives bearing morpholine moieties. Thus, reaction of 6-amino-2-thioxo-2,3-dihydropyrimidin-4(1*H*)-one (**6**) and the appropriate chalcones **4a**–**e** in ethanol under reflux for 8–12 h led to the formation of one isolable product as evidenced by TLC analysis of the crude product (Scheme 2). The structures of the products was identified to be **7a**–**e** based on elemental analysis and spectral data. For example, the IR spectrum of **7a**–**e** revealed three absorption bands near υ_max_ 3125, 3243, 3447 cm^−1^ due to the three NH groups, and another absorption band near υ_max_ 1679 cm^−1^ attributed to the carbonyl group. The ^1^H-NMR spectra displayed three singlet signals near δ 9.67, 10.65 and 11.17 ppm attributed to the three NH protons (disappeared by d_2_o), in addition to the expected signals due to the morpholine and aryl protons. Also, the ^13^C-NMR spectra showed the expected number of aliphatic and aromatic signals. The mass spectra of the products **7a**–**e** revealed in each case a molecular ion peak *m*/*z* at the expected molecular weight calculated for each compound (see Experimental).

For a much more rigorous identification of the structures of compounds **7a**–**e**, a comparison with authentic material prepared from the reaction between thione **8** (obtained from reaction of chalcone **5** and compound **6**) with morpholine was achieved, the product obtained from this reaction was identical to that from the reaction of **4a** and **6** (Scheme 2).

**Scheme 3:**
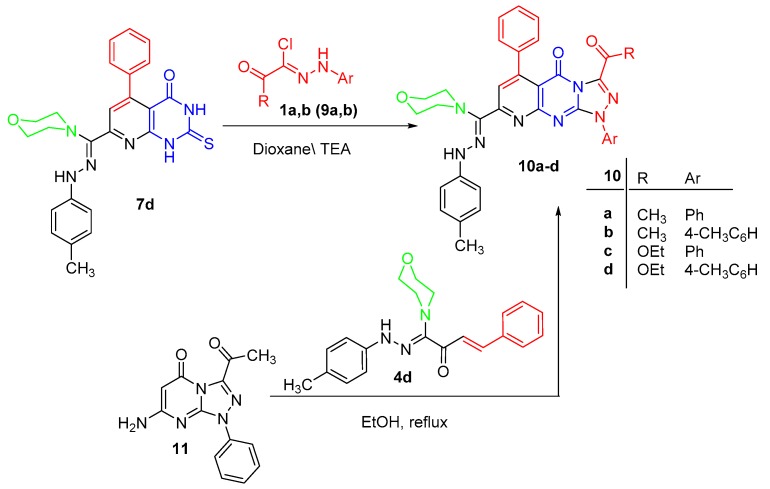
Synthesis of pyrido[2,3-*d*][1,2,4]triazolo[4,3-*a*]pyrimidin-5(1*H*)-ones **10a**–**d**.

On the other hand, compound **7d** was reacted with hydrazonoyl halides **1a**,**b** (**9a**,**b**) in dioxane in the presence of triethylamine to give 1,5-dihydropyrido[2,3-*d*][1,2,4]triazolo[4,3-*a*]pyrimidine derivatives **10a**–**d**, respectively (Scheme 3). Spectroscopic data as well as elemental analyses of the obtained products were in complete agreement with the assigned structures **10a**–**d** (see Experimental part).

An alternative synthetic route for the pyridotriazolopyrimidine derivatives **10a**–**d** was executed whereby compound **4d** was refluxed with 3-acetyl-7-amino-1-phenyl-[1,2,4]triazolo[4,3-*a*]pyrimidin-5(1*H*)-one (**11**) in ethanol afforded the respective authentic sample **10a** (Scheme 3).

### 2.2. Cytotoxic Activity

The in vitro growth inhibitory activity of the newly synthesized compounds **4a**,**c**,**e**,**f**, **5**, **7a**–**c**, **8** and **10a**–**d** was investigated against two carcinoma cell lines, a human lung cancer cell line (A-549) and a human hepatocellular carcinoma cell line (HepG-2), in comparison with a well-known anticancer standard drug (cisplatin) under the same conditions using a colorimetric MTT assay. Data generated were used to plot a dose response curve of which the concentration of test compounds required to kill 50% of cell population (IC_50_) was determined. The results are depicted in Table 1 revealed that the descending order of activity of the newly synthesized compounds towards the lung carcinoma cell line (A-549) were as follows: **8** > **4e** > **7b** > **7c** > **4f** > **7a** > **5** > **10d** > **10c** > **4a** > **4c** > **10a** > **10b**. The descending order of activity of the newly synthesized compounds towards the human hepatocellular carcinoma cell line (HepG-2) were as follows: **7b** > **7c** > **10d** > **4f** > **5** > **7a** > **10c** > **4e** > **10b** > **4c** > **4a** > **10a** > **8**.

#### Examination of the SAR Leads to the Following Conclusions

**Figure 1:**
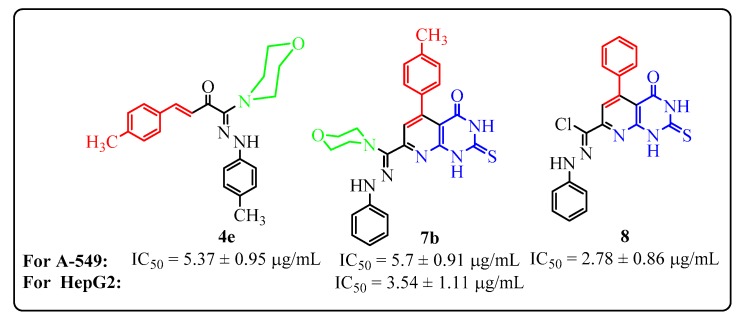
The most active compounds compared to cisplatin.

- The results revealed that all the tested compounds showed inhibitory activity to the tumor cell lines in a concentration dependent manner.
- The activities of the synthesized compounds depend on the structural skeleton and electronic environment of the molecules.
- Compounds **8**, **4e** and **7b** were the most active (IC_50_ values of 2.78 ± 0.86, 5.37 ± 0.95 and 5.70 ± 0.91 μg/mL, respectively) against the lung carcinoma cell line (A-549), compared with cisplatin reference drug with IC_50_ value of 0.95 ± 0.90 μg/mL (Figure 1), while the remaining compounds have moderate inhibitory activity (IC_50_ = 6.79 ± 1.11 − 26.8 ± 0.75 µg/mL).
- Compounds **7b**, **7c**, **10d** and **4f** were the most active (IC_50_ value of 3.54 ± 1.11, 8.42 ± 1.15, 8.72 ± 0.89 and 9.78 ± 0.78 μg/mL, respectively) against the human hepatocellular carcinoma cell line (HepG-2), compared with the reference drug cisplatin with an IC_50_ value of 1.40 ± 1.1μg/mL (Figure 1). The other compounds have moderate inhibitory activity (IC_50_ = 12.4 ± 0.98 − 29.9 ± 0.93 µg/mL).
- Among the morpholinylchalcone derivatives, the dimethylchalcone **4e** is the most active one against the A549 (IC_50_ = 5.37 ± 0.95 μg/mL) line, while the methylchlorochalcone **4f** is the most active one against the HepG-2 cell line (A549) (IC_50_ = 9.78 ± 0.78 μg/mL).
- For pyrido[2,3-*d*][1,2,4]triazolo[4,3-*a*]pyrimidin-5(1*H*)-ones **10a**–**d**: Compounds **10c** and **10d** (substituted with COOEt group at position 3) have more in vitro inhibitory activity than compounds **10a** and **10b** (substituted with a COCH_3_ group at position 3). Also compound **10d** is more active than **10c** where the *p*-substitution with a methyl group increases the activity via its +I effect.

### 2.3. Molecular Docking

A major problem in chemotherapy is resistance to the chemotherapeutic agents. There are generally two major forms of resistance encountered in the clinic. One is intrinsic resistance, which is a property of the tumor cells and is not triggered by drug exposure. The other is known as acquired resistance, which occurs following exposure to the drug(s). Anti-folates such as methotrexate (MTX) and fluoropyrimidines such as 5-fluorouracil (5-FU) have been used in the clinic for the management of childhood acute lymphoblastic leukemias (ALL) and colorectal cancer, respectively, with modest success. Among the several enzymes that participate in the synthesis of nucleic acid precursors, DHFR is an important target for several human dis-eases, namely, protozoal, bacterial and fungal infections, psoriasis, autoimmune diseases and neoplastic diseases. Traditionally, several DHFR inhibitors are reported as potential drug candidates in various diseases.

In the late 1950s DHFR was discovered as a ubiquitous enzyme with respect to drug design due to its central role in the synthesis of DNA. Most eukaryotic organisms synthesize the essential metabolite thymidylate via the thymidylate cycle, which consists of three enzymes: serine hydroxymethyl transferase; thymidylate synthase (TS) and the much promising DHFR. It reduces the NADPH-dependent 7,8-DHF to 5,6,7,8-THF utilizing NADPH^2+^ as cofactor. This THF acts in the conversion of deoxyuridylate (dUMP) to deoxythymidylate (dTMP) by thymidylate synthetase. Inhibition of TS or of DHFR leads to thymineless death, which has found clinical utility as antitmalarial, antiprotozoal and antimicrobial agents.

The MOE 2014.010 package software was used to analyze all docking poses and binding energies between compound **7b** and the enzyme dihydrofolate reductase (DHFR) to evaluate the affinity of **7b** according to its binding energy with the enzyme.

**Figure 2:**
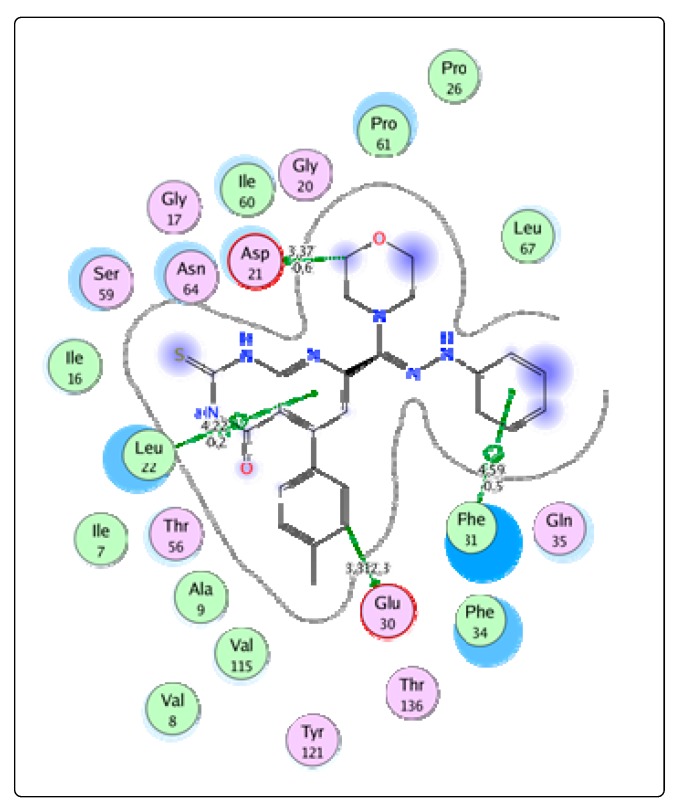
Hhydrogen bonding between the compound **7b** and the DHFR enzyme pocket amino acids.

**Figure 3:**
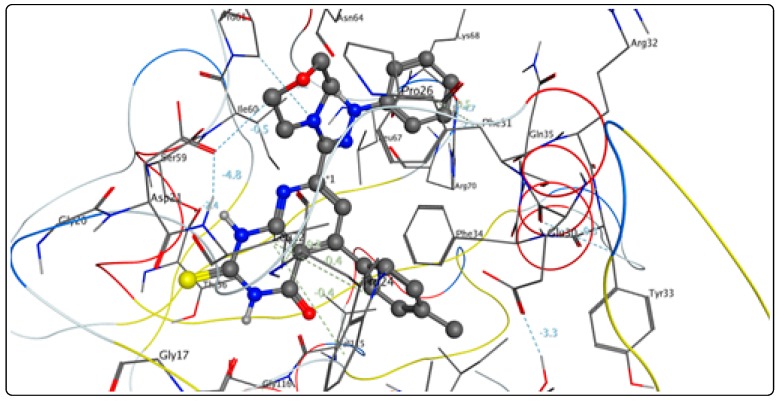
The interesting compound **7b** fitted into the enzyme pocket by interacting with amino acids found in the pocket.

From Figure 2 and Figure 3 which represent all the binding energies of the two compounds.it is clear that the total binding energy of compound **7b** equals −1.6 E (Kcal/mol), showing good affinity with the DHFR enzyme by forming four pi-hydrogen interactions with binding energy −1.4 E (kcal/mol) , one hydrogen acceptor interaction with binding energy −0.2 E (Kcal/mol) and one pi-pi interaction with almost zero binding energy.on the other hand compound comp showing affinity to the DHFR enzyme by −1.3 E (Kcal/mol) by making one hydrogen donor interaction with −0.6 E (Kcal/mol) and tow pi-hydrogen interactions with −0.7 E (Kcal/mol).

#### Bioactivity and ADME Toxicity

Due to its impact on society, the design of new drugs has the potential to interest a wide audience, and provides a rare opportunity to introduce several concepts in chemistry and biochemistry. Drug design can be seen as a multi-objective cyclic optimization process. Indeed, it is important to develop the understanding not only that a drug is generally an effective ligand for a protein of therapeutic interest, but also that these molecules need to have drug-like properties. Computer-aided drug design and bioinformatics approaches play a fundamental role in addressing these different challenges. Basically, drug design consists of the conception of molecules that are complementary to the protein target in terms of 3D-shape and charge distribution, to optimize molecular recognition and binding. On the contrary, ligand based approaches rely on the knowledge implicitly contained in the chemical structure or physical properties of other molecules that bind to the biological target of interest.

Molecular properties in relation to lipophilicity, drug likeness, or pharmacokinetics (PK), for example. These molecular properties are fundamental in drug design. Indeed, although a high affinity for the protein target is essential, it is not sufficient for the designed small molecule to become a drug: to obtain a therapeutic effect, the molecule needs to reach its target in the body, and stay there long enough for the expected biological events to occur. Therefore, to support efficiently the design of new drugs, it is important to predict their PK behaviors with computer-aided approaches.

A computational study was also carried out including prediction of pharmacokinetic properties, toxicity and bioactivity studies. In Table 2 Molecular properties were calculated on the basis of Lipinski’s rule and its components, Furthermore, TPSA values of the tested compounds, the prediction of bioactivity scores of the compounds were recorded by calculating the activity scores of GPCR, ion channel modulator, kinase inhibitor, nuclear receptor ligand, protease inhibitor and enzyme inhibitor.

Physicochemical properties, with an emphasis on lipophilicity computed by a variety of methodologies to enable a consensus approach by calculating the molecular weight and TPSA of the tested compounds. Druglikeness, estimated by simple rules to evaluate oral bioavailability through evaluating the compounds according to Lipinski rule five and also Pharmacokinetics, which predicts several ADME behaviors (e.g., substrate of P-glycoprotein, cytochromes P450, gastrointestinal absorption, brain blood barrier) by binary classification models relying on physicochemical descriptors. While medicinal chemistry that gives a score for synthetic accessibility, leadlikeness and pan-assay interference structure of molecules together with structural alerts for problematic fragments.

The docking study was performed using the MOE 2014.010 software. The crystal structure of the enzyme dihydrofolate reductase (DHFR, PDB ID (3NU0)) was downloaded out from Protein Data Bank website. Regularization and optimization for protein and ligand were performed. Determination of the essential amino acids in the binding site was carried out and compared with that presented in the literature. The performance of the docking method was evaluated by re-docking the crystal ligand into the assigned active dihydrofolate reductase (DHFR) enzyme to determine a RMSD value. Interactive docking to the selected active site was carried out for all the conformers of interesting compounds. Each docked compound was assigned a score according to its fit in the ligand binding pocket (LBP) and its binding mode.

## 3. Experimental

### 3.1. General Information 

Melting points were measured on an Electrothermal IA 9000 series digital melting point apparatus (Bibby Sci. Lim. Stone, Staffordshire, UK). IR spectra were measured on Shimadzu FTIR 8101 PC infrared spectrophotometers (Shimadzu, Tokyo, Japan) in potassium bromide discs. NMR spectra were measured on a Varian Mercury VX-300 NMR spectrometer (Varian, Inc., Karlsruhe, Germany) operating at 300 MHz (^1^H-NMR) and run in deuterated dimethylsulfoxide (DMSO-*d*_6_). Chemical shifts were related to that of the solvent. Mass spectra were recorded on a Shimadzu GCMS-QP1000 EX mass spectrometer at 70 eV. Elemental analyses were measured by using an Elementar Vario LIII CHNS analyzer (GmbH & Co.KG, Hanau, Germany). Antitumor activity of the products was measured at the Regional Center for Mycology and Biotechnology at Al-Azhar University, Cairo, Egypt. Hydrazonoyl halides were prepared following a literature method [35].

#### 3.1.1. Synthesis of Chalcones **4a**–**f**

A mixture of 1-morpholino-1-(2-arylhydrazono)propan-2-ones **2a**,**b** (10 mmol) and the appropriate benzaldehyde derivatives **3a**–**c** (10 mmol) in glacial acetic acid (20 mL) containing a few drops of concentrated H_2_SO_4_ was stirred at room temperature for 4 h (monitored by TLC). The formed precipitate after cooling was isolated by filtration, washed with ethanol, dried and recrystallized from ethanol to give products **4a**–**f**.

*1-Morpholino-4-phenyl-1-(2-phenylhydrazono)but-3-en-2-one* (**4a**), Yellow solid, (80% yield), m.p. 203–205 °C; IR (KBr) υ_max_ 1606 (C=N), 1678 (C=O), 2985, 2919 (C–H), 3243 (NH) cm^−1^; ^1^H-NMR (DMSO-*d*_6_) δ 2.30–2.34 (m, 4H, 2CH_2_), 3.65 (m, 4H, 2CH_2_), 6.57–7.33 (m, 10H, Ar–H), 7.45 (d, *J* = 8 Hz, 1H, CH=CH), 7.79 (d, *J* = 8 Hz, 1H, CH=CH), 10.58 (brs, 1H, NH); ^13^C-NMR (DMSO-*d*_6_): δ 25.76, 68.04 (CH_2_), 112.76, 114.82, 115.30, 122.76, 124.07, 129.49, 129.78, 129.89, 130.15, 132.25, 140.67 (Ar–C and C=N), 188.22 (C=O); MS *m*/*z* (%) 335 (M^+^, 5), 277 (20), 213 (24), 198 (62), 159 (47), 141 (38), 99 (43), 80 (42), 67 (48), 43 (100). Anal. Calcd. for C_20_H_21_N_3_O_2_ (335.40): C, 71.62; H, 6.31; N, 12.53. Found: C, 71.95; H, 6.11; N, 12.13%.

*1-Morpholino-1-(2-phenylhydrazono)-4-(p-tolyl)but-3-en-2-one* (**4b**), Yellow solid, (75% yield), m.p. 200–202 °C; IR (KBr) υ_max_ 1599 (C=N), 1680 (C=O), 3021, 2949 (C–H_2_), ^1^H-NMR (DMSO-*d*_6_) δ 7.05–7.60 (m, 9H, Ar-H), 7.87 (d, *J* = 8 Hz, 1H, CH=CH), 8.37 (d, *J* = 8 Hz, 1H, CH=CH), 10.59 (brs, 1H, NH); MS *m*/*z* (%) 349 (M^+^, 6), 340 (12), 268 (28), 236 (11), 164 (6), 123 (7), 108 (19), 92 (72), 76 (55), 68 (22), 65 (65), 43 (100). Anal. Calcd. for C_21_H_23_N_3_O_2_ (349.43): C, 72.18; H, 6.63; N, 12.03. Found: C, 72.51; H, 6.40; N, 11.80%.

*4-(4-Chlorophenyl)-1-morpholino-1-(2-phenylhydrazono)but-3-en-2-one* (**4c**), Yellow solid, (85% yield), m.p. 192–194 °C; IR (KBr) υ_max_ 1599 (C=N), 1680 (C=O), 3019, 2950 (C–H), 3245 (NH) cm^−1^; ^1^H-NMR (DMSO-*d*_6_) δ 2.20–2.35 (m, 4H, 2CH_2_), 3.54 (m, 4H, 2CH_2_), 7.09–7.59 (m, 9H, Ar–H), 7.84 (d, *J* = 8 Hz, 1H, CH=CH), 8.37 (d, *J* = 8 Hz, 1H, CH=CH), 10.75 (brs, 1H, NH); MS *m*/*z* (%) 371 (M^+^ + 2, 1), 369 (M^+^, 4), 196 (3), 165 (4), 123 (5), 111 (8), 97 (11), 83 (20), 77 (20), 69 (42), 57 (47), 43 (100), 41 (25). Anal. Calcd. for C_20_H_20_N_3_O_2_Cl (369.84): C, 64.95; H, 5.45; N, 11.36. Found: C, 63.78; H, 5.05; N, 11.02%.

*1-Morpholino-4-phenyl-1-(2-(p-tolyl)hydrazono)but-3-en-2-one* (**4d**), Yellow solid, (70% yield), m.p. 188–190 °C; IR (KBr) υ_max_ 1599 (C=N), 1683 (C=O), 3021, 2948 (C-H), 3236 (NH) cm^−1^; ^1^H-NMR (DMSO-*d*_6_) δ 2.18–2.47 (m, 4H, 2CH_2_), 2.53 (s, 3H, CH_3_), 3.54 (m, 4H, 2CH_2_), 7.06–7.60 (m, 9H, Ar–H), 7.86 (d, *J* = 8 Hz, 1H, CH=CH), 8.38 (d, *J* = 8 Hz, 1H, CH=CH), 10.45 (brs, 1H, NH); ^13^C-NMR (DMSO-*d*_6_): δ 21.83 (CH_3_), 26.76, 69.38, (CH_2_), 111.76, 114.82, 115.30, 123.76, 124.07, 129.39, 129.78, 130.89, 131.15, 132.25, 140.67 (Ar–C and C=N), 188.22 (C=O); MS *m*/*z* (%) 349 (M^+^, 6), 340 (12), 268 (28), 236 (11), 164 (6), 123 (7), 108 (19), 92 (72), 76 (55), 68 (22), 65 (65), 43 (100). Anal. Calcd. for C_21_H_23_N_3_O_2_ (349.43): C, 72.18; H, 6.63; N, 12.03. Found: C, 72.55; H, 5.05; N, 12.44%.

*1-Morpholino-4-(p-tolyl)-1-(2-(p-tolyl)hydrazono)but-3-en-2-one* (**4e**), Yellow solid, (70% yield), m.p. 203–205 °C; IR (KBr) υ_max_ 1608 (C=N), 1678 (C=O), 2985, 2918 (C–H), 3250 (NH) cm^−1^; ^1^H-NMR (DMSO-*d*_6_) δ 2.13–2.41 (m, 4H, 2CH_2_), 2.45 (s, 3H, CH_3_), 2.58 (s, 3H, CH_3_), 3.54 (m, 4H, 2CH_2_), 6.91–7.72 (m, 8H, Ar–H), 7.82 (d, *J* = 8 Hz, 1H, CH=CH), 8.40 (d, *J* = 8 Hz, 1H, CH=CH), 10.80 (brs, 1H, NH); MS *m*/*z* (%) 363 (M^+^, 2), 321 (2), 291 (3), 275 (4), 233 (4), 208 (5), 178 (3), 119 (40), 106 (45), 91 (100), 77 (37), 65 (49), 43 (75). Anal. Calcd. for C_22_H_25_N_3_O_2_ (363.45): C, 72.70; H, 6.93; N, 11.56. Found: C, 73.02; H, 6.55; N, 11.16%.

*4-(4-Chlorophenyl)-1-morpholino-1-(2-(p-tolyl)hydrazono)but-3-en-2-one* (**4f**), Yellow solid, (80% yield), m.p. 206–208 °C; IR (KBr) υ_max_ 1613 (C=N), 1676 (C=O), 2974, 2940 (C–H), 3243 (NH) cm^−1^; ^1^H-NMR (DMSO-*d*_6_) δ 2.24 (s, 3H, CH_3_), 2.42–2.47 (m, 4H, 2CH_2_), 3.74 (m, 4H, 2CH_2_), 7.12–7.53 (m, 8H, Ar–H), 7.79 (d, *J* = 8 Hz, 1H, CH=CH), 7.91 (d, *J* = 8 Hz, 1H, CH=CH), 10.58 (brs, 1H, NH); MS *m*/*z* (%) 385 (M^+^ + 2, 1), 383 (M^+^, 3), 313 (3), 262 (3), 210 (4), 165 (4), 139 (24), 121 (10), 106 (27), 91 (36), 77 (42), 69 (58), 57 (62), 43 (100). Anal. Calcd. for C_21_H_22_N_3_O_2_Cl (383.87): C, 65.71; H, 5.78; N, 6.94. Found: C, 65.95; H, 5.55; N, 6.53%.

#### 3.1.2. Synthesis of 2-Oxo-*N*,4-diphenylbut-3-enehydrazonoyl Chloride (**5**)

A mixture of 2-oxo-*N*-phenylpropanehydrazonoyl chloride **1a** (0.98 g, 5 m mol) and the benzaldehyde **3a** (0.53 g, 5 mmol) in glacial acetic acid (20 mL) containing drops of concentrated H_2_SO_4_ was stirred at room temperature for 4 h (monitored by TLC). The formed precipitate after cooling was isolated by filtration, washed with ethanol, dried and recrystallized from ethanol to give product **5** as yellow solid, (70% yield), m.p. 209–211 °C; IR (KBr) υ_max_ 1601 (C=N), 1680 (C=O), 2950, 3018, 3055, (C–H), 3245 (NH) cm^−1^; ^1^H-NMR (DMSO-*d*_6_) δ 6.99 (d, *J* = 6 Hz, 1H, CH=CH–CO), 7.01–7.44 (m, 10H, Ar–H), 7.33 (d, *J* = 6 Hz, 1H, CH=CH–CO), 10.64 (brs, 1H, NH); MS *m*/*z* (%) 286 (M^+^ + 2, 2), 284 (M^+^, 7), 262 (9), 239 (15), 137 (12), 123 (11), 109 (31), 97 (22), 81 (50), 69 (64), 57 (63), 43 (100). Anal. Calcd. for C_16_H_13_ClN_2_O (284.74): C, 67.49; H, 4.60; N, 9.84. Found: C, 67.82; H, 4.20; N, 9.55%.

#### 3.1.3. Alternative Synthesis of **4a**

Equimolar amounts of **5** (0.284 g, l mmol) and morpholine (0.87 g, 1 mmol) in ethanol (15 mL) was refluxed for 3 h, gave product identical in all respects (m.p., mixed m.p. and IR spectra) with compound **4a** which obtained from reaction of **2a** + **3a**.

#### 3.1.4. Synthesis of Thiones **7a**–**e**

A mixture of 6-amino-2-thioxo-2,3-dihydropyrimidin-4(1*H*)-one (**6**) (0.143 g, 1 mmol) and the appropriate chalcones **4a**–**e** (1 mmol) in ethanol (20 mL) was refluxed for 8–12 h (monitored by TLC). The formed precipitate after cooling was isolated by filtration, washed with ethanol, dried and recrystallized from ethanol to give products **7a**–**e**.

*7-(Morpholino(2-phenylhydrazono)methyl)-5-phenyl-2-thioxo-2,3-dihydropyrido[2,3-d]pyrimidin-4(1H)-one* (**7a**), Yellow solid, (70% yield), m.p. 150–152 °C; IR (KBr) υ_max_ 1613 (C=N), 1679 (C=O), 2919, 2983, 3030 (C–H), 3125, 3243, 3447 (3NH) cm^−1^; ^1^H-NMR (DMSO-*d*_6_) δ 2.30–2.42 (m, 4H, 2CH_2_), 3.01–308 (m, 4H, 2CH_2_), 7.01–7.94 (m, 11H, Ar–H), 9.67, 10.65, 11.17 (3brs, 3H, 3NH); ^13^C-NMR (DMSO-*d*_6_): δ 25.80, 78.62 (CH_2_), 112.96, 115.30, 119.41, 123.23, 123.27, 125.07, 128.83, 129.74, 131.13, 132.11, 132.96, 138.02, 142.96, 148.36, 154.78 (Ar–C and C=N), 162.08 (C=O), 175.01 (C=S); MS *m*/*z* (%) 458 (M^+^, 3), 313 (5), 236 (6), 192 (13), 152 (4), 129 (9), 121 (10), 107 (13), 98 (20), 97 (33), 81 (37), 71 (50), 57 (79), 43 (100). Anal. Calcd. for C_24_H_22_N_6_O_2_S (458.54): C, 62.87; H, 4.84; N, 18.33. Found: C, 63.17; H, 4.71; N, 18.16%.

*7-(Morpholino(2-phenylhydrazono)methyl)-2-thioxo-5-(p-tolyl)-2,3-dihydropyrido[2,3-d]pyrimidin-4(1H)-one* (**7b**), Yellow solid, (70% yield), m.p. 158–160 °C; IR (KBr) υ_max_ 1608 (C=N), 1679 (C=O), 2926, 3057 (C–H), 3175, 3247, 3425 (3NH) cm^−1^; ^1^H-NMR (DMSO-*d*_6_) δ 2.24 (s, 3H, CH_3_), 2.30 (m, 4H, 2CH_2_), 3.31 (m, 4H, 2CH_2_), 7.12–7.33 (m, 10H, Ar–H), 10.57, 11.48, 11.58 (3brs, 3H, 3NH); MS *m*/*z* (%) 472 (M^+^, 1), 368 (9), 313 (7), 237 (7), 178 (6), 143 (54), 115 (16), 98 (22), 83 (24), 69 (44), 43 (100). Anal. Calcd. for C_25_H_24_N_6_O_2_S (472.57): C, 63.54; H, 5.12; N, 17.78. Found: C, 63.68; H, 4.05; N, 17.59%.

*5-(4-Chlorophenyl)-7-(morpholino(2-phenylhydrazono)methyl)-2-thioxo-2,3-dihydropyrido [2,3-d]pyrimidin-4(1H)-one* (**7c**), Yellow solid, (70% yield), m.p. 149–151 °C; IR (KBr) υ_max_ 1608 (C=N), 1695 (C=O), 2954, 3038 (C–H), 3172, 3397, 3412 (3NH) cm^−1^; ^1^H-NMR (DMSO-*d*_6_) δ 2.36 (m, 4H, 2CH_2_), 3.38 (m, 4H, 2CH_2_), 6.99–7.44 (m, 10H, Ar–H), 10.64, 11.49, 11.59 (3brs, 3H, 3NH); MS *m*/*z* (%) 494 (M^+^, 12), 492 (39), 390 (38), 202 (39), 161 (23), 127 (100), 84 (80), 77 (82). Anal. Calcd. for C_24_H_21_ClN_6_O_2_ (492.98): C, 58.47; H, 4.29; N, 17.05. Found: C, 58.78; H, 4.01; N, 16.70%.

*7-(Morpholino(2-(p-tolyl)hydrazono)methyl)-5-phenyl-2-thioxo-2,3-dihydropyrido[2,3-d]pyrimidin-4(1H)-one* (**7d**), Yellow solid, (70% yield), m.p. 183–185 °C; IR (KBr) υ_max_ 1600 (C=N), 1637 (C=O), 2950, 3058 (C–H), 3246, 3330, 3426 (3NH) cm^−1^; ^1^H-NMR (DMSO-*d*_6_) δ 2.23 (m, 4H, 2CH_2_), 2.47 (s, 3H, CH_3_), 3.35 (m, 4H, 2CH_2_), 6.95–7.42 (m, 10H, Ar–H), 10.64, 11.49, 11.59 (3brs, 3H, 3NH); MS *m*/*z* (%) 472 (M^+^, 10), 368 (9), 313 (7), 237 (7), 178 (6), 143 (54), 115 (16), 98 (22), 83 (24), 69 (44), 55 (45), 43 (100). Anal. Calcd. for C_25_H_24_N_6_O_2_S (472.56): C, 63.54; H, 5.12; N, 17.78. Found: C, 63.78; H, 4.05; N, 17.19%.

*5-(4-Chlorophenyl)-7-(morpholino(2-(p-tolyl)hydrazono)methyl)-2-thioxo-2,3-dihydropyrido[2,3-d]-pyrimidin-4(1H)-one* (**7e**), Yellow solid, (70% yield), m.p. 160–162 °C; IR (KBr) υ_max_ 1601 (C=N), 1676 (C=O), 2972, 3031 (C–H), 3243, 3326, 3425 (3NH) cm^−1^; ^1^H-NMR (DMSO-*d*_6_) δ 2.24 (s, 3H, CH_3_), 2.46 (m, 4H, 2CH_2_), 3.32 (m, 4H, 2CH_2_), 7.12–7.33 (m, 9H, Ar–H), 10.57, 11.49, 11.59 (3brs, 3H, 3NH); ^13^C-NMR (DMSO-*d*_6_): δ 20.80 (CH_3_), 25.76, 78.62 (CH_2_), 111.93, 115.30, 122.76, 125.37, 130.15, 132.25, 135.47, 140.67, 142.01, 145.96, 147.13, 148.22, 152.84, 154.77 (Ar–C and C=N), 162.07 (C=O), 175.02 (C=S); MS *m*/*z* (%) 509 (M^+^ + 2, 5), 507 (M^+^, 14), 409 (11), 304 (38), 272 (24), 240 (42), 208 (20), 195 (17), 174 (39), 163 (23), 131 (28), 79 (100). Anal. Calcd. for C_25_H_23_ClN_6_O_2_S (507.01): C, 59.22; H, 4.57; N, 16.58. Found: C, 59.25; H, 4.05; N, 16.21%.

#### 3.1.5. Alternative Synthesis of **7a**

Equimolar amounts of **8** (0.407 g, l mmol) and morpholine (0.87 g, 1 mmol) in ethanol (15 mL) was refluxed for 3 h, gave product identical in all respects (m.p., mixed m.p. and IR spectra) with compound **7a** which obtained from reaction of **4a** + **6**.

*Synthesis of 4-oxo-N,5-diphenyl-2-thioxo-1,2,3,4-tetrahydropyrido[2,3-d]pyrimidine-7-carbohydrazonoyl chloride* (**8**), A mixture of **5** (0.568 g, 2 mmol) and 6-amino-2-thioxo-2,3-dihydropyrimidin-4(1*H*)-one (**6**) (0.286 g, 2 mmol) in ethanol (20 mL) was refluxed for 6 h (monitored by TLC). The formed precipitate after cooling was isolated by filtration, washed with ethanol, dried and recrystallized from ethanol to give product **8** as yellow solid, Yellow solid, (70% yield), m.p. 203–205 °C; IR (KBr) υ_max_ 1588 (C=N), 1641 (C=O), 2973, 3097, 3199 (C–H), 3242, 3316, 3424 (3NH) cm^−1^; ^1^H-NMR (DMSO-*d*_6_) δ 6.35–7.43 (m, 11H, Ar–H), 10.64, 11.49, 11.59 (3brs, 3H, 3NH); MS *m*/*z* (%) 409 (M^+^ + 2, 4), 407 (M^+^, 10), 392 (25), 370 (10), 344 (26), 332 (15), 310 (100), 283 (33), 268 (74), 108 (10), 92 (39), 77 (24), 56 (16). Anal. Calcd. for C_20_H_14_ClN_5_OS (407.88): C, 58.90; H, 3.46; N, 17.17. Found: C, 59.24; H, 3.15; N, 16.89%.

#### 3.1.6. Synthesis of Pyrido[2,3-*d*][1,2,4]triazolo[4,3-*a*]pyrimidin-5(1*H*)-ones **10a**–**d**

To a mixture of equimolar amounts of thione **7d** (0.472 g, 1 mmol) and the appropriate hydrazonoyl halides **1a**,**b** (**9a**,**b**) (1 mmol of each) in dioxane (20 mL) was added triethylamine (0.1 mL, 1 mmol). The reaction mixture was refluxed till all of the starting materials have disappeared and hydrogen sulfide gas ceased to evolve (6–12 h monitored by TLC). The solvent was evaporated and the residue was treated with methanol. The solid that formed was filtered off and crystallized from the appropriate solvent to give compounds **10a**–**d**.

*3-Acetyl-8-(morpholino(2-(p-tolyl)hydrazono)methyl)-1,6-diphenylpyrido[2,3-d][1,2,4]triazolo[4,3-a]-pyrimidin-5(1H)-one* (**10a**), Browen solid, (70% yield), m.p. 230–232 °C; IR (KBr) υ_max_ 1594 (C=N), 1654, 1675 (2C=O), 2933, 3042 (C–H), 3429 (NH) cm^−1^; ^1^H-NMR (DMSO-*d*_6_) δ 2.24 (s, 3H, CH_3_), 2.42 (m, 4H, 2CH_2_), 2.61 (s, 3H, CH_3_), 3.54 (m, 4H, 2CH_2_), 6.59–7.93 (m, 15H, Ar–H), 11.22 (brs, 1H, NH); ^13^C-NMR (DMSO-*d*_6_): δ 20.51, 25.58 (CH_3_), 26.03, 66.80 (CH_2_), 113.17, 114.84, 115.36, 120.58, 122.46, 124.03, 125.64, 125.77, 129.43, 129.61, 129.81, 129.91, 130.01, 130.30, 130.33, 130.46, 130.78, 132.16, 134.83, 140.43 (Ar–C and C=N), 168.19, 196.22 (2C=O); MS *m*/*z* (%) 598 (M^+^, 3), 353 (12), 247 (20), 196 (15), 121 (15), 108 (27), 105 (24), 92 (49), 77 (47), 69 (21), 65 (25), 43 (100). Anal. Calcd. for C_34_H_30_N_8_O_3_ (598): C, 68.21; H, 5.05; N, 18.72. Found: C, 68.55; H, 4.85; N, 18.41%.

*3-Acetyl-8-(morpholino(2-(p-tolyl)hydrazono)methyl)-6-phenyl-1-(p-tolyl)pyrido[2,3-d][1,2,4]triazolo[4,3-a]pyrimidin-5(1H)-one* (**10b**), Brown solid, (70% yield), m.p. 238–240 °C; IR (KBr) υ_max_ 1596 (C=N), 1653, 1682 (2C=O), 2970, 3027 (C–H), 3434 (NH) cm^−1^; ^1^H-NMR (DMSO-*d*_6_) δ 2.24 (s, 3H, CH_3_), 2.37 (m, 4H, 2CH_2_), 2.40 (s, 3H, CH_3_), 2.63 (s, 3H, CH_3_), 3.41 (m, 4H, 2CH_2_), 7.03–7.76 (m, 14H, Ar–H), 11.17 (brs, 1H, NH); MS *m*/*z* (%) 612 (M^+^, 4), 519 (4), 494 (7), 460 (13), 443 (63), 429 (34), 400 (30), 384 (32), 337 (12), 315 (18), 291 (16), 216 (30), 201 (62), 187 (53), 126 (43), 68 (100). Anal. Calcd. for C_35_H_32_N_8_O_3_ (612.68): C, 68.61; H, 5.26; N, 18.29. Found: C, 68.93; H, 5.01; N, 17.89%.

*Ethyl 8-(morpholino(2-(p-tolyl)hydrazono)methyl)-5-oxo-1,6-diphenyl-1,5-dihydropyrido[2,3-d][1,2,4]triazolo[4,3-a]pyrimidine-3-carboxylate* (**10c**), Brown solid, (70% yield), m.p. 244–245 °C; IR (KBr) υ_max_ 1597 (C=N), 1658, 1712 (2C=O), 2979, 3061 (C–H), 3427 (NH) cm^−1^; ^1^H-NMR (DMSO-d*6*) δ 1.26 (t, *J* = 7.2 Hz, 3H, CH_3_CH_2_), 2.31–2.43 (m, 4H, 2CH_2_), 2.59 (s, 3H, CH_3_), 3.54–360 (m, 4H, 2CH_2_), 4.24 (q *J* = 7.2 Hz, 2H, CH_3_CH_2_), 6.97–7.97 (m, 15H, Ar–H), 10.52 (brs, 1H, NH); ^13^C-NMR (DMSO-*d*_6_): δ 14.56, 25.61 (2CH_3_)*,* 27.43, 62.62, 69.48 (3CH_2_), 115.01, 115.05, 115.36, 120.40, 122.65, 122.87, 126.44, 129.11, 129.37, 129.41, 129.68, 129.75, 129.78, 129.85, 129.94, 130.01, 130.36, 130.41, 138.74, 143.12 (Ar–C and C=N), 162.52, 174.52 (2C=O); MS *m*/*z* (%) 628 (M^+^, 7), 511 (18), 472 (9), 397 (6), 209 (14), 161 (23), 127 (10), 119 (26), 104 (88), 84 (100), 77 (69). Anal. Calcd. for C_35_H_32_N_8_O_4_ (628.68): C, 66.87; H, 5.13; N, 17.82. Found: C, 67.21; H, 4.80; N, 17.55%.

*Ethyl 8-(morpholino(2-(p-tolyl)hydrazono)methyl)-5-oxo-6-phenyl-1-(p-tolyl)-1,5-dihydropyrido [2,3-d][1,2,4]triazolo[4,3-a]pyrimidine-3-carboxylate* (**10d**), Brown solid, (70% yield), m.p. 256–258 °C; IR (KBr) υ_max_ 1591 (C=N)1663,1717 (2C=O), 2979, 3028 (C–H), 3436 (NH) cm^−1^; ^1^H-NMR (DMSO-*d*_6_) δ 1.21 (t, *J* = 7.2 Hz, 3H, CH_3_CH_2_), 2.24 (s, 3H, CH_3_), 2.37 (s, 3H, CH_3_), 2.48 (m, 4H, 2CH_2_), 4.25 (q, *J* = 7.2 Hz, 2H, CH_3_CH_2_), 3.40 (m, 4H, 2CH_2_), 6.95–7.98 (m, 14H, Ar–H), 11.18 (brs, 1H, NH); MS *m*/*z* (%) 642 (M^+^, 12), 602 (21), 511 (18), 472 (9), 397 (6), 209 (14), 161 (23), 127 (10), 119 (26), 104 (88), 84 (100), 77 (69). Anal. Calcd. for C_36_H_34_N_8_O_4_ (642.71): C, 67.28; H, 5.33; N, 17.43. Found: C, 67.59; H, 5.02; N, 17.09%.

#### 3.1.7. Alternate Synthesis of **10a**

A mixture of acetyltriazolopyrimidine **11** (0.269 g, l mmol) and chalcone **4d** (0.349 g, 1 mmol) in ethanol (15 mL) was refluxed for 6 h, gave product identical in all respects (m.p., mixed m.p. and IR spectra) with compound **10a** which obtained from reaction of **7d** + **1a**.

### 3.2. Anticancer Activity

The cytotoxic evaluation of the synthesized compounds was carried out at the Regional Center for Mycology and Biotechnology at Al-Azhar University, Cairo, Egypt according to the reported method [36,37].

### 3.3. Molecular Modeling

Docking studies were performed using the MOE 2014.09 software. Regularization and optimization for protein and ligand were performed. Each docked compound was assigned a score according to its fit in the ligand binding pocket (LBP) and its binding mode [38,39,40].

## 4. Conclusions

In our present work, we present an efficient synthesis of novel morpholinylchalcones, which have not been hitherto reported. These chalcones were used as building blocks for constructing a series of pyridopyrimidinethiones and pyrido[2,3-*d*][1,2,4]triazolo[4,3-*a*]pyrimidin-5(1*H*)-ones. The structures of the newly synthesized compounds were established on the basis of spectroscopic evidences and their synthesis by alternative methods. The in vitro growth inhibitory activity of the synthesized compounds against hepatocellular carcinoma (HepG-2) and human lung cancer (A-549) cell lines were investigated in comparison with cisplatin as reference drug using MTT assays and the results revealed promising activities of four compounds. Moreover, computational studies using MOE 2014.09 software supported the biological activity results.

## Figures and Tables

**Table 1:** The in vitro inhibitory activity of tested compounds against tumor cell lines expressed as IC_50_ values (μg/mL) ± standard deviation from three replicates.

Tested Compounds	Tumor Cell Lines		Tested Compounds	Tumor Cell Lines	
	A-549	HepG2		A-549	HepG2
**4a**	16.3 ± 1.31	21.1 ± 0.91	**7c**	6.79 ± 1.11	8.42 ± 1.15
**4c**	24.0 ± 1.21	20.0 ± 1.23	**8**	2.78 ± 0.86	29.9 ± 0.93
**4e**	5.37 ± 0.95	15.68 ± 1.12	**10a**	24.47 ± 1.23	27.68 ± 1.31
**4f**	7.38 ± 0.82	9.78 ± 0.78	**10b**	26.8 ± 0.75	17.7 ± 0.73
**5**	10.3 ± 0.91	12.4 ± 0.98	**10c**	15.2 ± 1.42	14.9 ± 1.14
**7a**	9.41 ± 0.79	13.9 ± 0.77	**10d**	12.2 ± 0.88	8.72 ± 0.89
**7b**	5.7 ± 0.91	3.54 ± 1.11	Cisplatin	0.95 ± 0.9	1.4 ± 1.1

**Table 2:** Bioactivity and ADME toxicity.

Compound	8
Molecular weight	472.56 g/mol
Num. rotatable bonds	5
Num. H-bond acceptors	4
Num. H-bond donors	3
TPSA	130.49 Å^2^
GI absorption	High
BBB permeant	No
P-gp substrate	Yes
CYP1A2 inhibitor	No
Log Kp (skin permeation)	−6.43 cm/s
Lipinski	Yes; 0 violation
PAINS	0 alert
Leadlikeness	No; 2 violations: MW >350, XLOGP3 >3.5
Synthetic accessibility	3.76

Topological polar surface area (TPSA), gastrointestinal absorption (GI absorption), blood brain barrier (BBB) permeant, P-glycoprotein substrate (P-gp substrate), cytochrome P50 1A2 inhibitor (CYP1A2 inhibitor), pan-assay interference structure (PAINS).

## Supplementary Materials

Supplementary materials are available online.
