# Supplementary material for: Synthesis, Antitumor Evaluation and Molecular Docking of New Morpholine Based Heterocycles
Source: Molecules. 2017 Jul 20;22(7):1211. doi: 10.3390/molecules22071211 (PMC6152077; doi:10.3390/molecules22071211)
Supplement: Supplementary file 1 [file molecules-22-01211-s001.docx]

Synthesis, Antitumor Evaluation and Molecular Docking of New Morpholine Based Heterocycles

Zeinab A. Muhammad ^1^, Mastoura M. Edrees ^1,2^, Rasha A. M. Faty ^3^, Sobhi M. Gomha ^3,^*, Seham S. Alterary ^4^ and Yahia N. Mabkhot ^4,^*

^1^ Department of Organic Chemistry, National Organization for Drug Control and Research (NODCAR), Giza 12311, Egypt; [zeinab.a.muhammad@gmail.com](mailto:zeinab.a.muhammad@gmail.com) (Z.A.M.), [mmohamededrees@yahoo.com](mailto:mmohamededrees@yahoo.com) (M.M.E.)

^2^ Department of Chemistry, Faculty of Science, King Khalid University, Abha, 61413, Saudi Arabia

^3^ Department of Chemistry, Faculty of Science, Cairo University, Giza 12613, Egypt; [rashafaty@yahoo.com](mailto:rashafaty@yahoo.com)

^4^ Department of Chemistry, College of Science, King Saud University, P. O. Box 2455, Riyadh-11451, Saudi Arabia; salterary@ksu.edu.sa

***** Correspondence: [s.m.gomha@gmail.com](mailto:s.m.gomha@gmail.com) (S.M.G.); Tel.: +20-237-400-304 (S.M.G.); Fax: +2-025-685-799 (S.M.G.); [yahia@ksu.edu.sa](mailto:yahia@ksu.edu.sa) (Y.N.M.); +966-11-467-5898 (Y.N.M.); +966-11-467-5992 (S.M.G.)


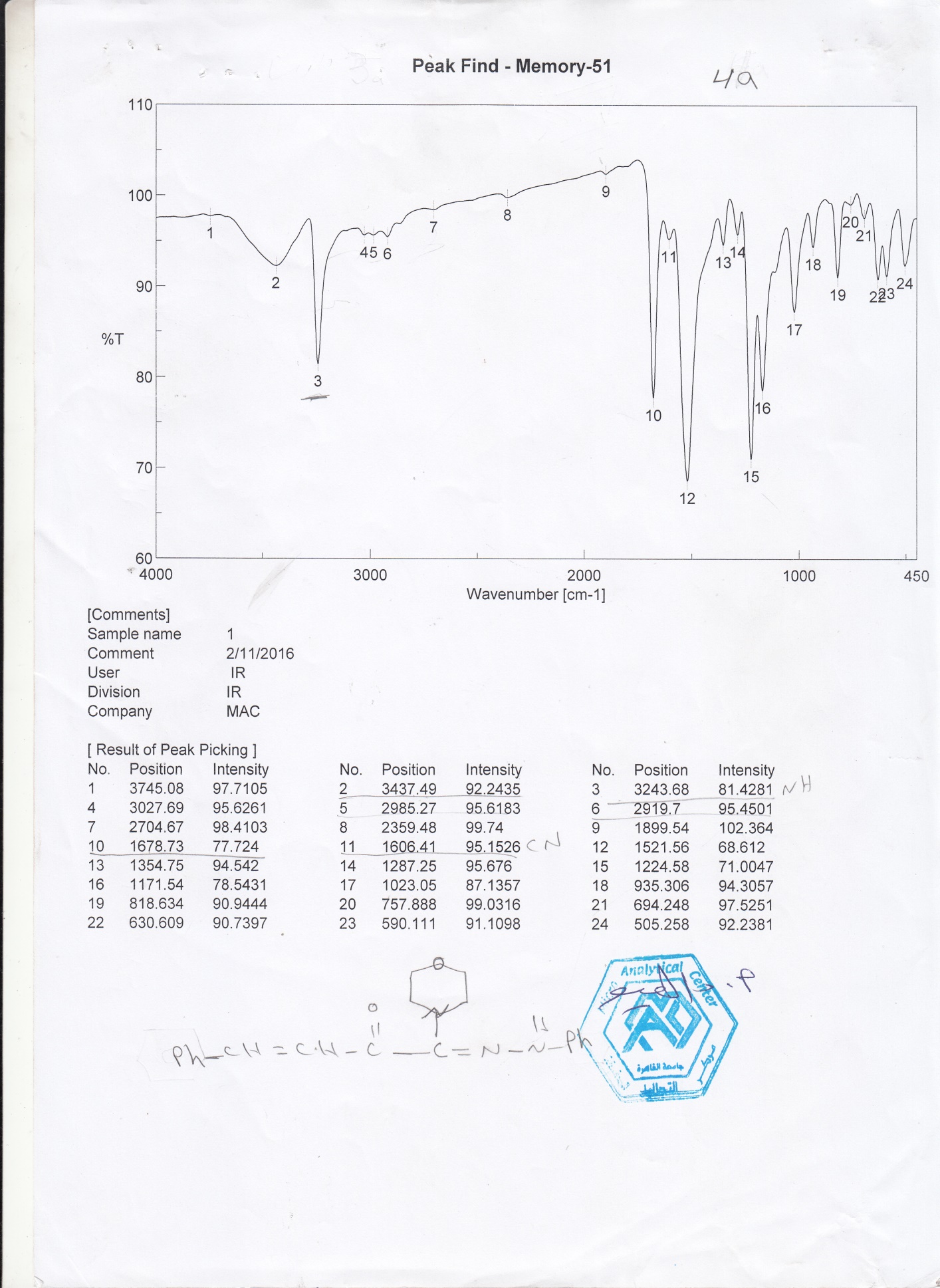


Compound **4a** (IR)


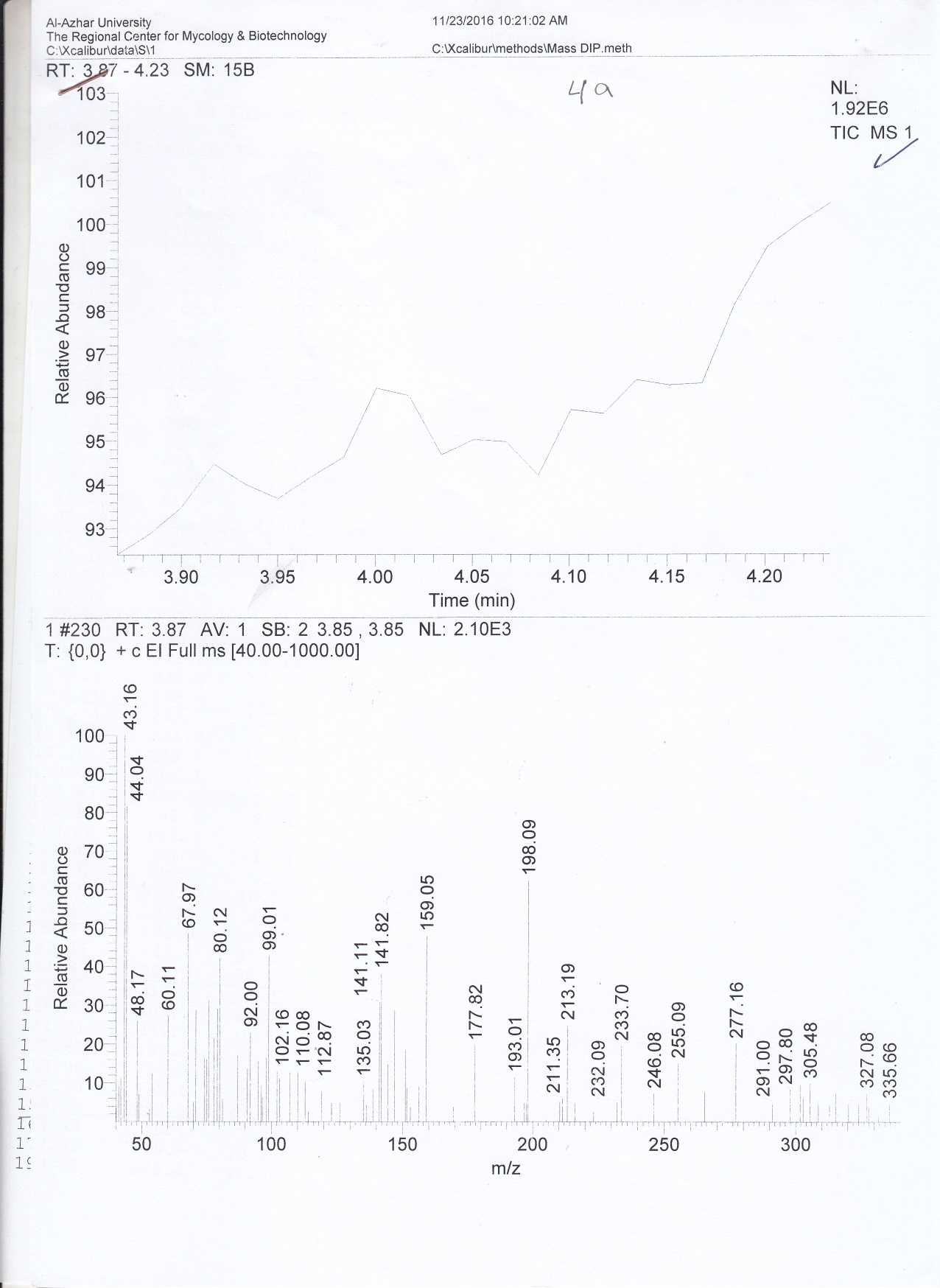


Compound **4a** (mass)


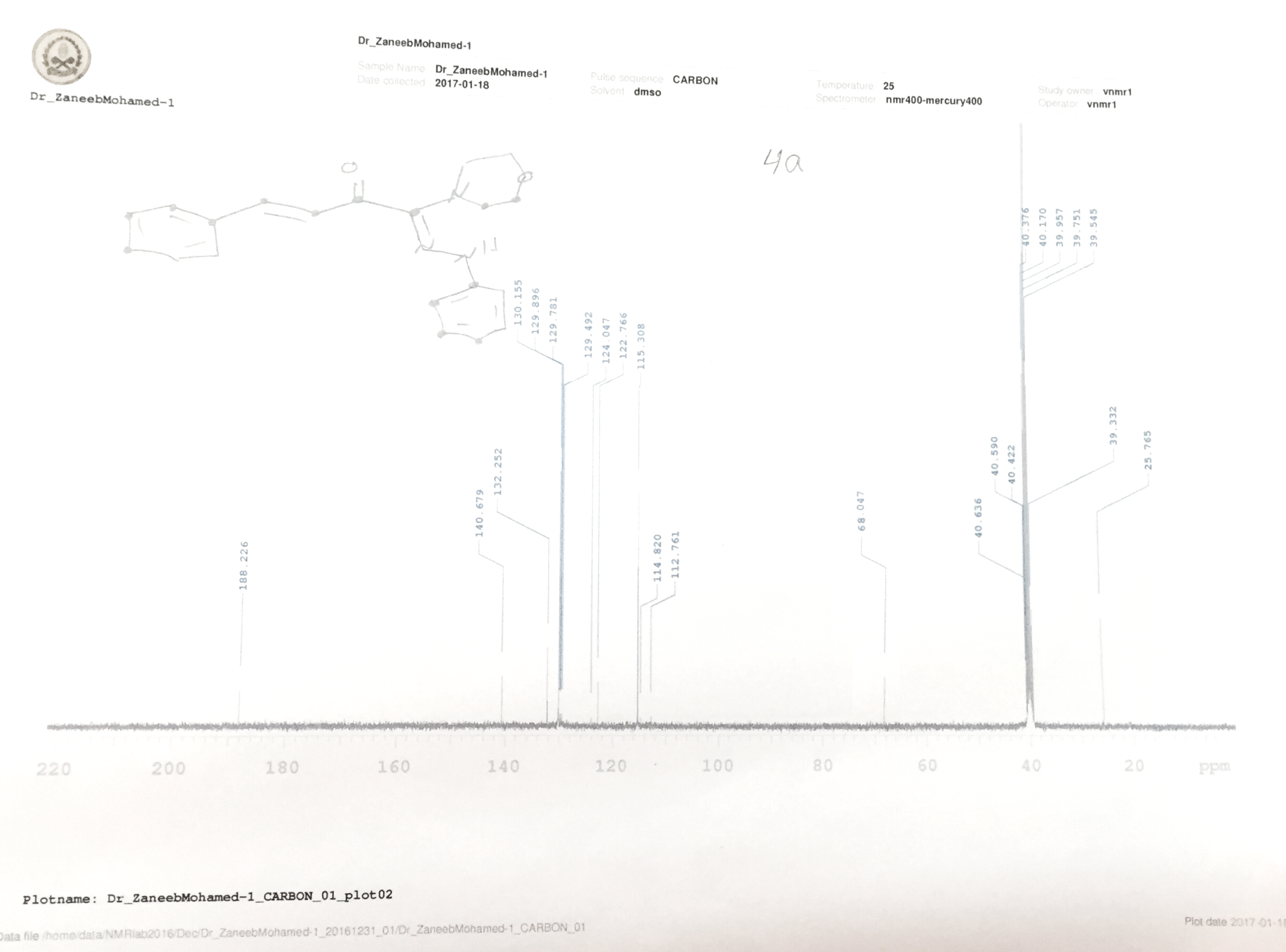


Compound **4a** (^13^C-NMR)


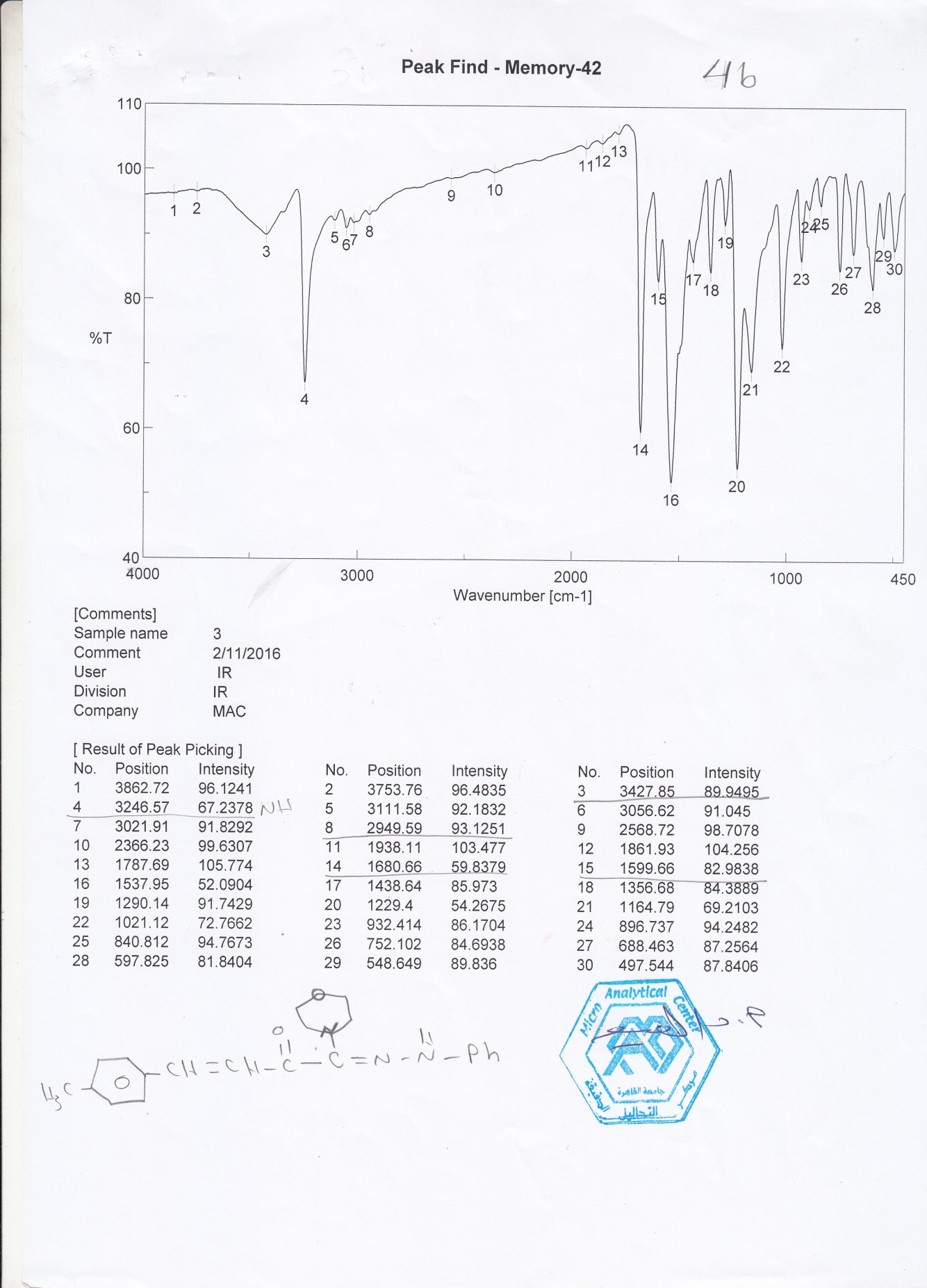


Compound **4b** (IR)


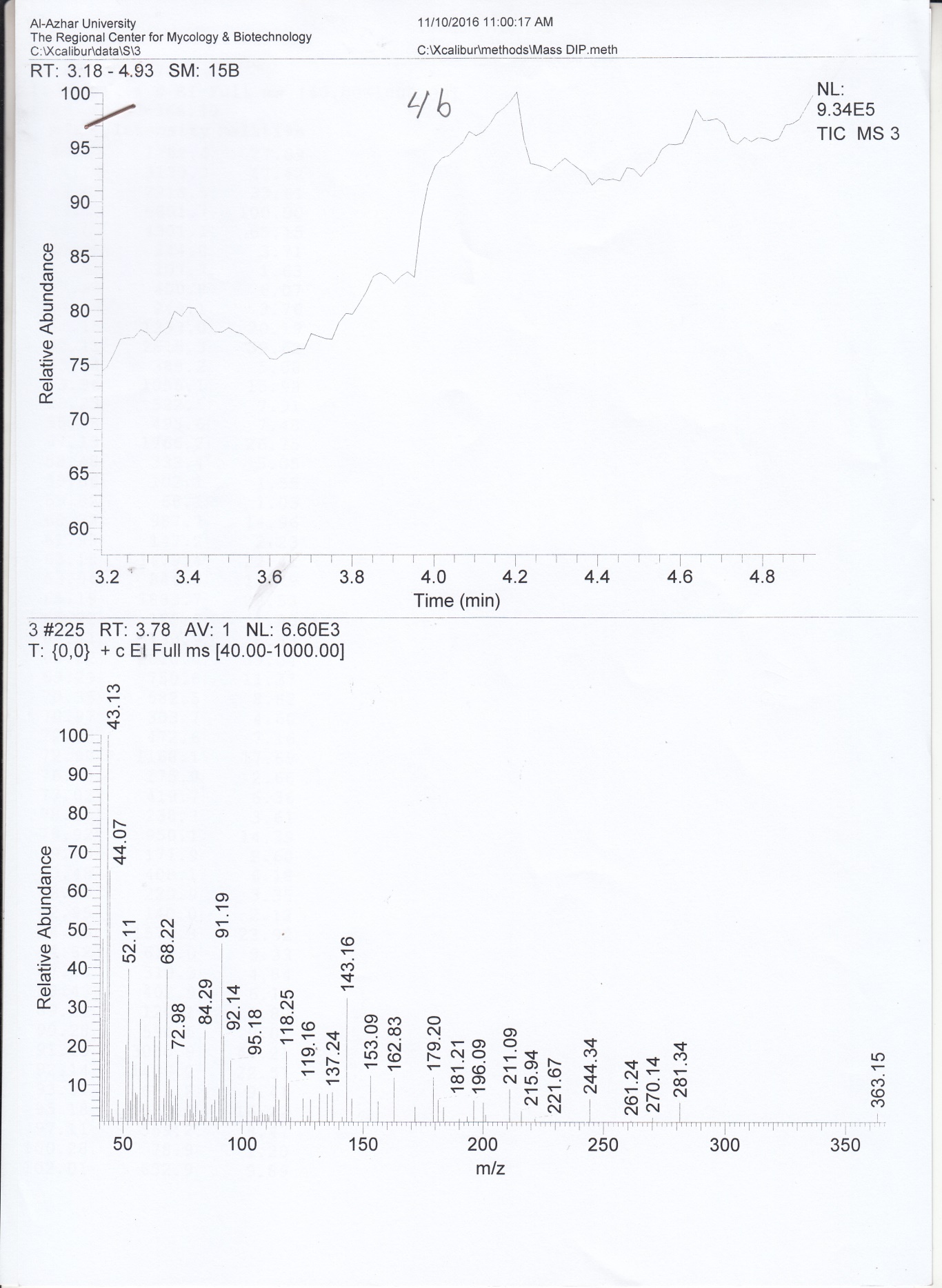


Compound **4b** (Mass)


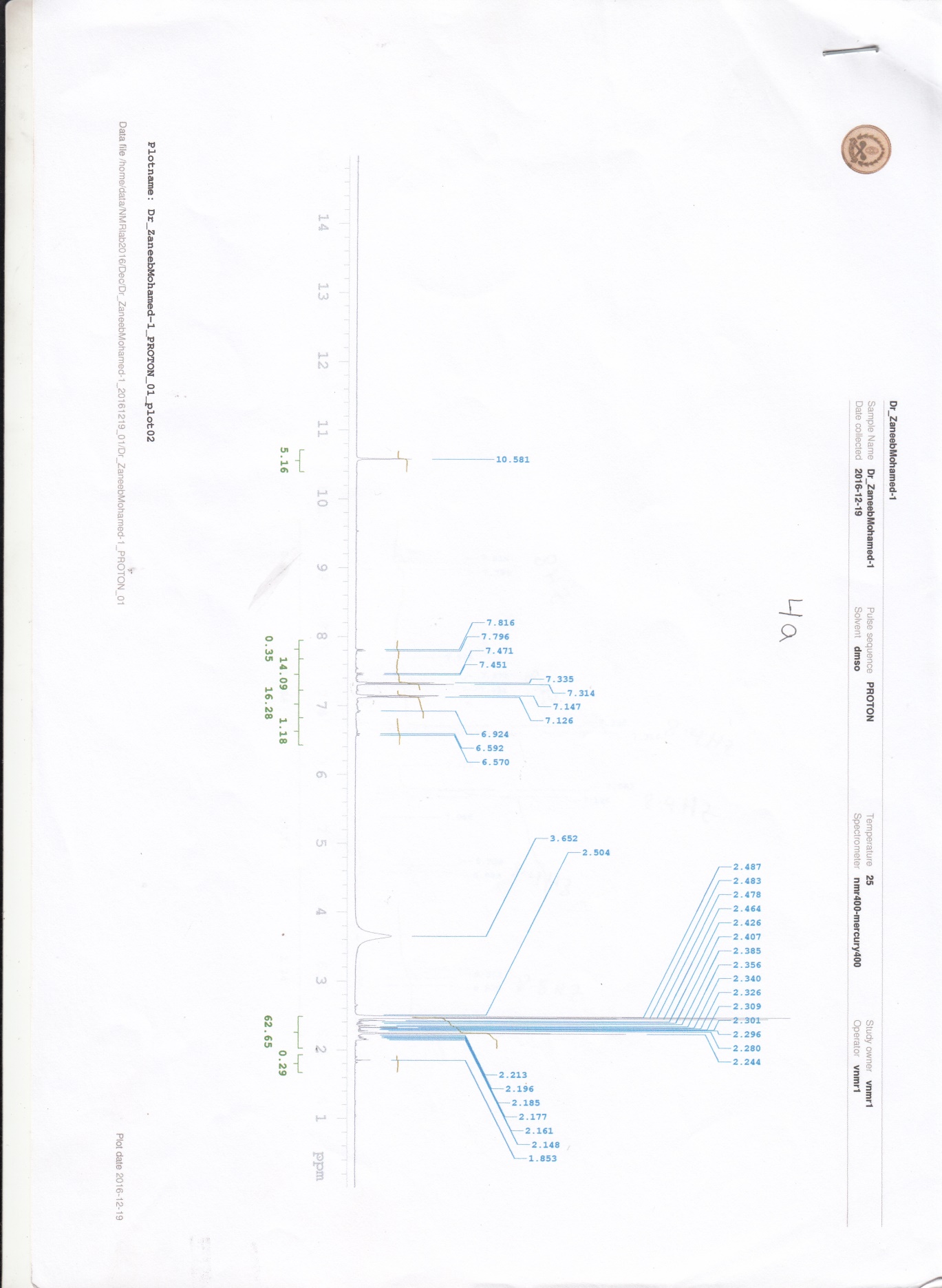


Compound **4a** (^1^H NMR)


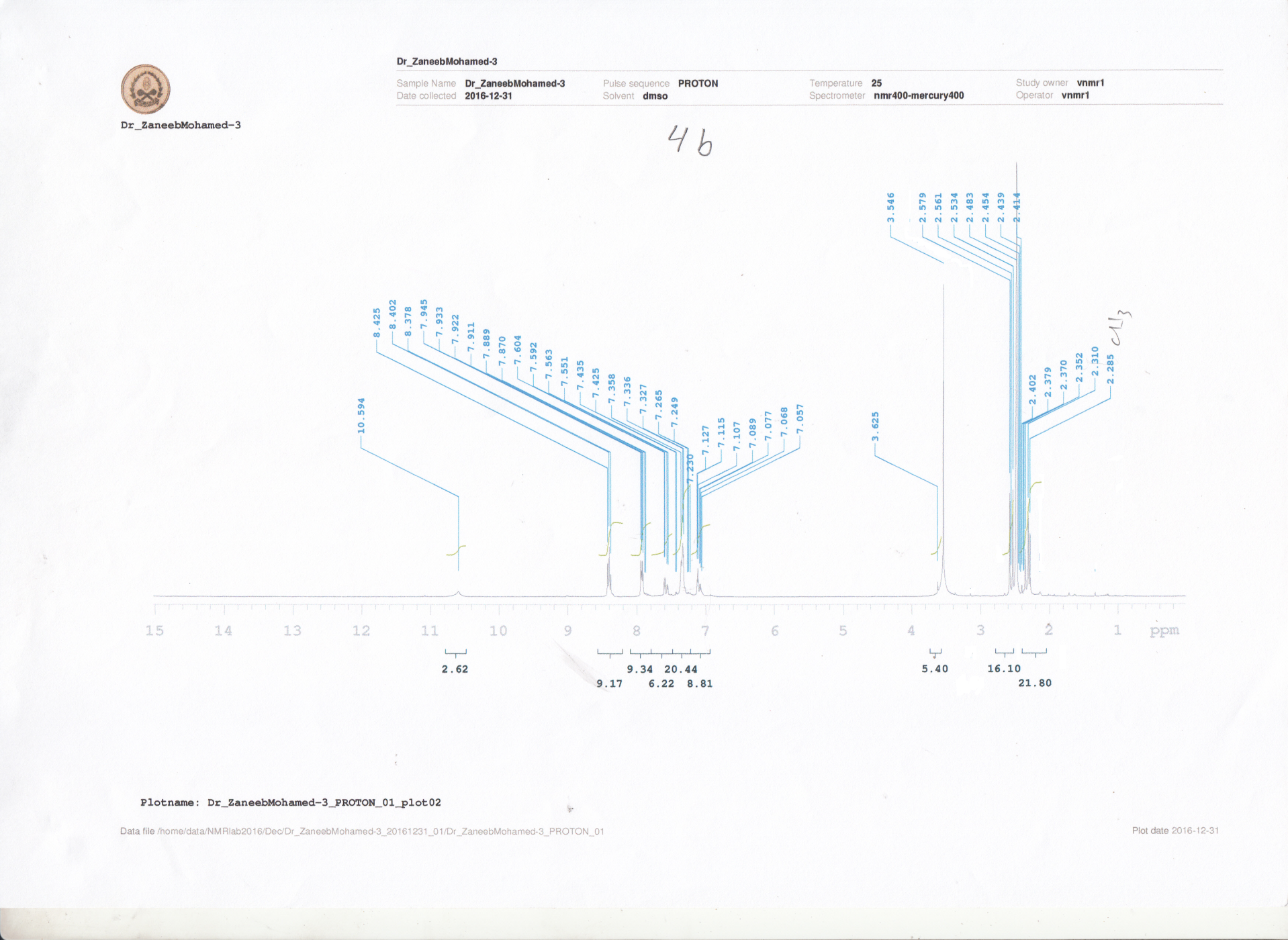


Compound **4b** (^1^H NMR)


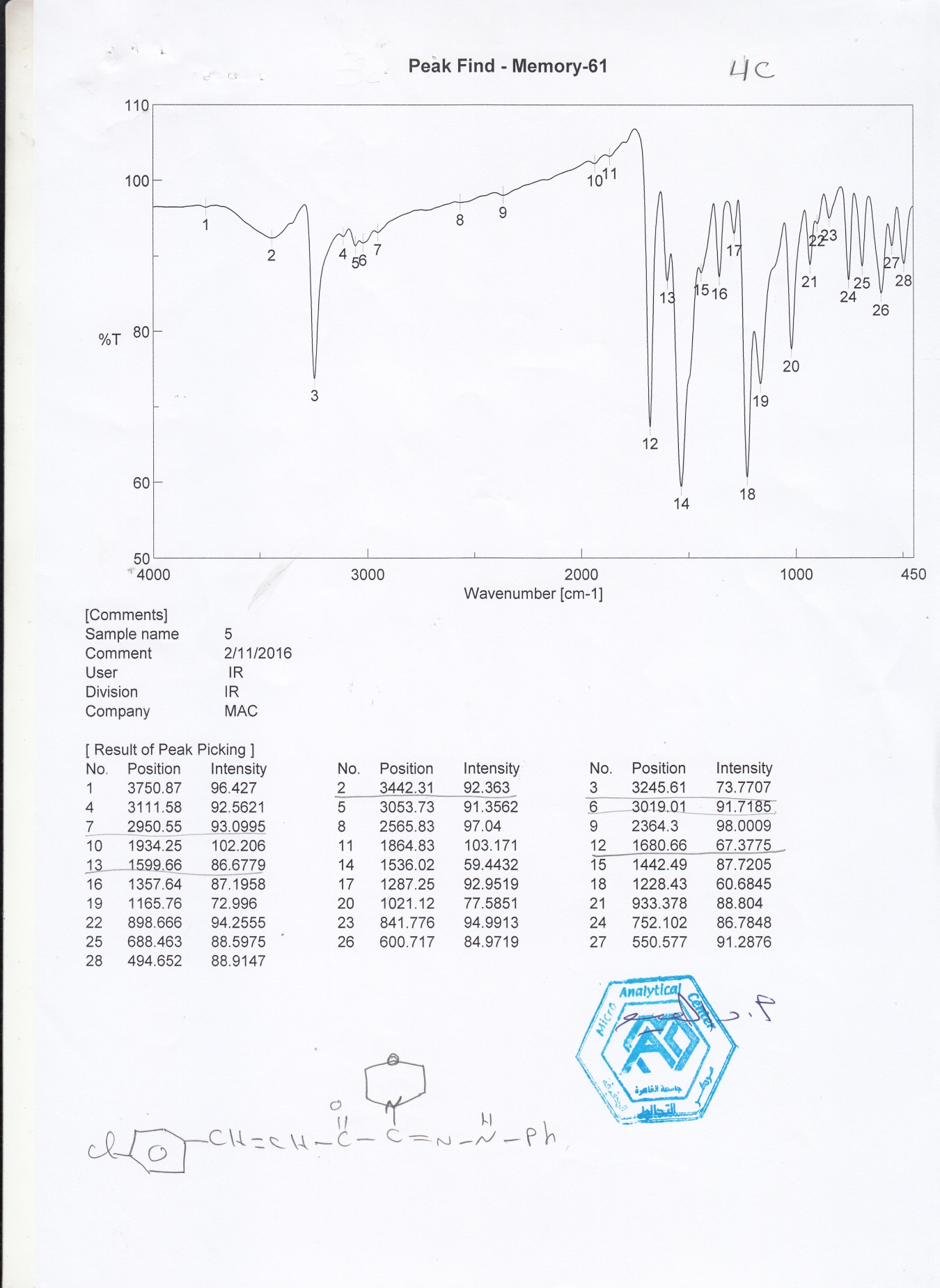


Compound **4c** (IR)


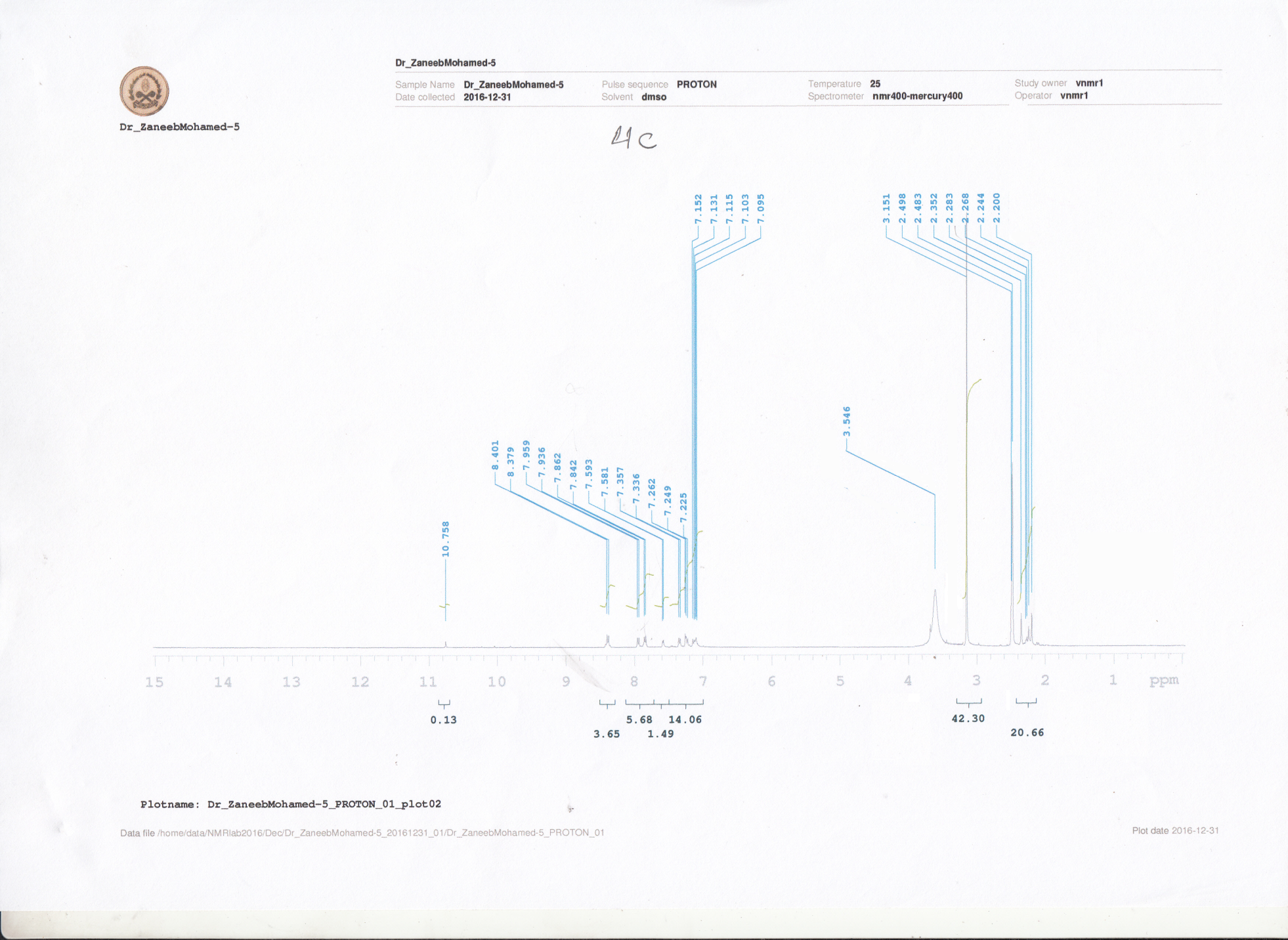


Compound **4c** (^1^H NMR)


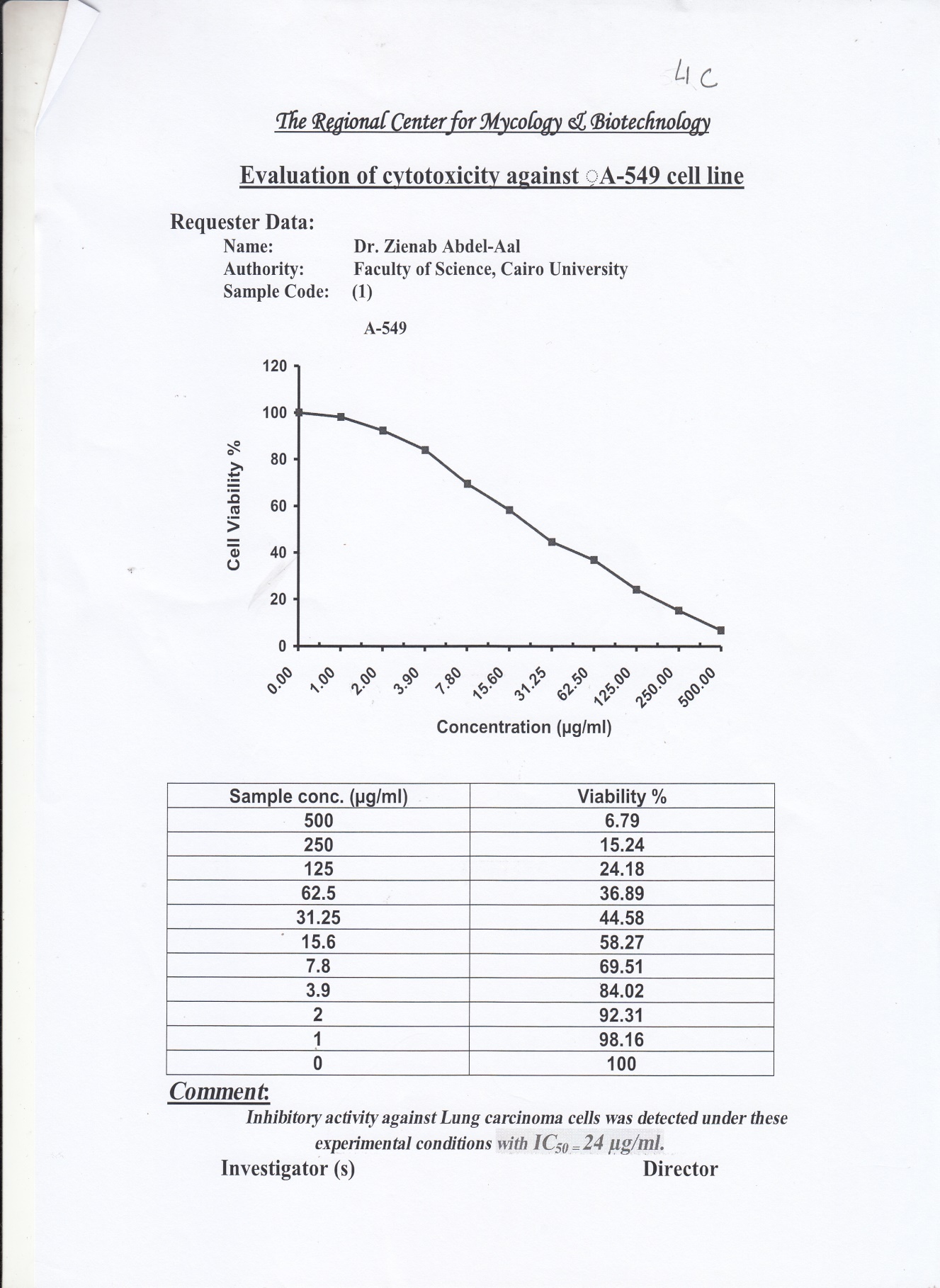


Compound **4c** (Cytotoxic activity against A-549)


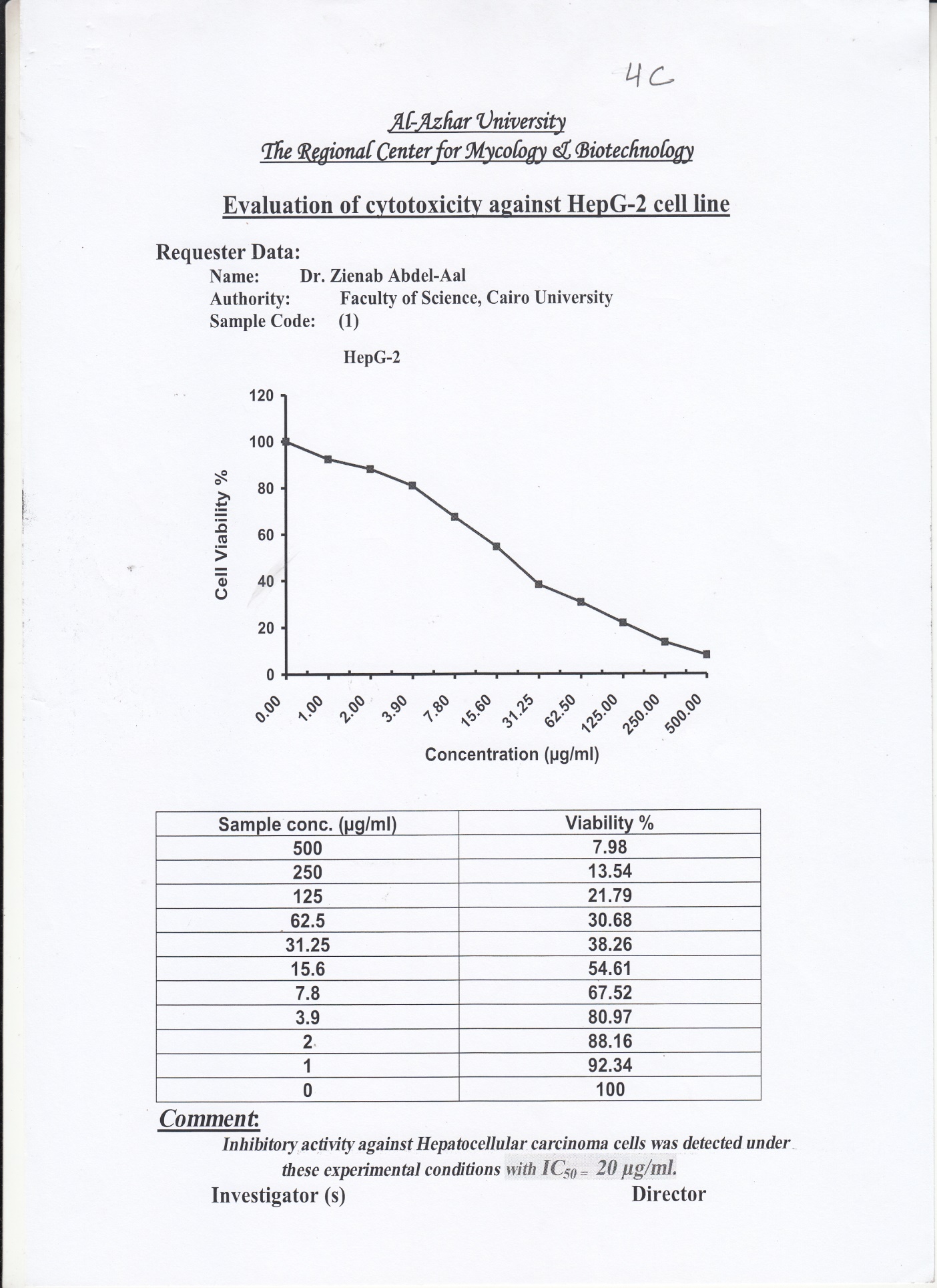


Compound **4c** (Cytotoxic activity against HepG-2)


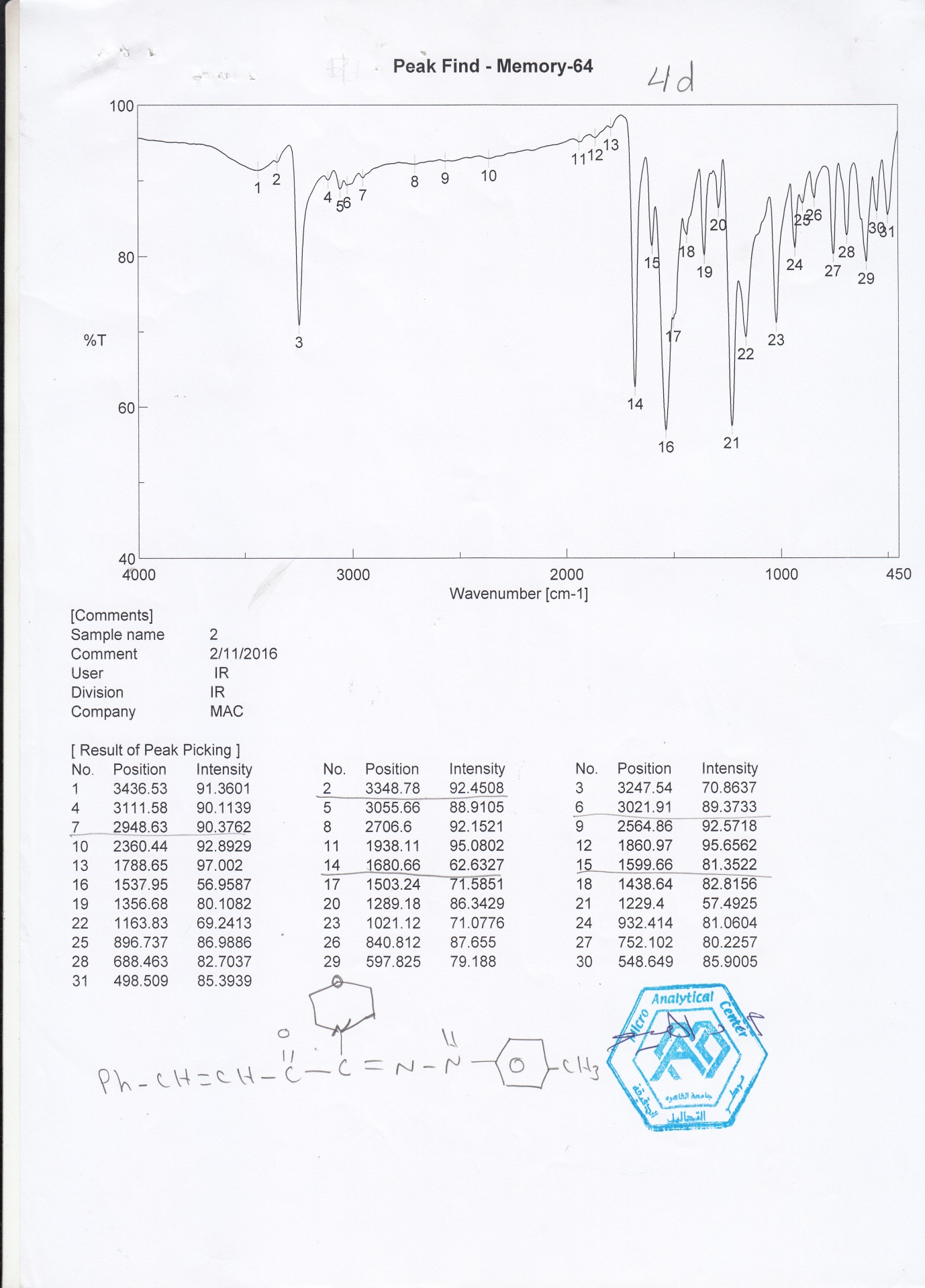


Compound **4d** (IR)


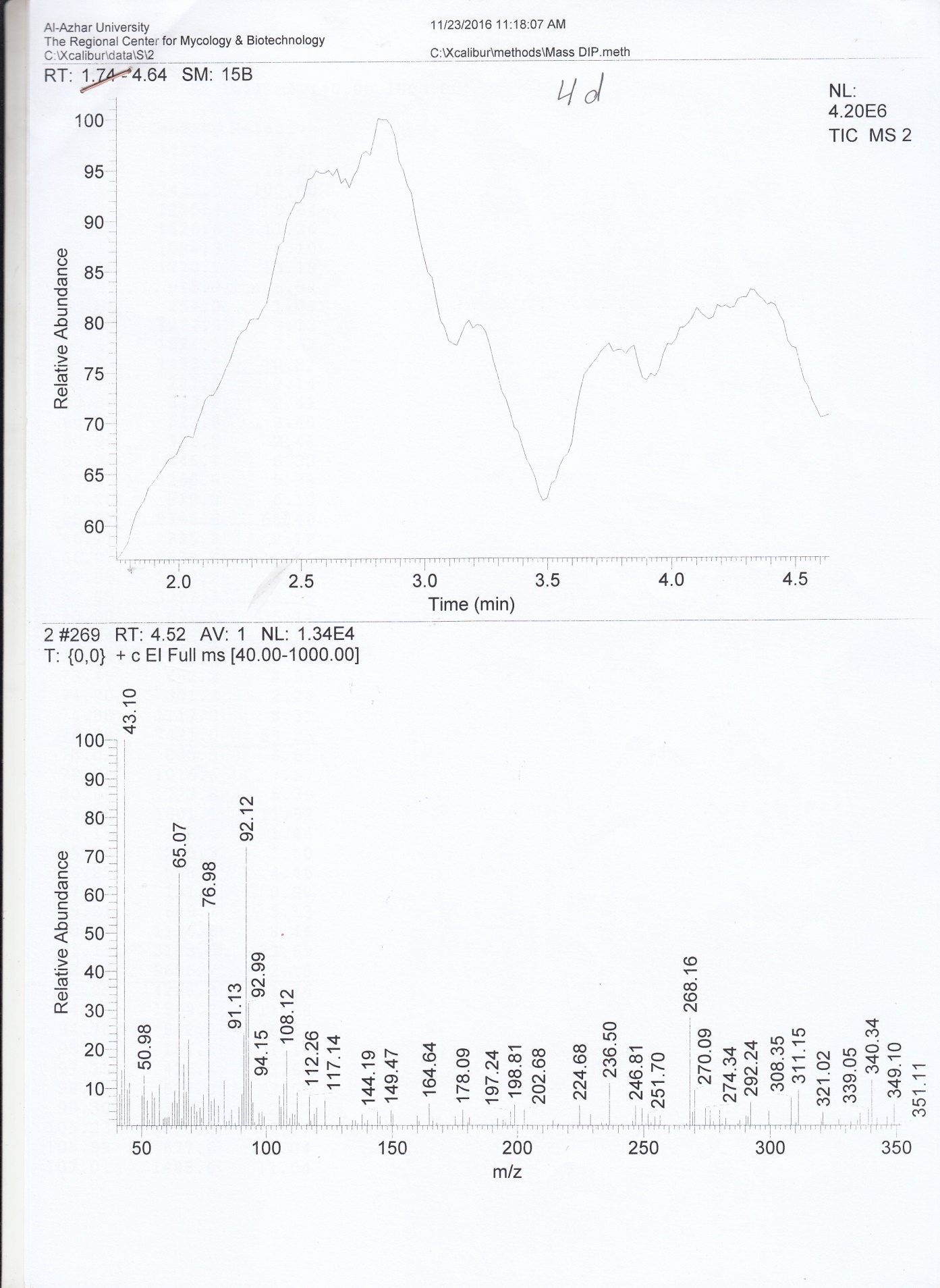


Compound **4d** (IR)


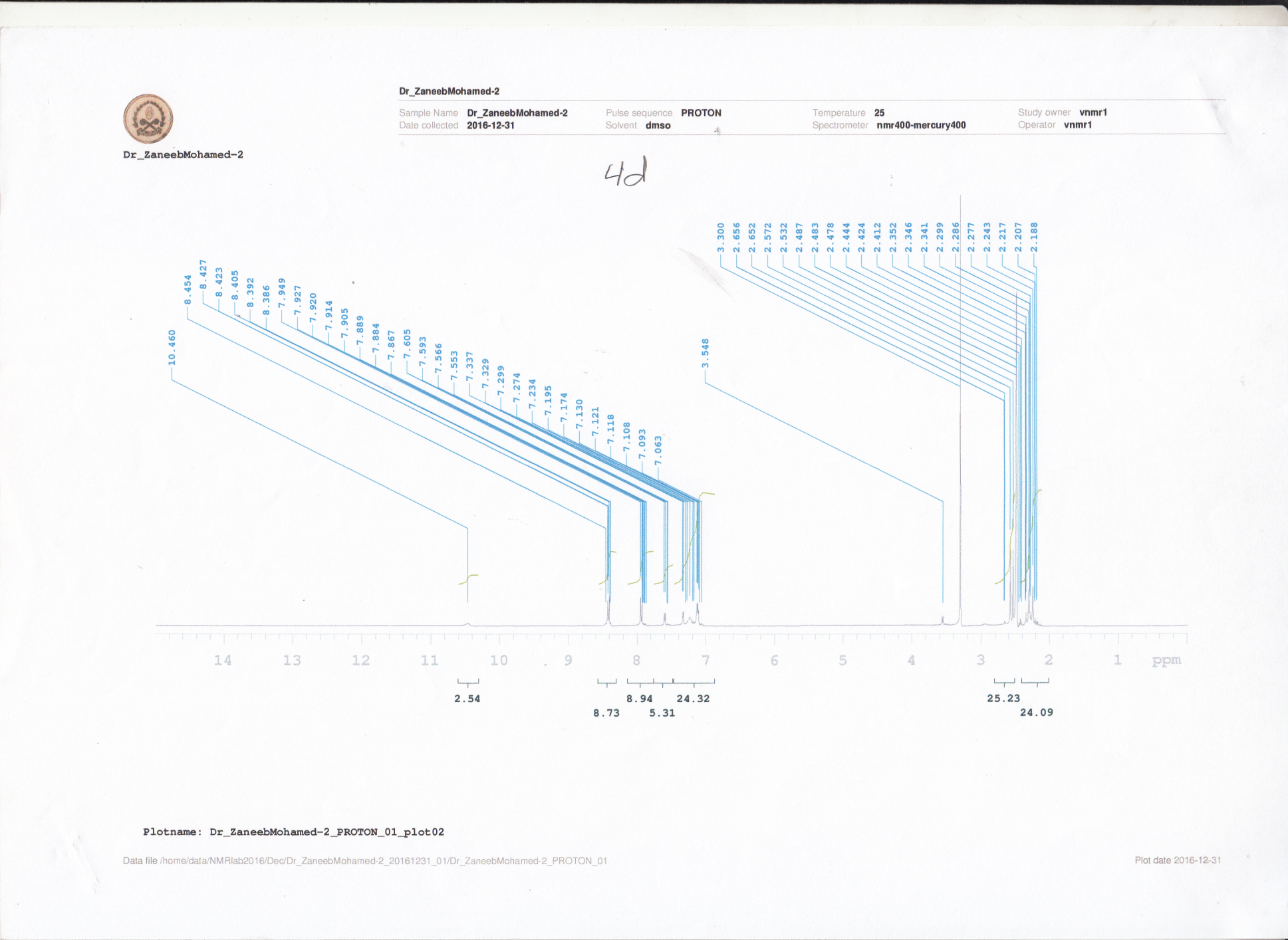


Compound **4d** (^1^H NMR).


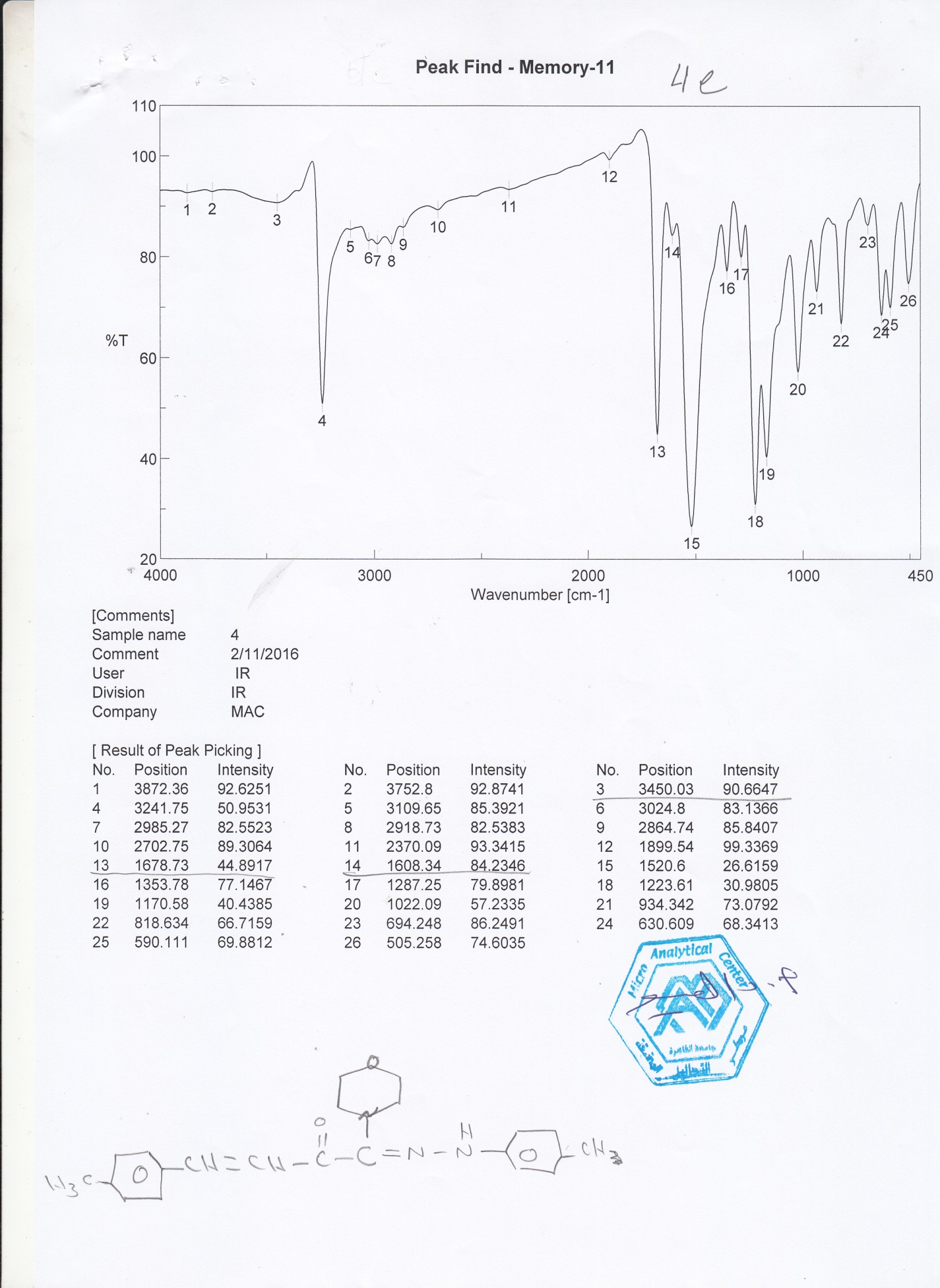


Compound **4e** (IR)


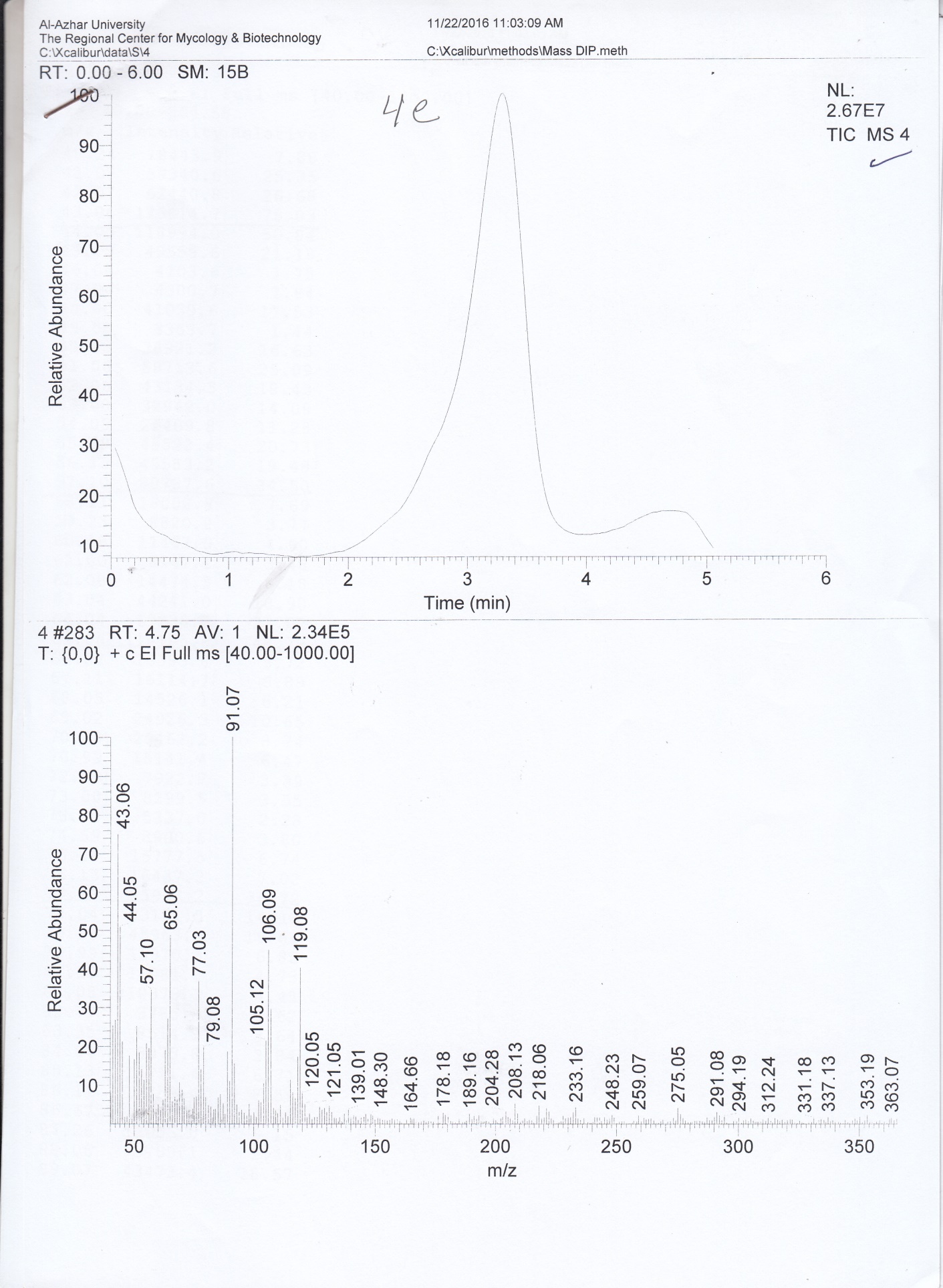


Compound **4e** (mass)


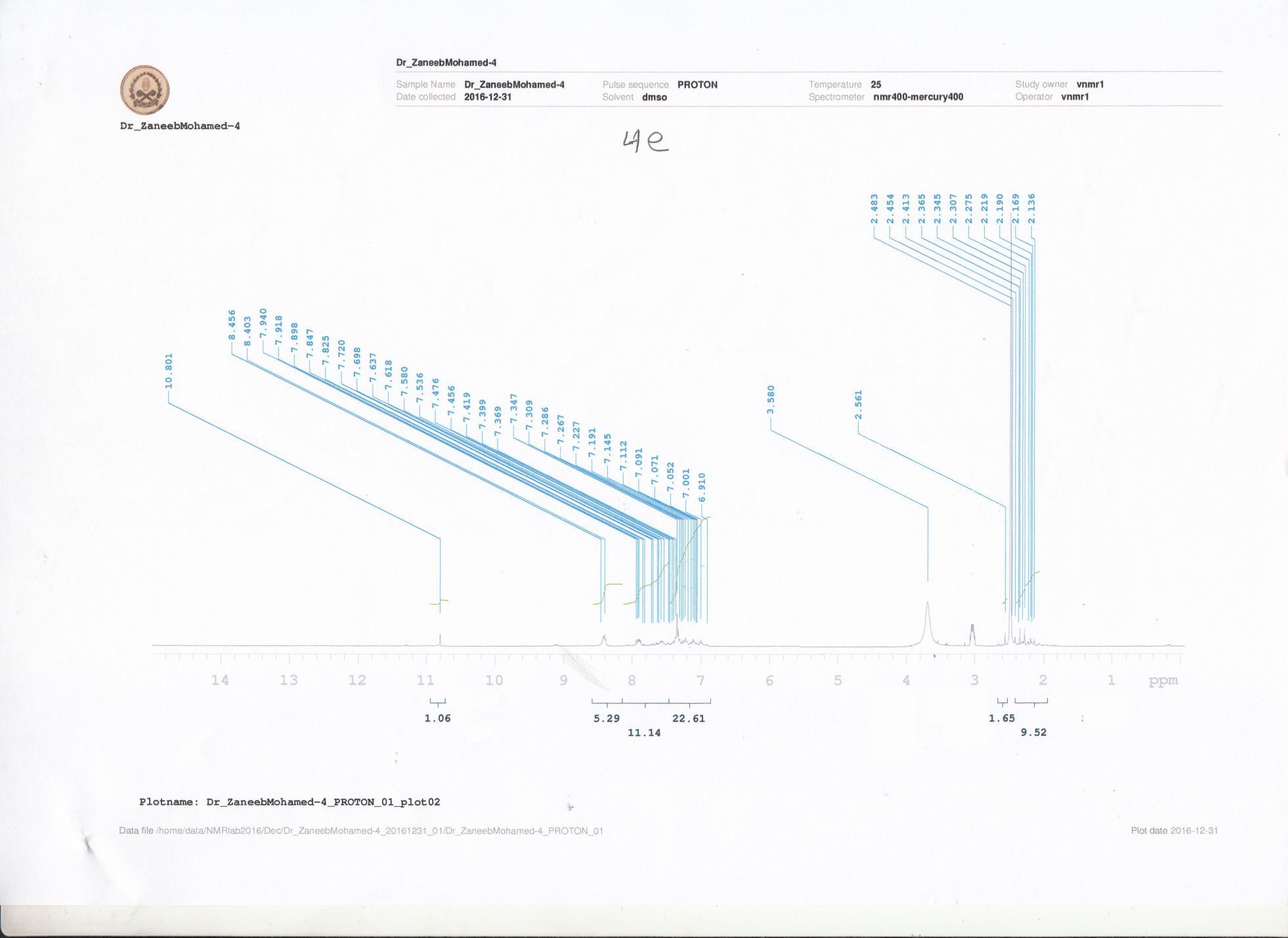


Compound **4e** (^1^H NMR)


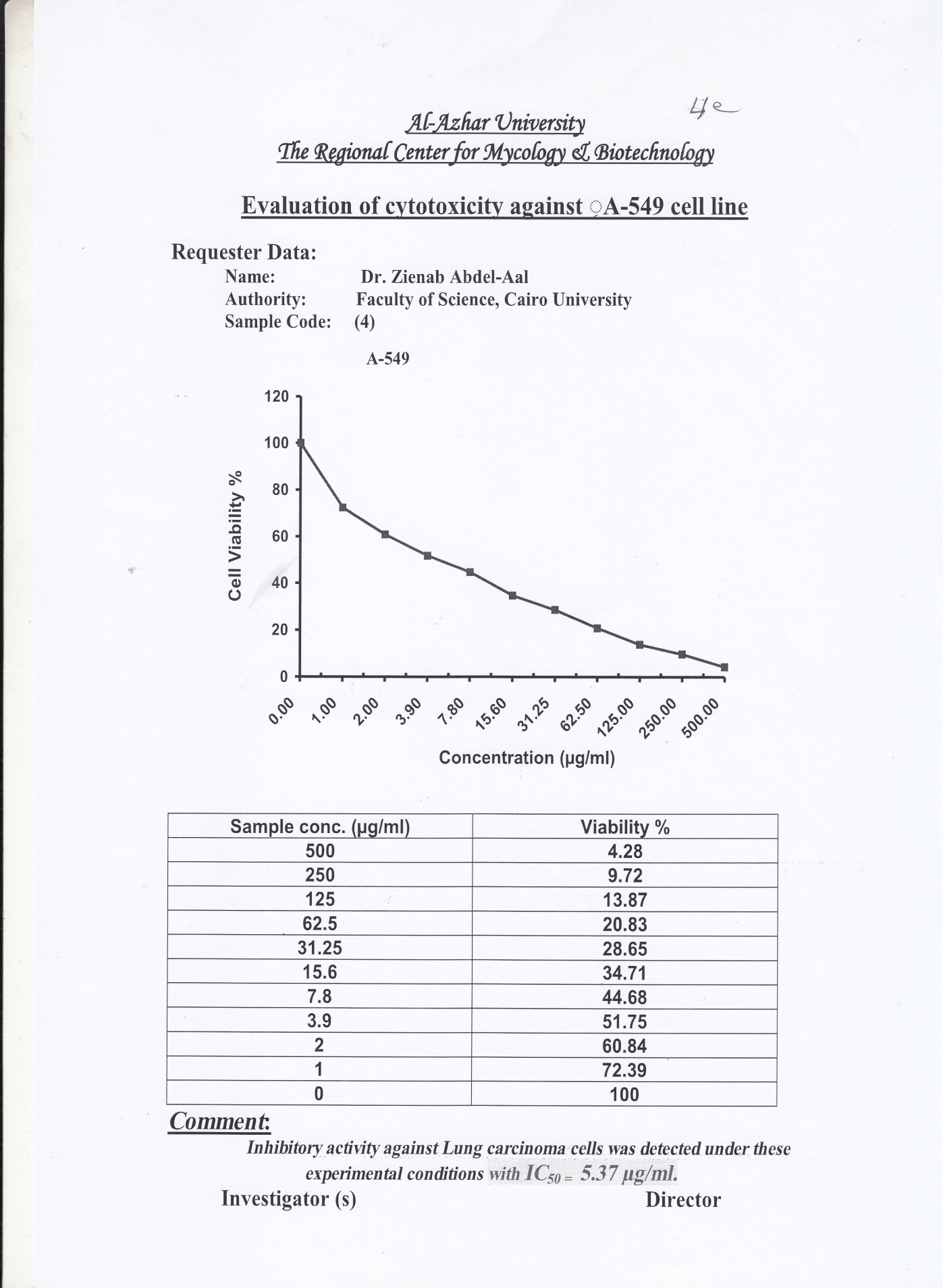


Compound **4e** (Cytotoxic activity against A-549)


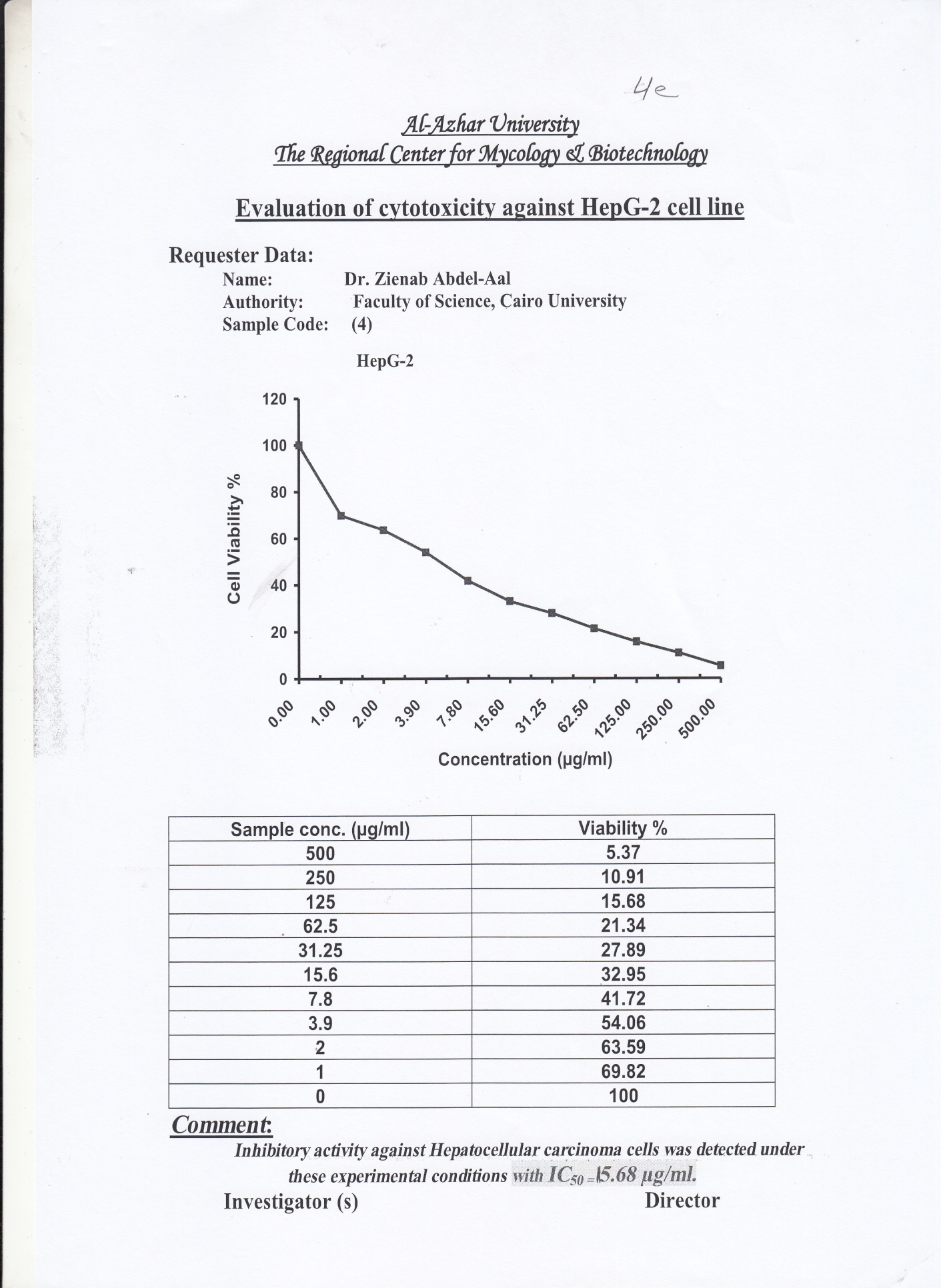


Compound **4e** (Cytotoxic activity against HepG-2)


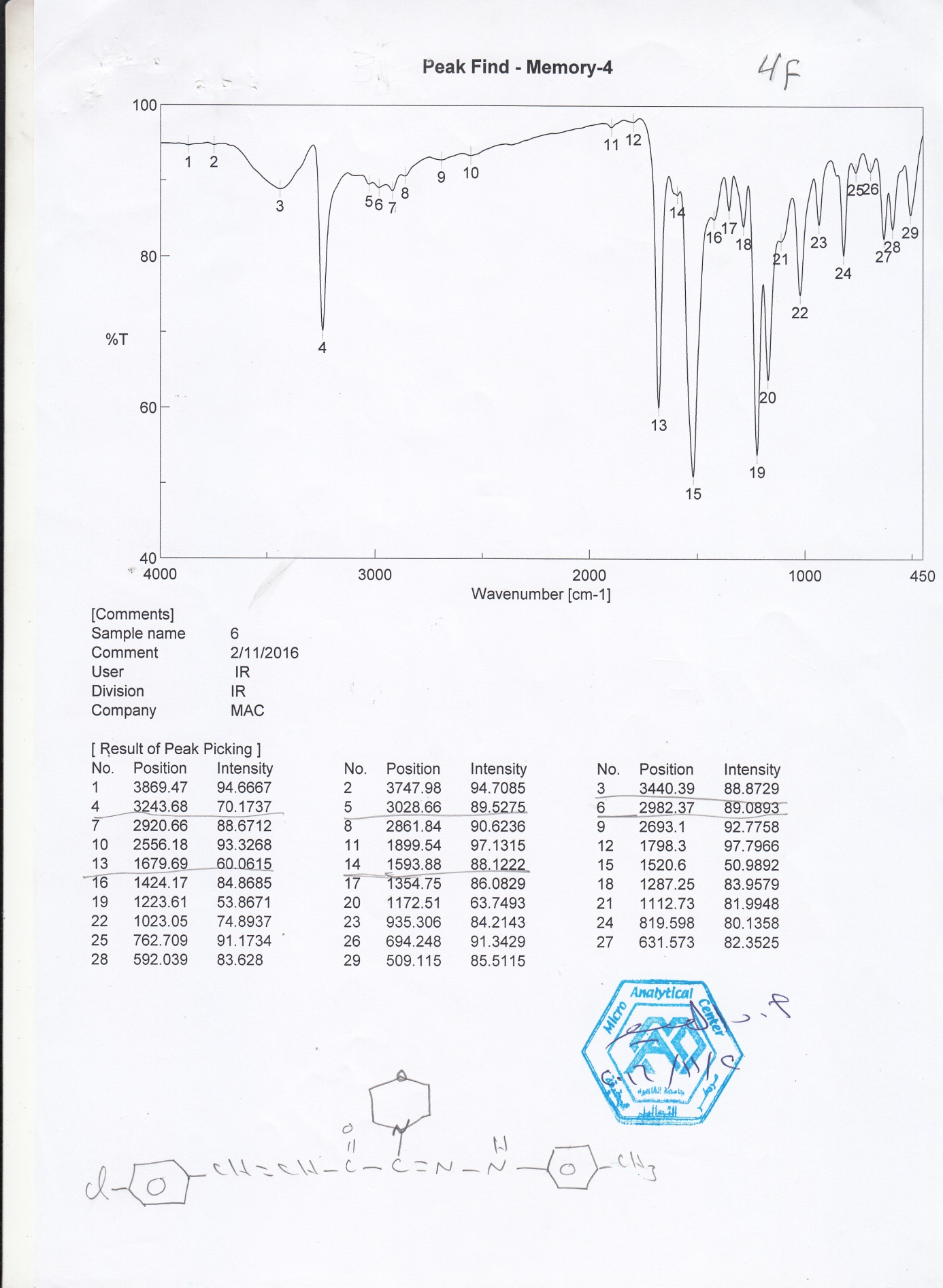


Compound **4f** (IR)


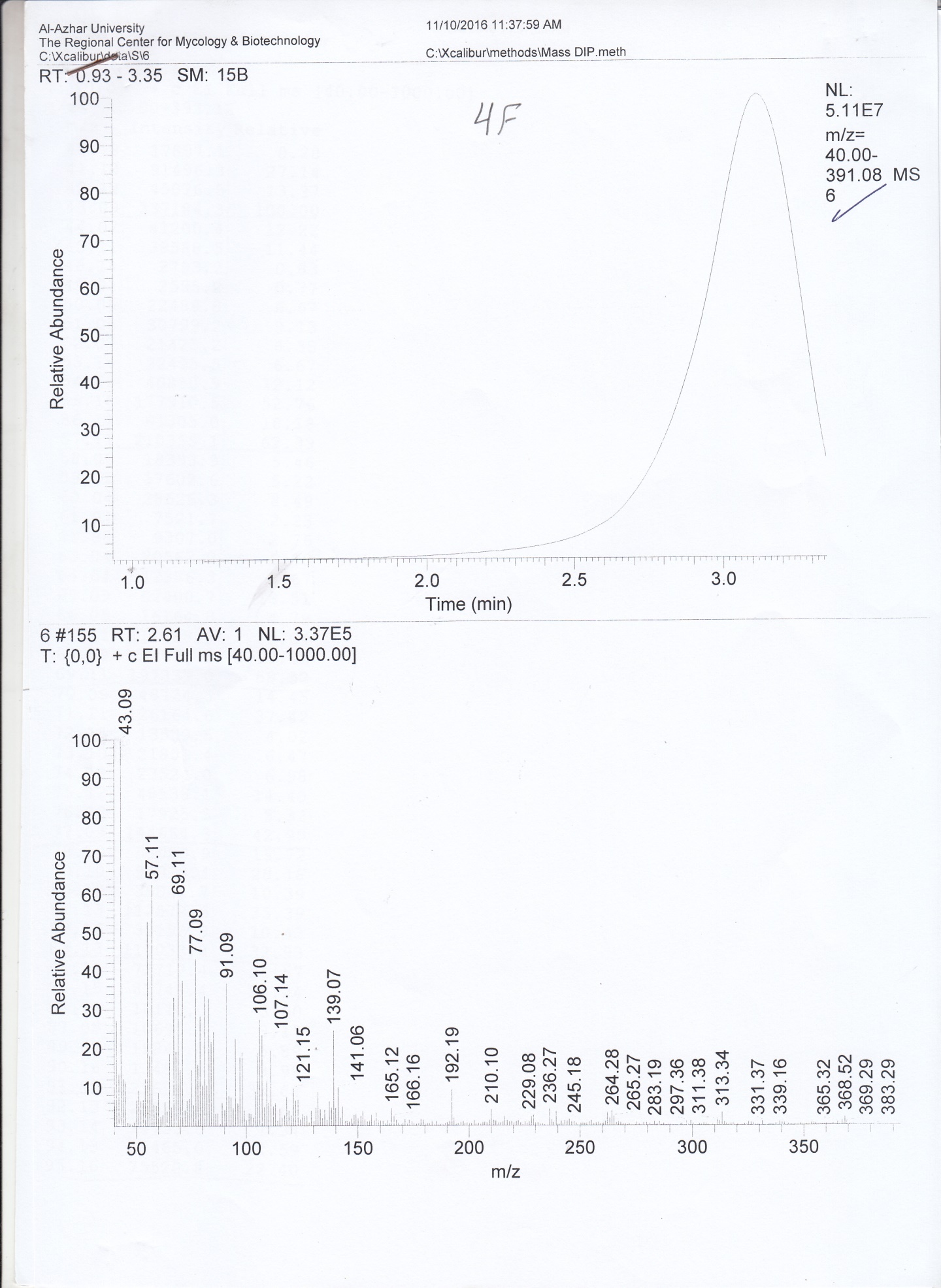


Compound **4f** (Mass)


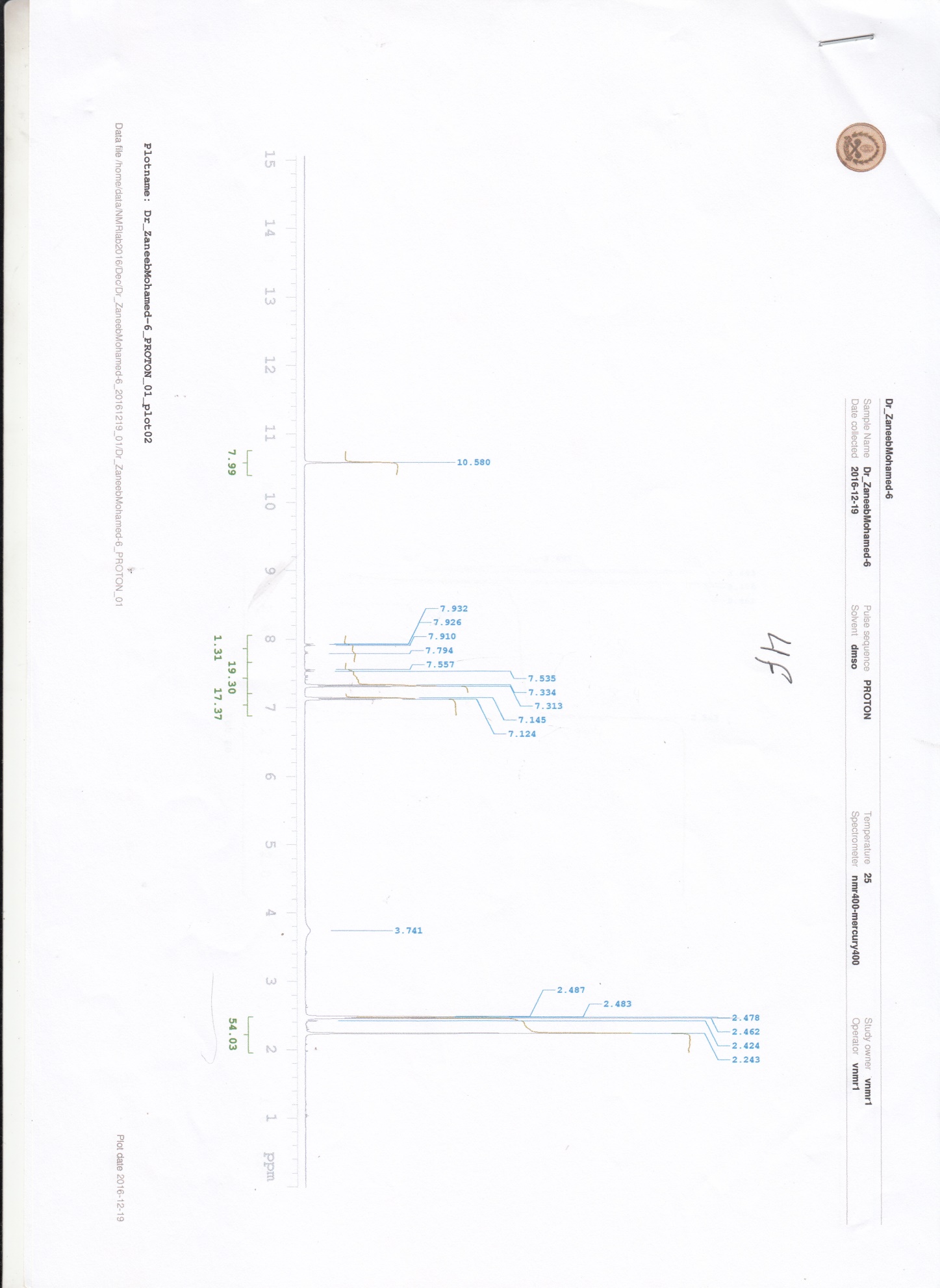


Compound **4f** (^1^HNMR)


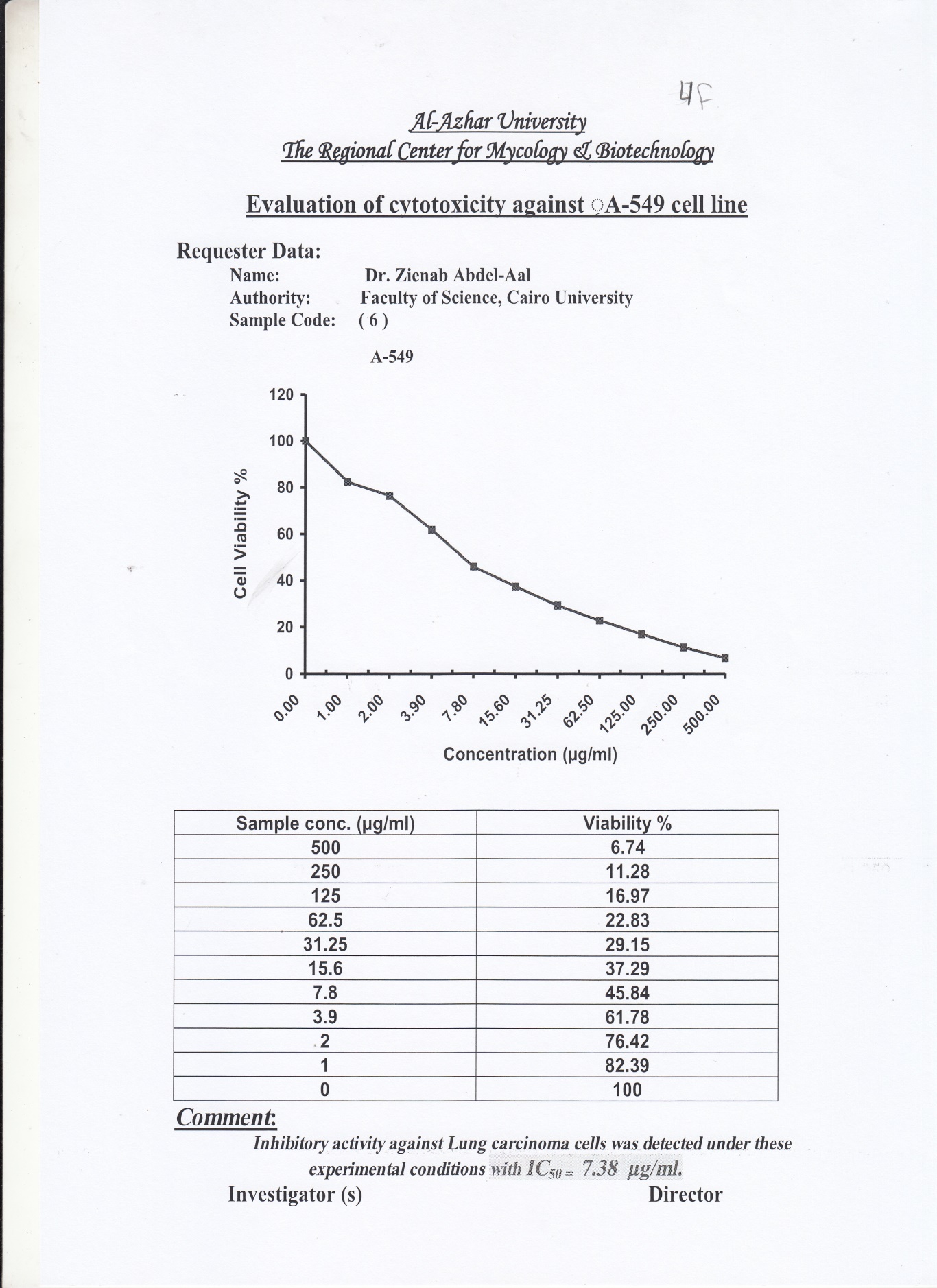


Compound **4f** (Cytotoxic activity against A-549)


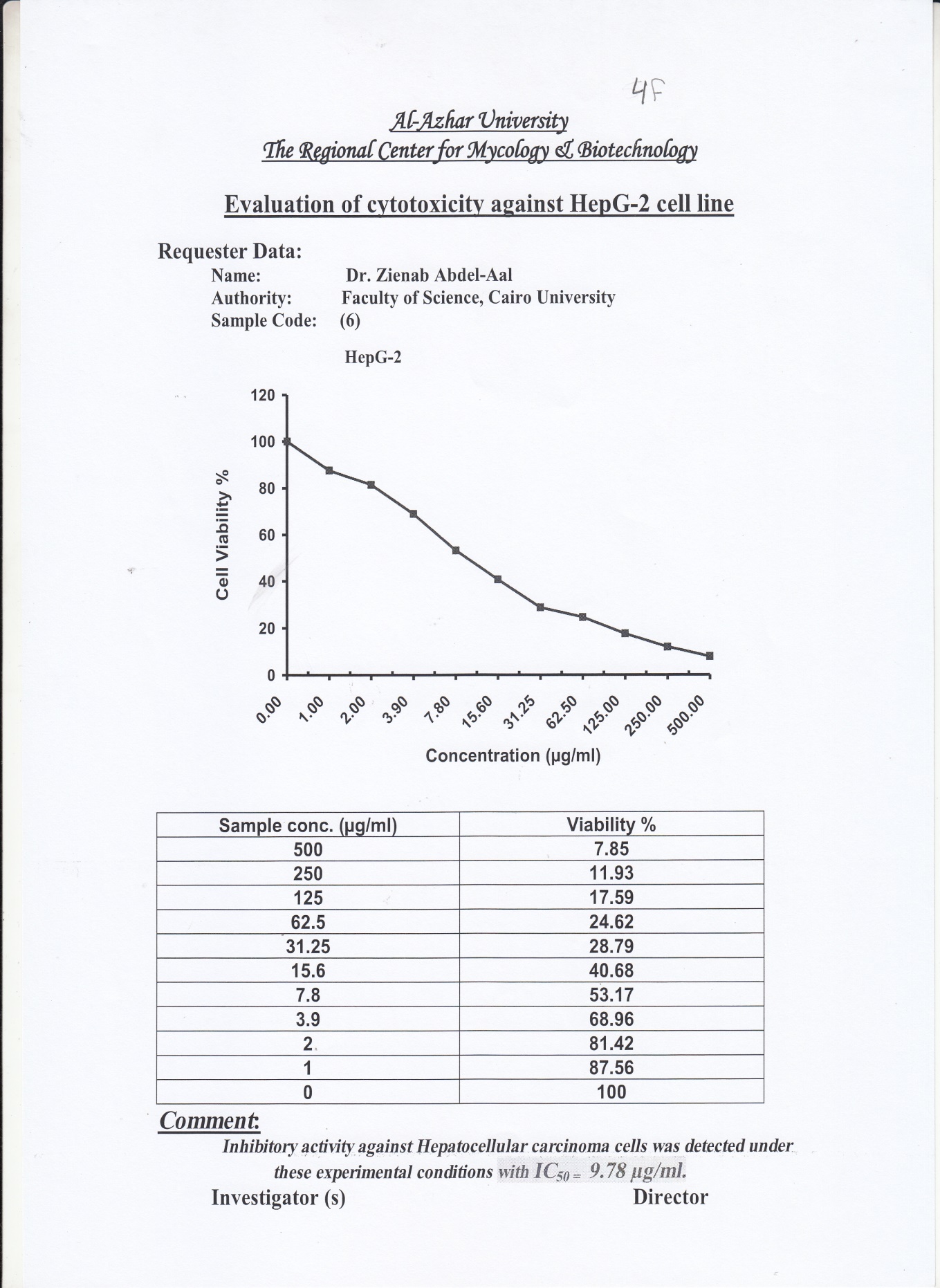


Compound **4f** (Cytotoxic activity against HepG-2)


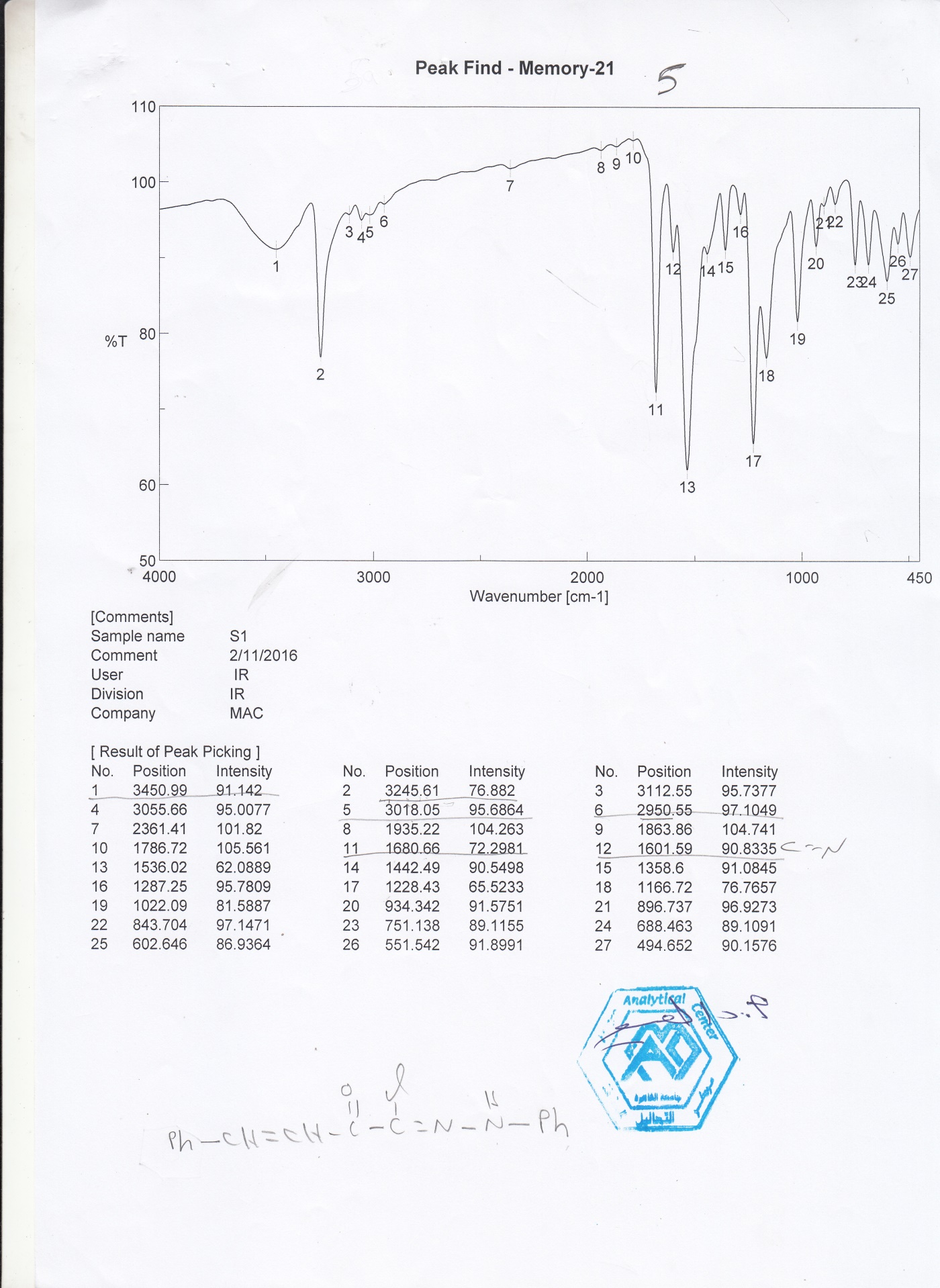


Compound **5** (IR)


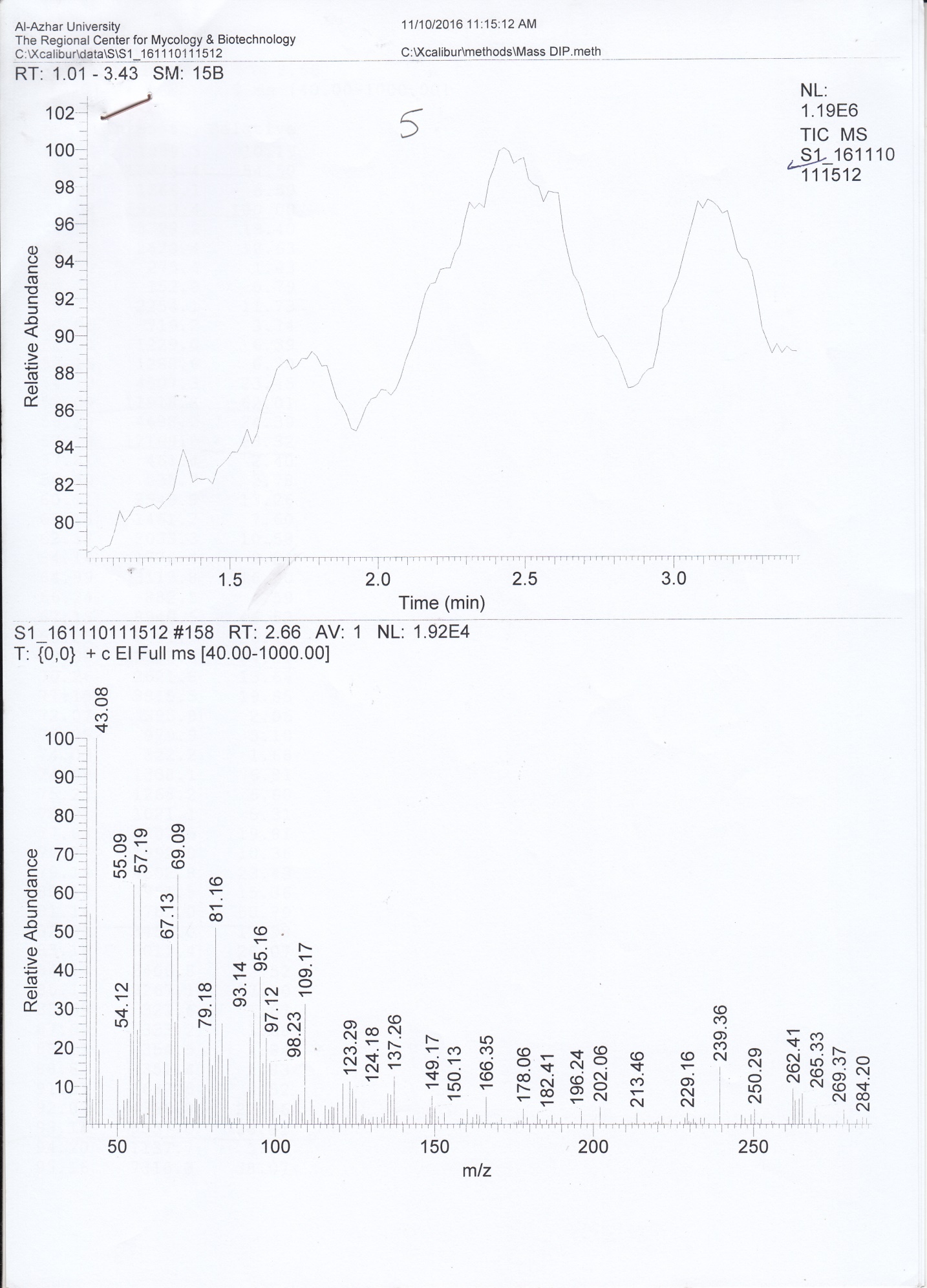


Compound **5** (mass)


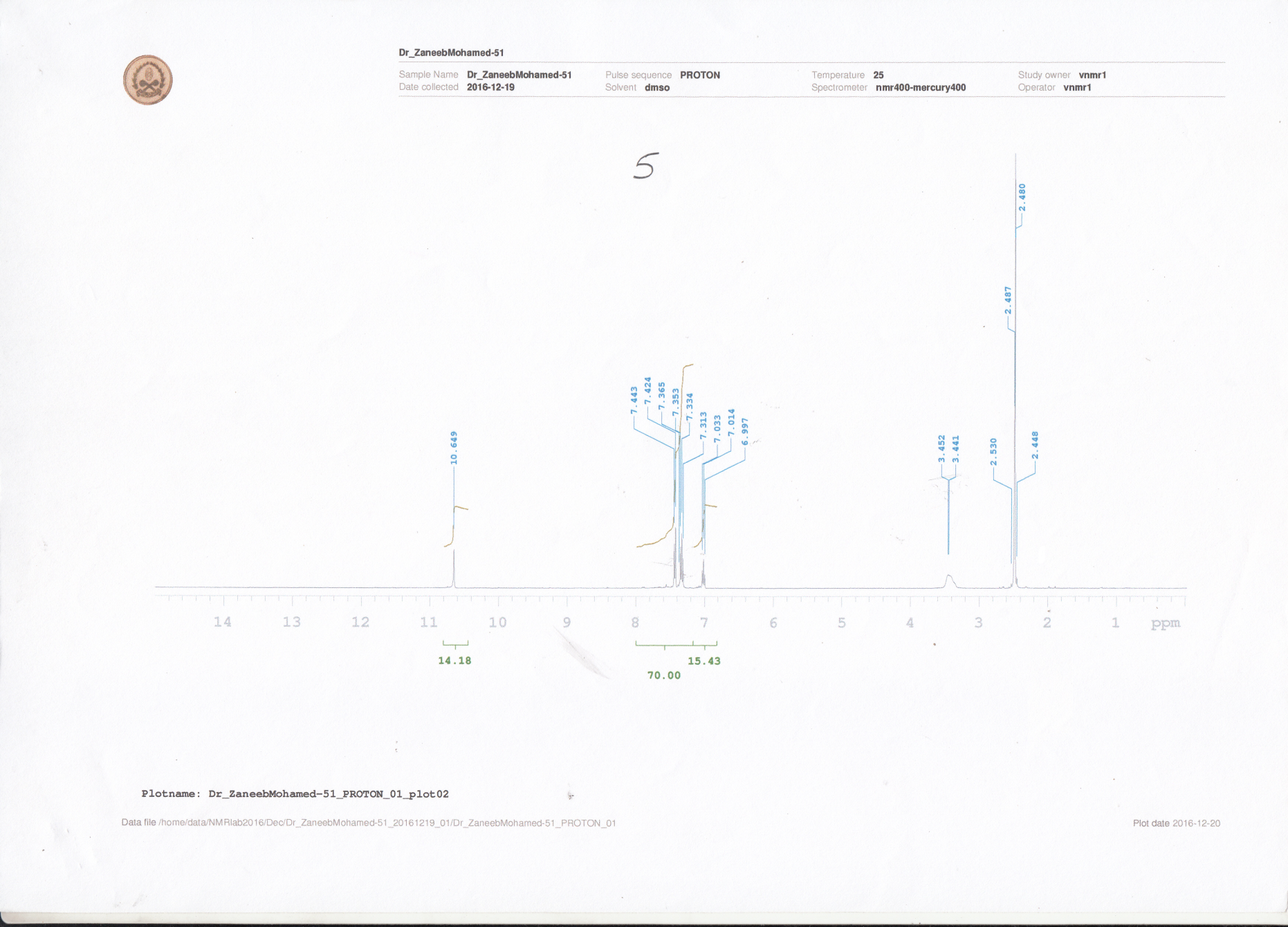


Compound **5** (^1^H NMR)


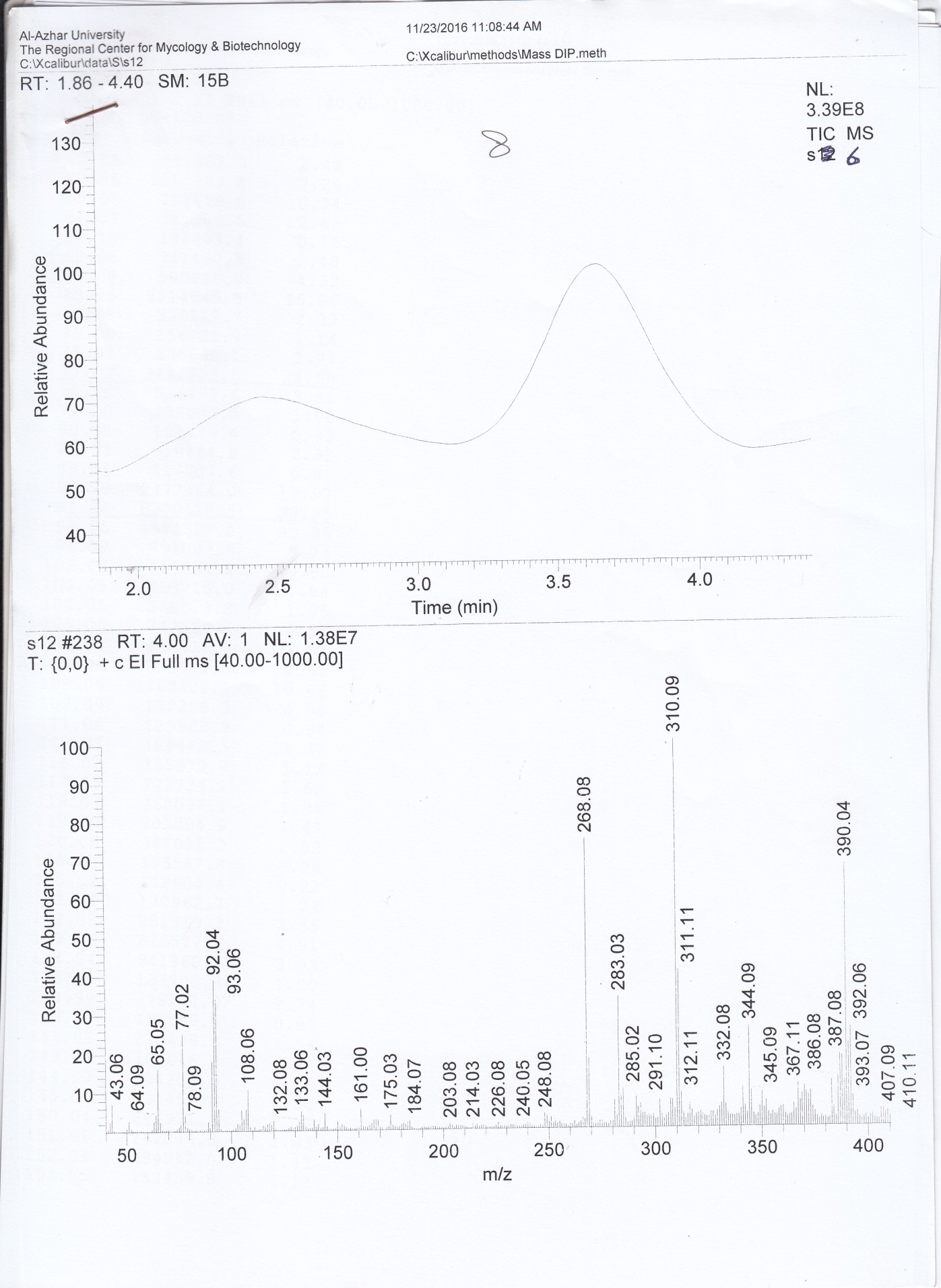


Compound **8** (Mass)


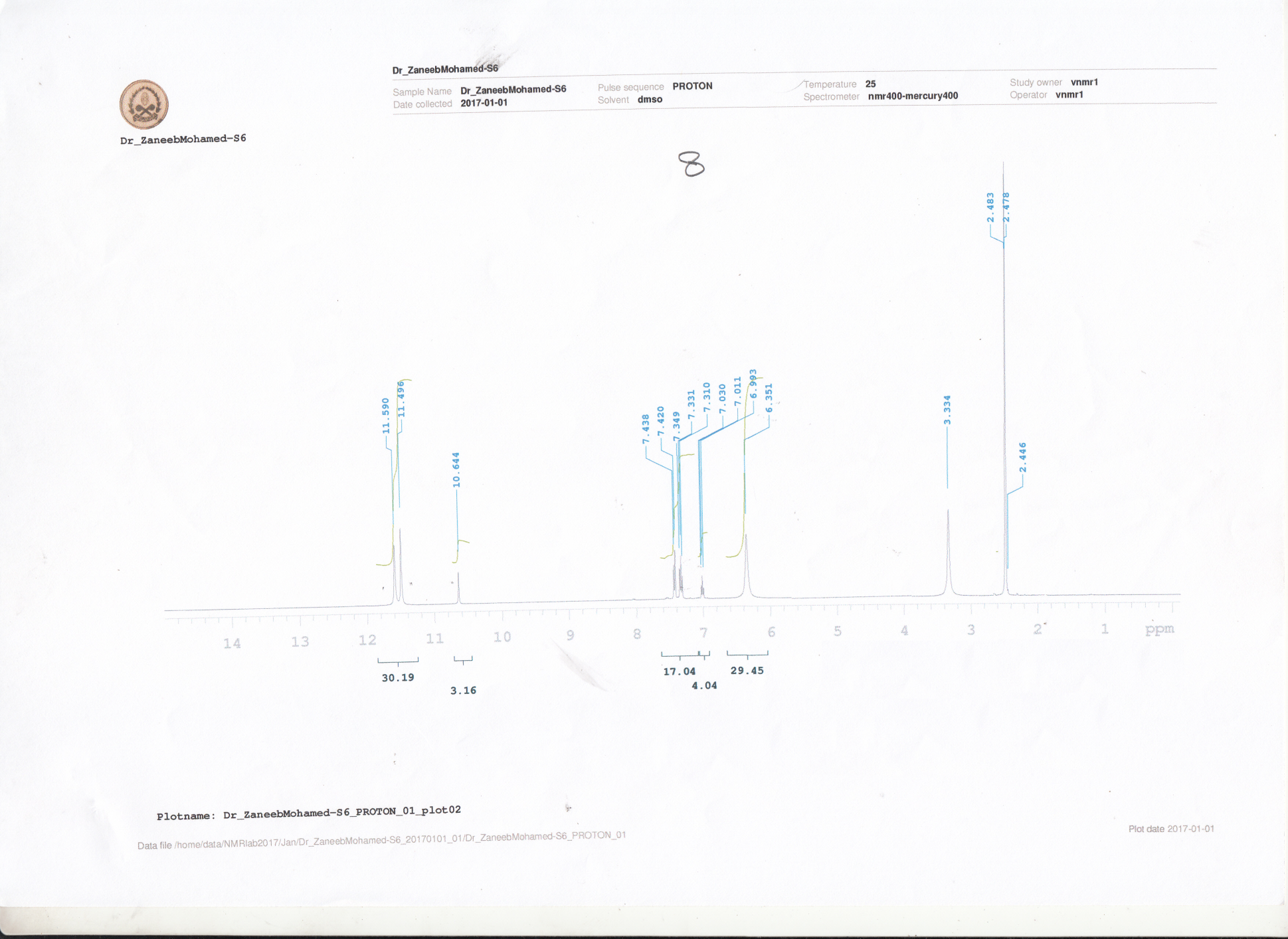


Compound **8** (^1^H NMR)


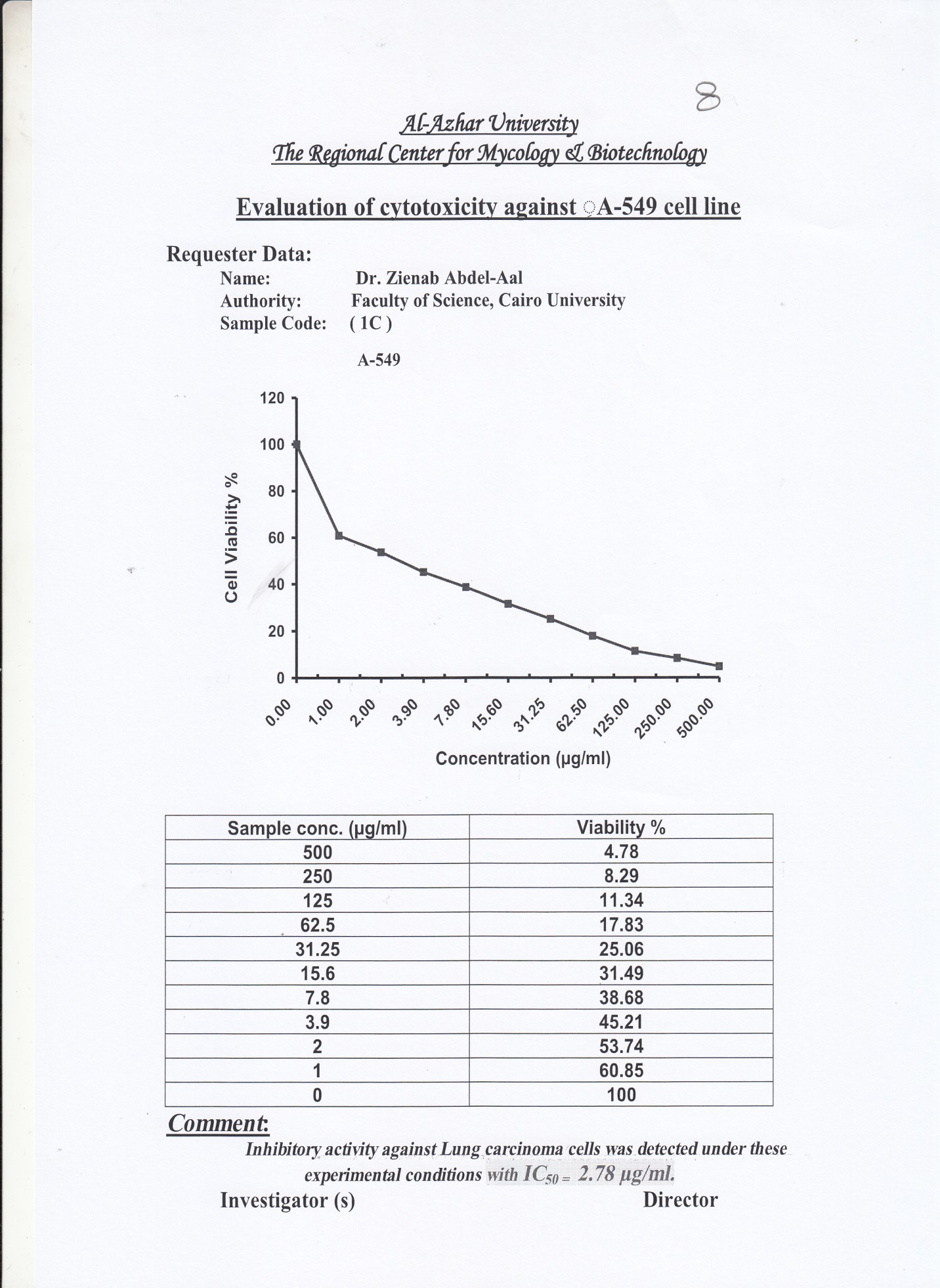


Compound **8** (Cytotoxic activity against A-549)


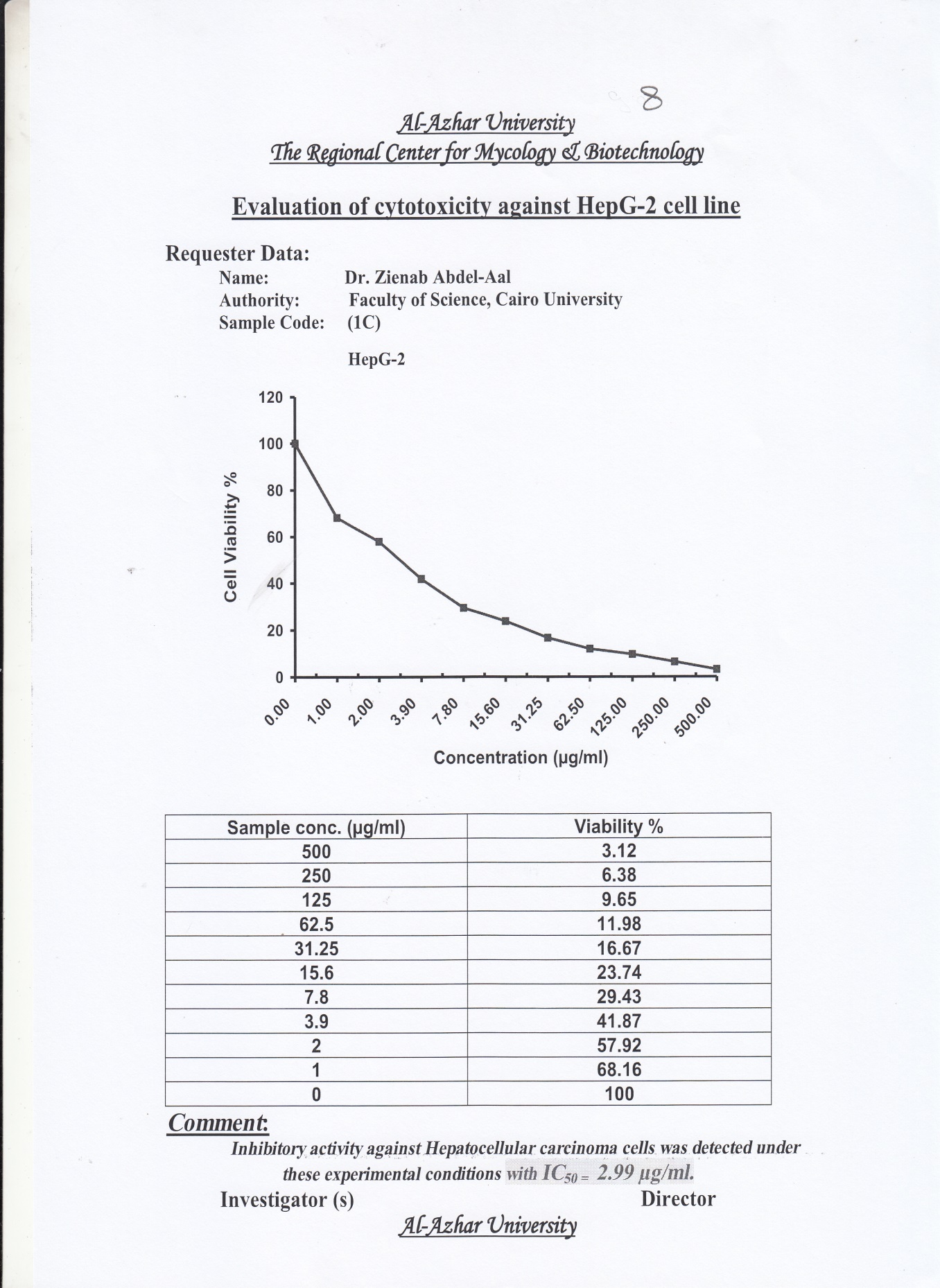


Compound **8** (Cytotoxic activity against HepG-2)


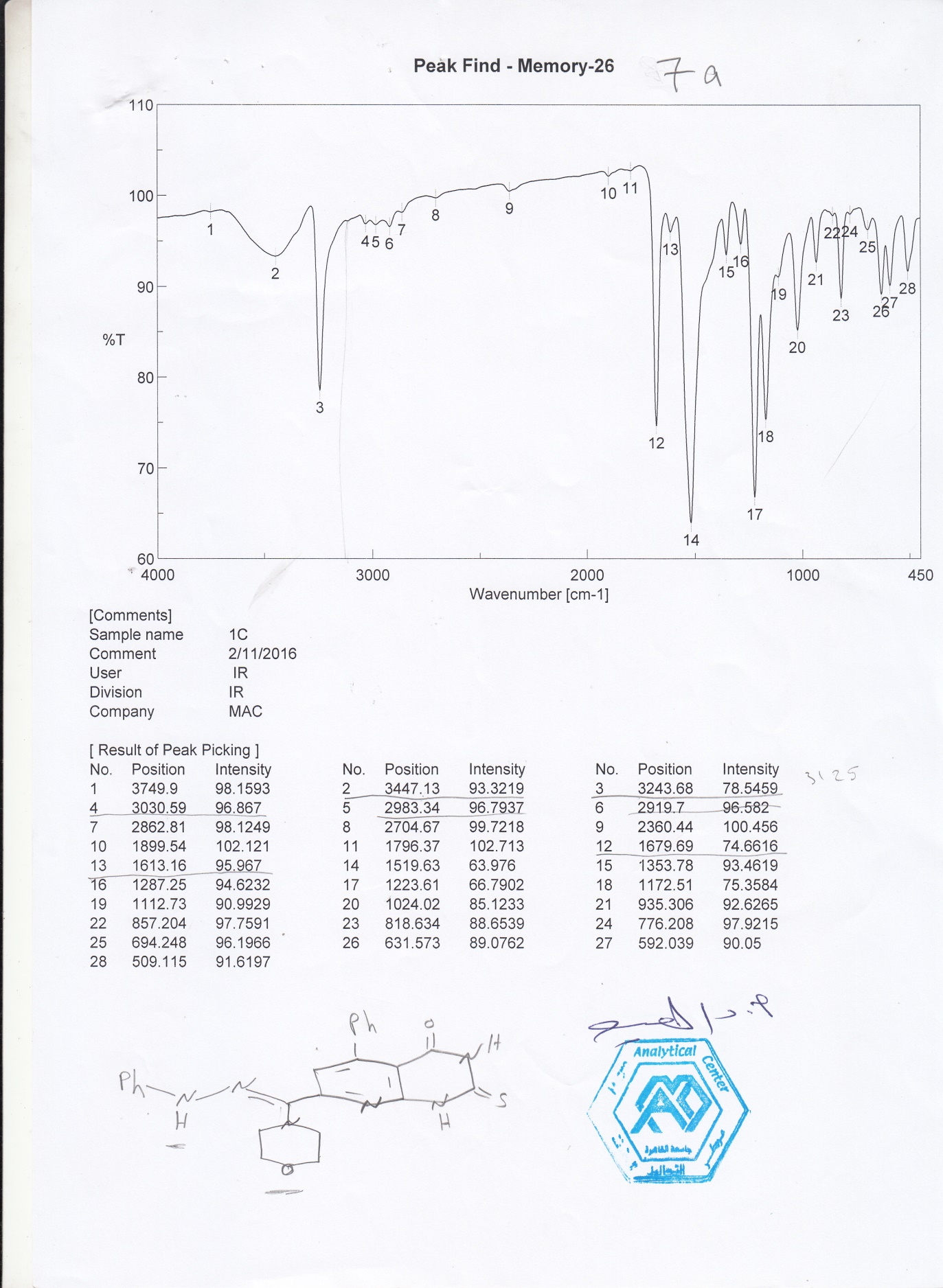


Compound **7a** (IR)


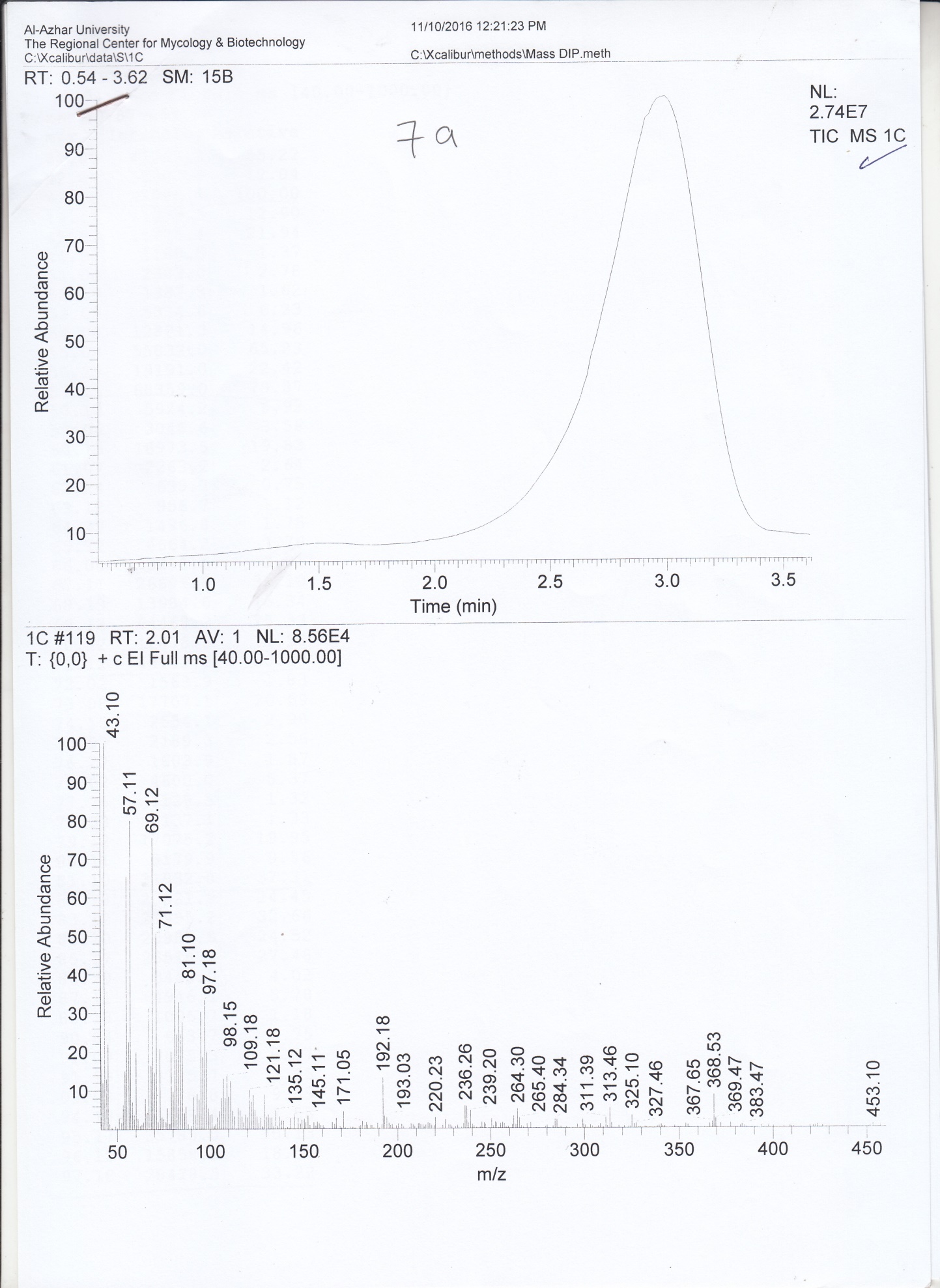


Compound **7a** (mass)


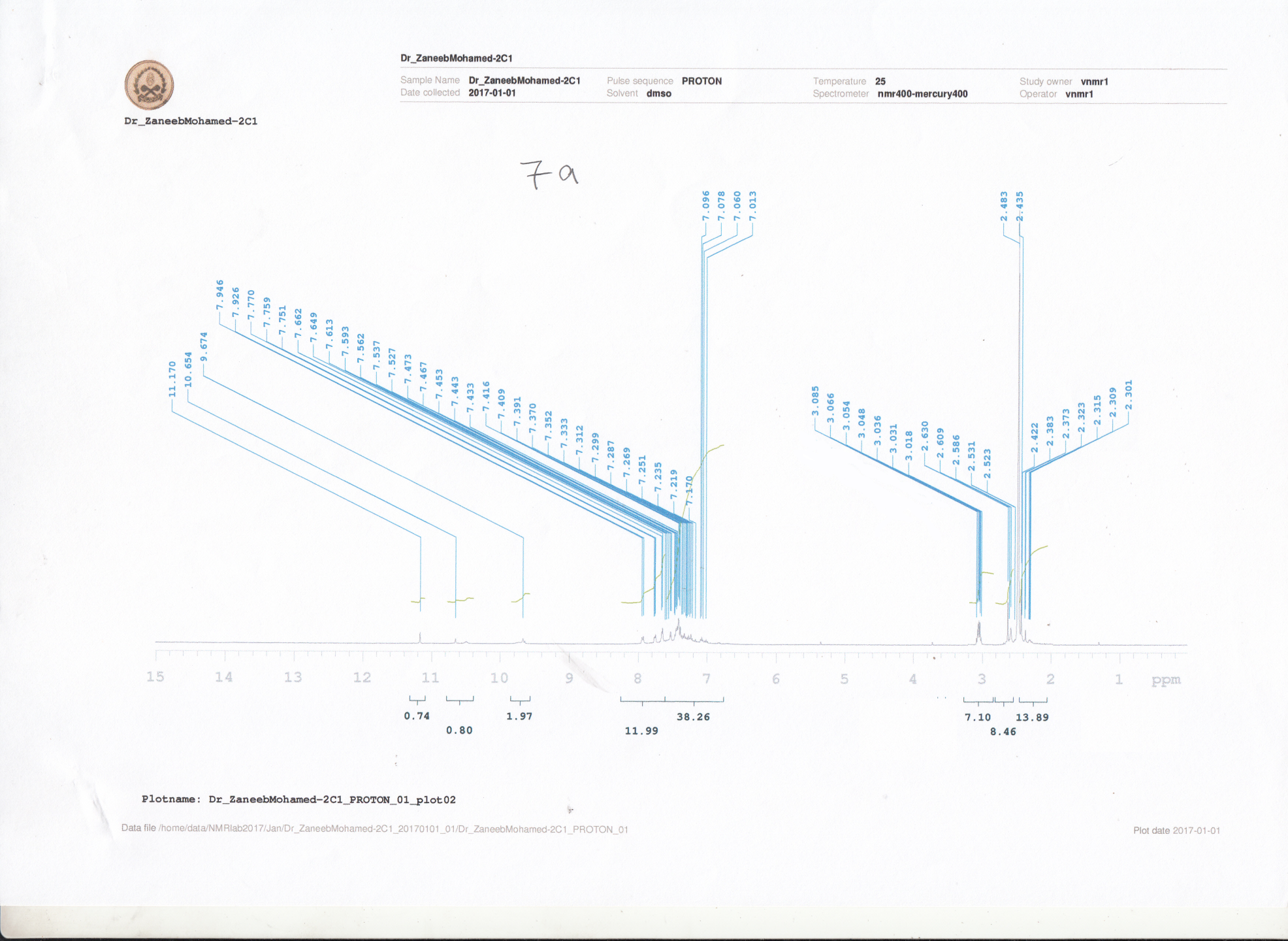


Compound **7a** (^1^H NMR)


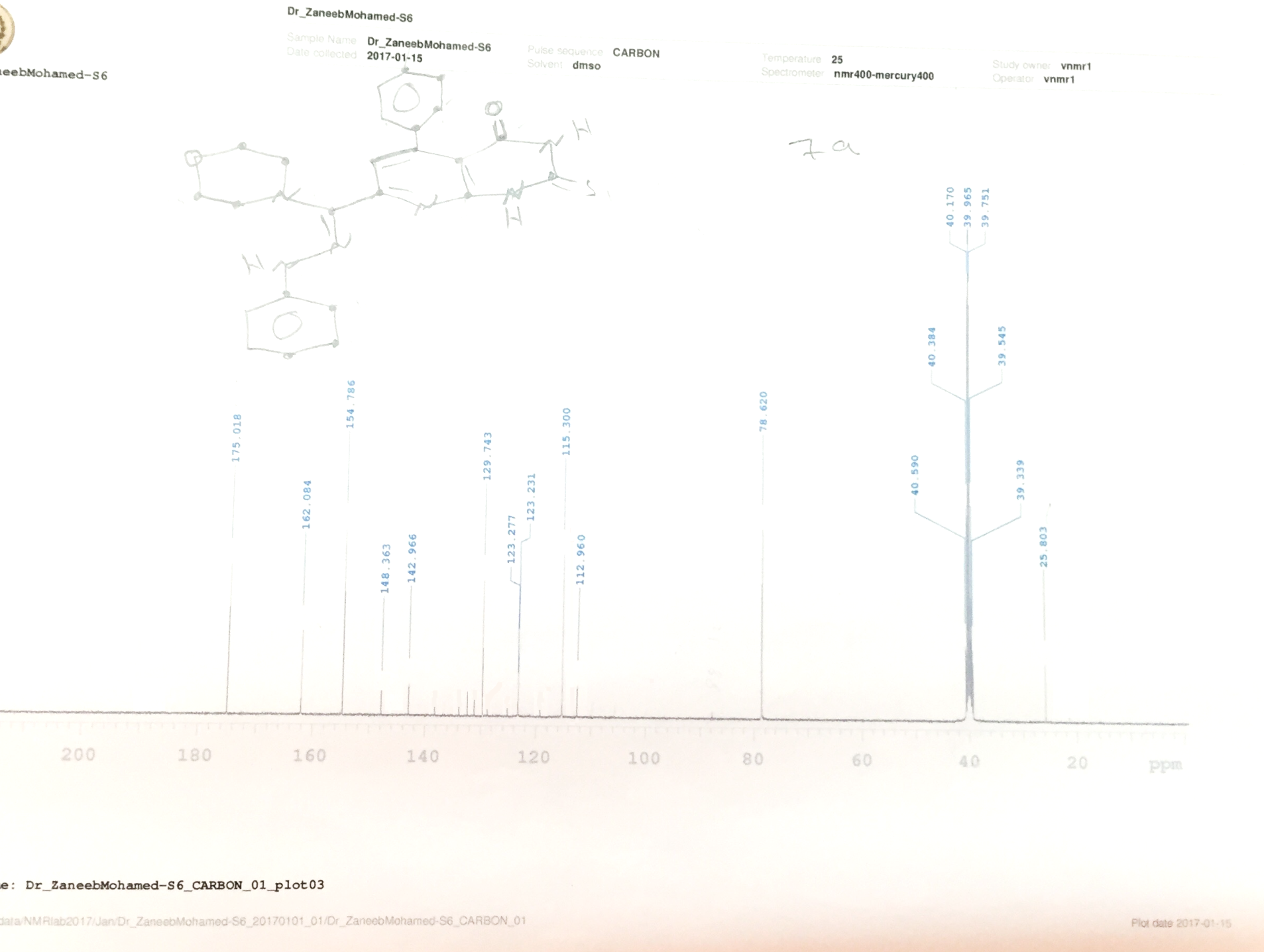


Compound **7a** (^13^C- NMR)


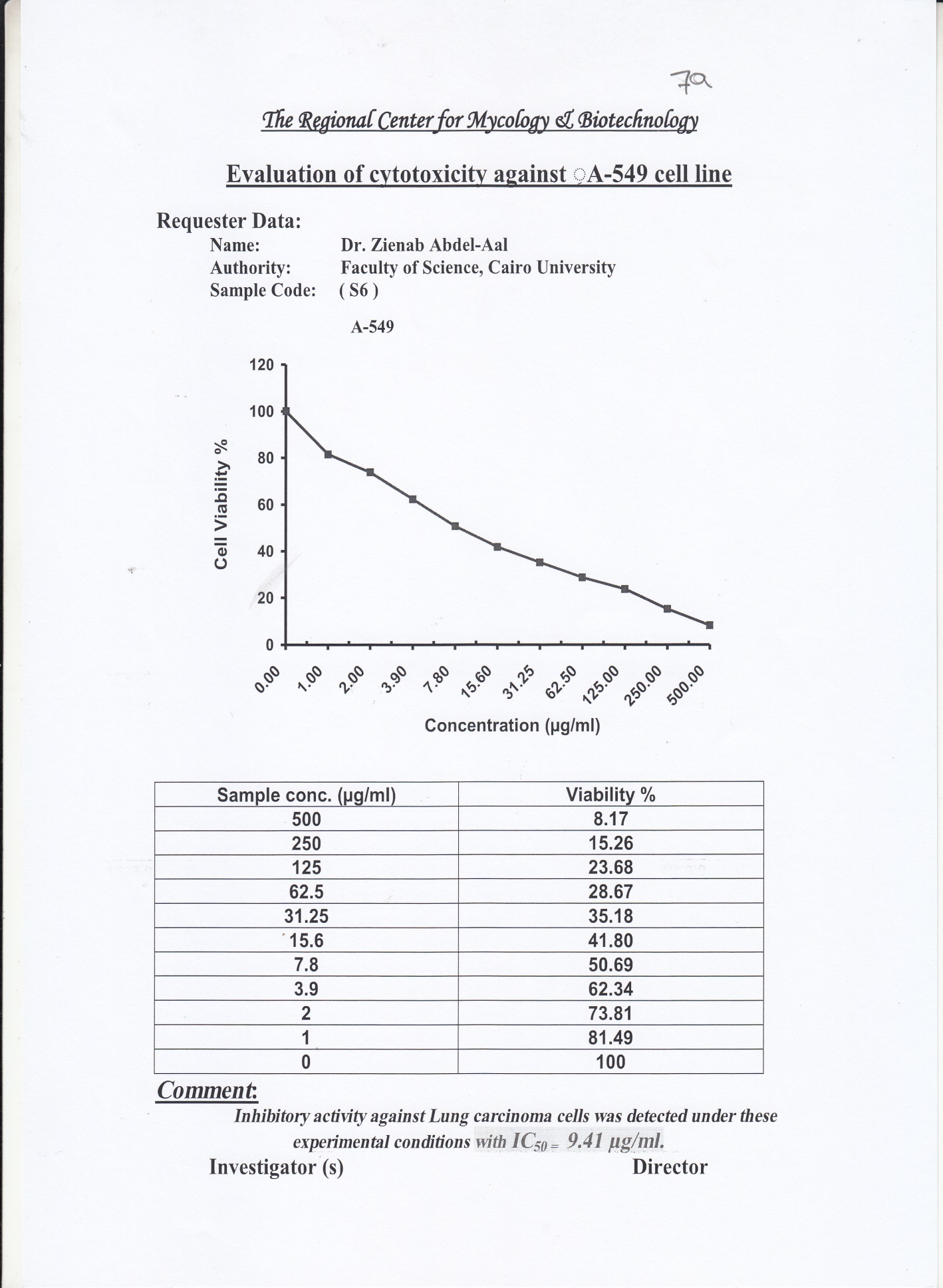


Compound **7a** (Cytotoxic activity against A-549)


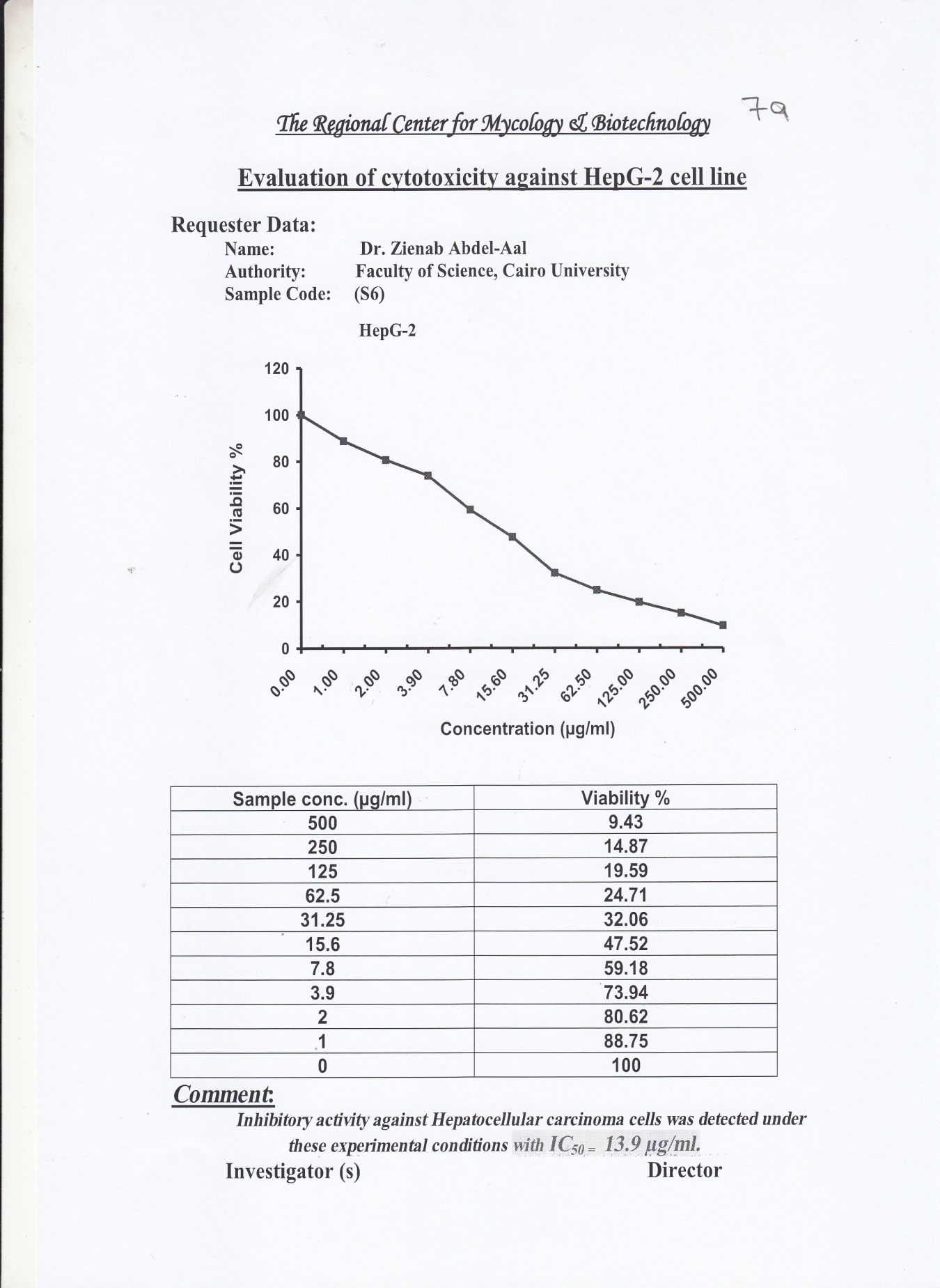


Compound **7a** (Cytotoxic activity against HepG-2)


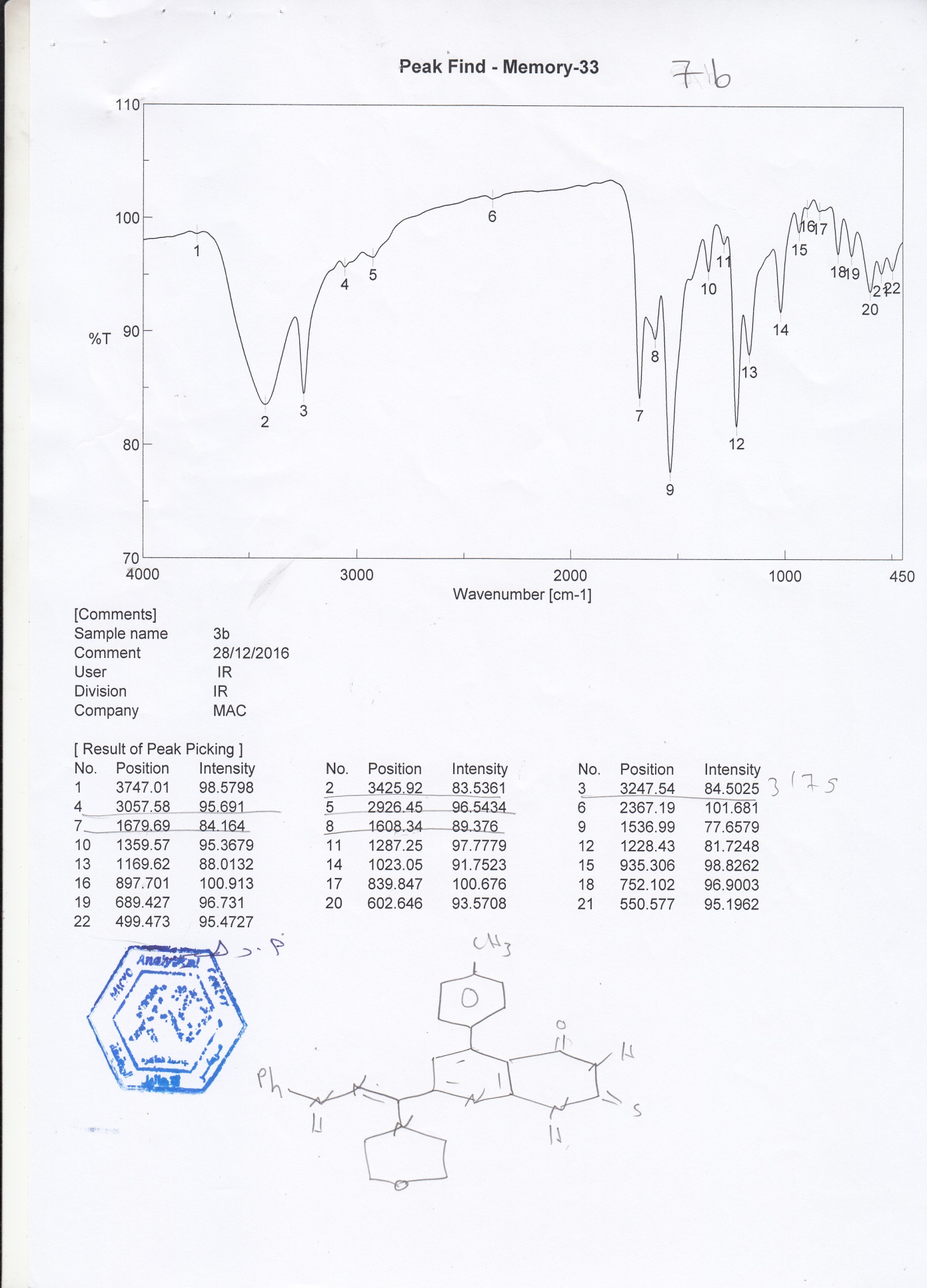


Compound **7b** (IR)


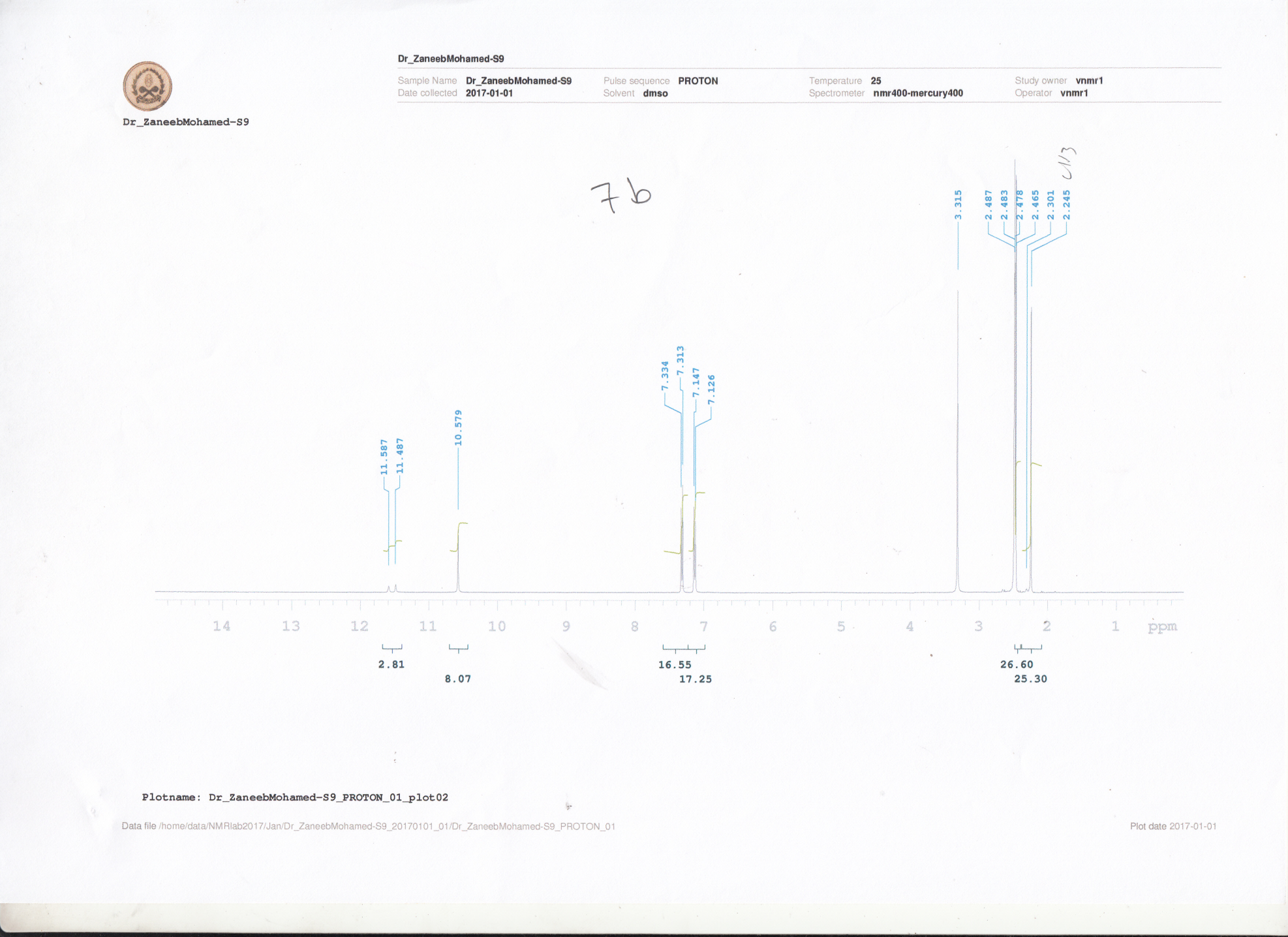


Compound **7b** (^1^H NMR)


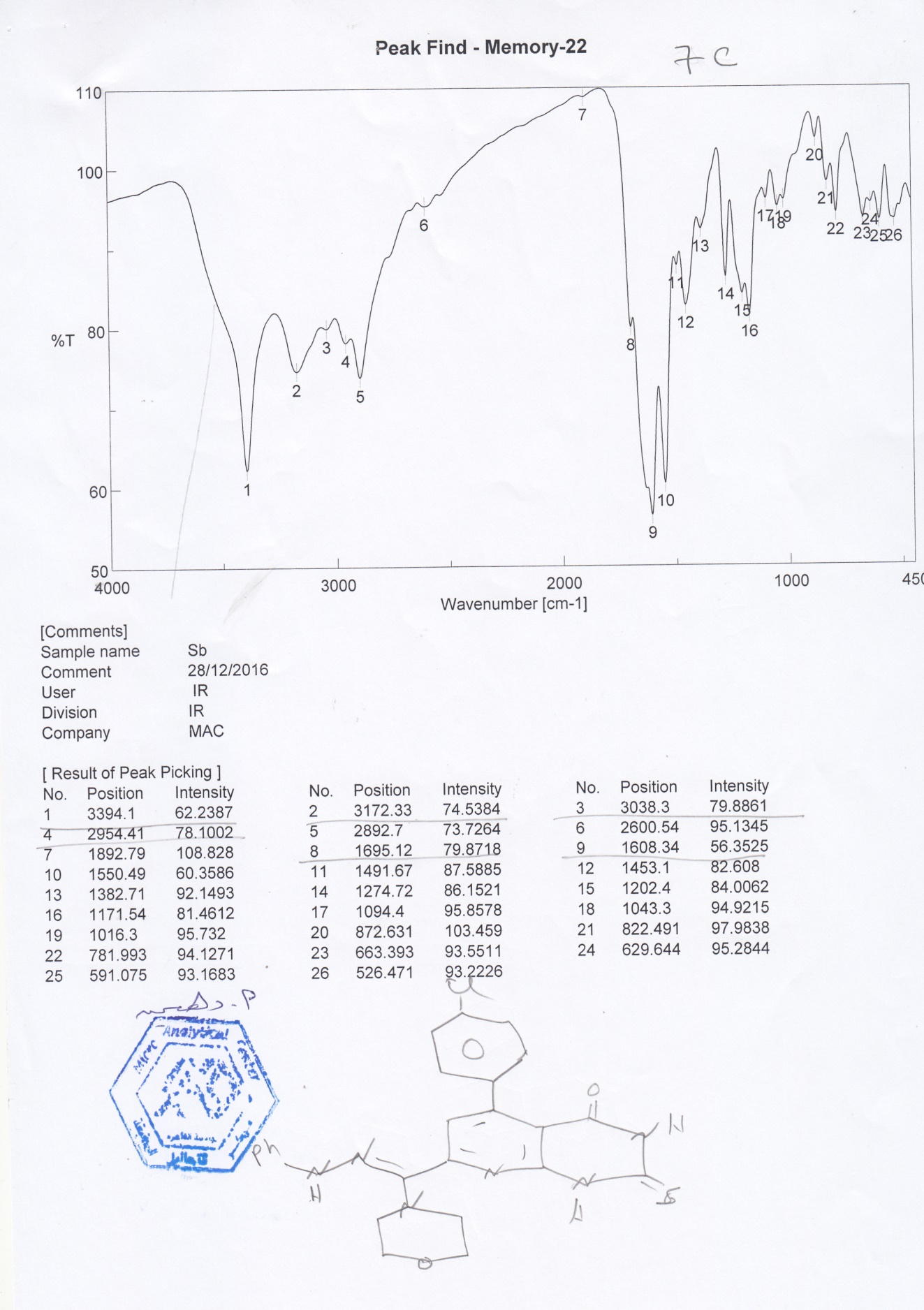


Compound **7c** (IR)


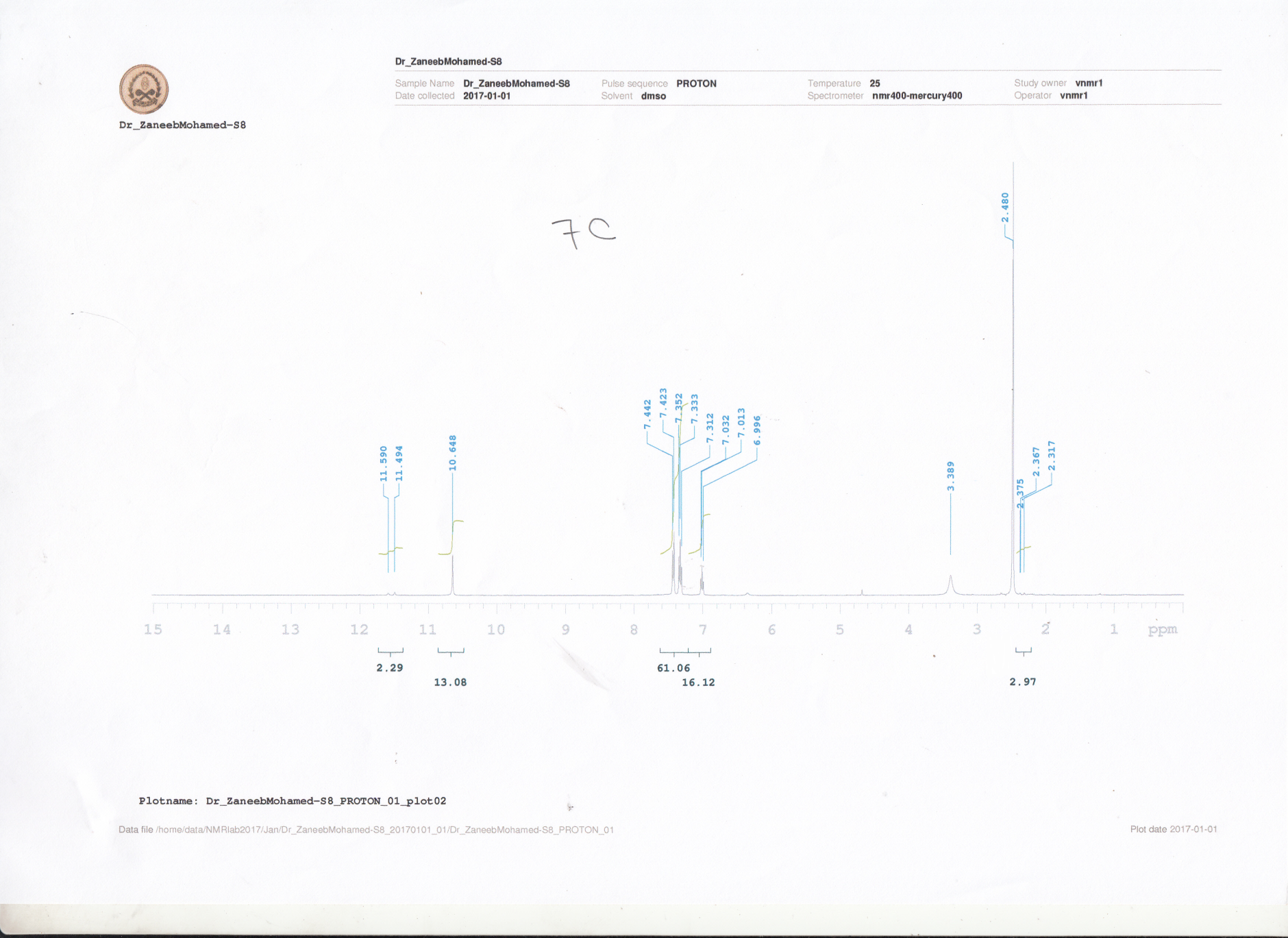


Compound **7c** (^1^H NMR)


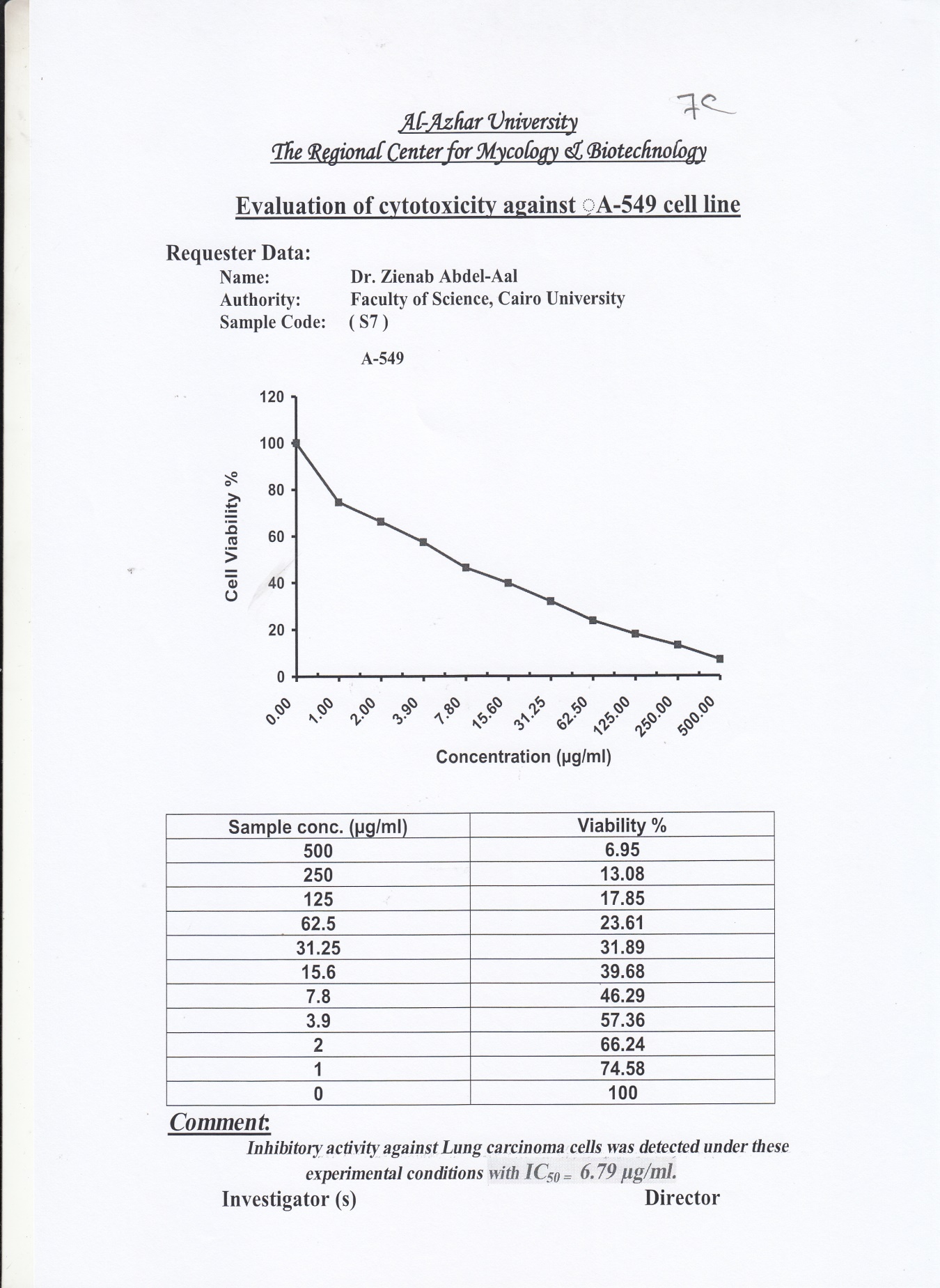


Compound **7c** (Cytotoxic activity against A-549)


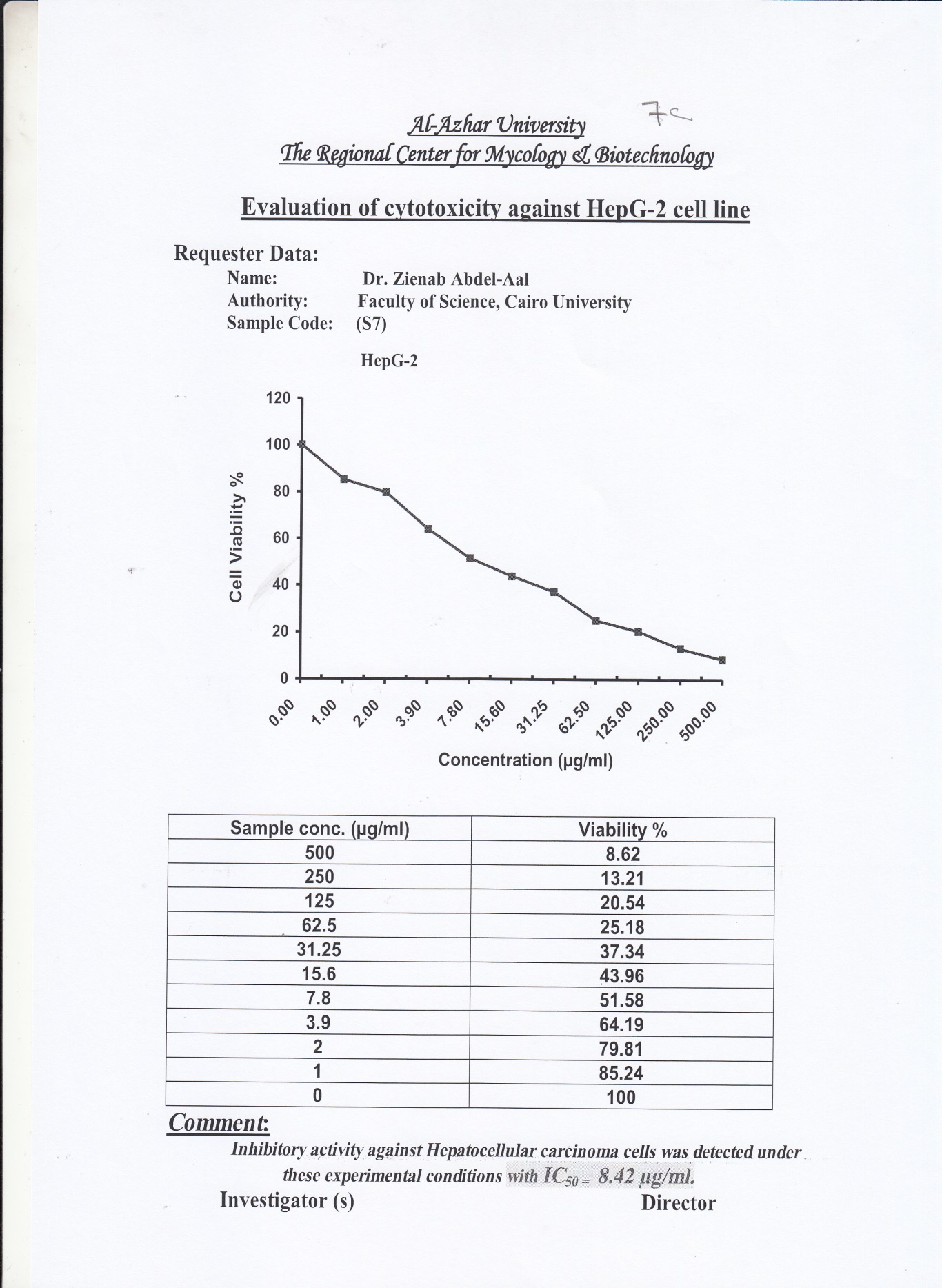


Compound **7c** (Cytotoxic activity against HepG-2)


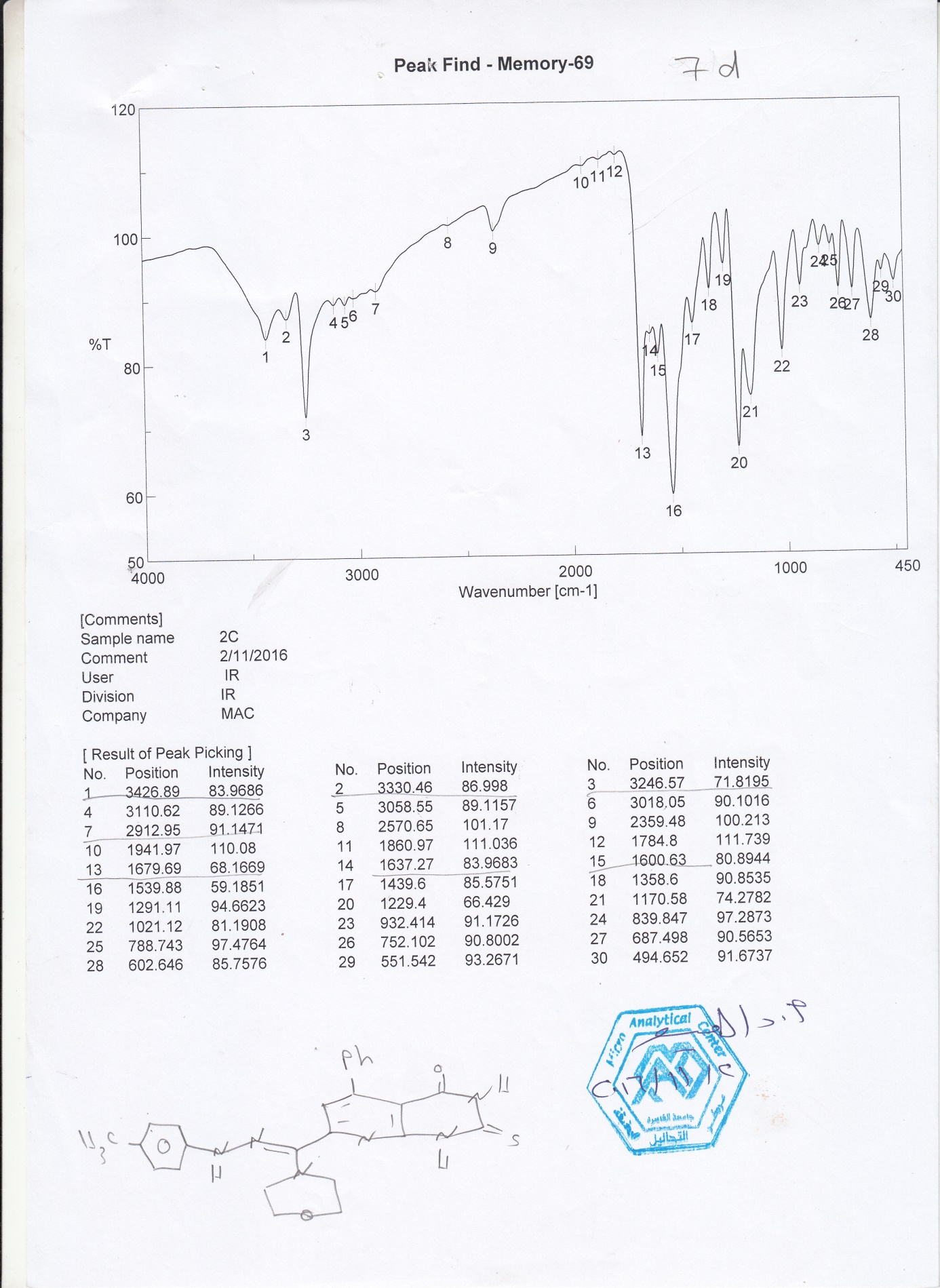


Compound **7d** (IR)


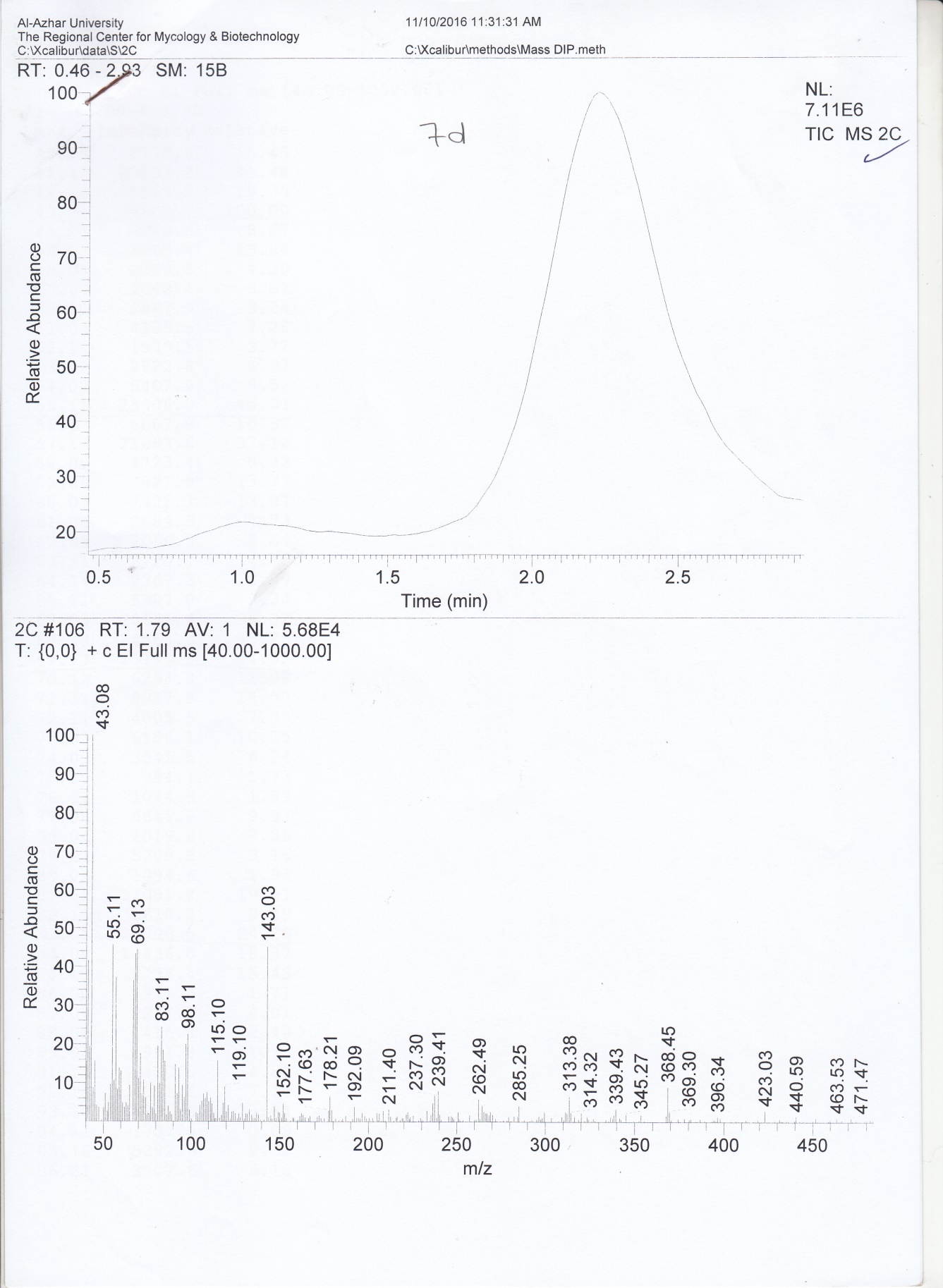


Compound **7d** (mass)


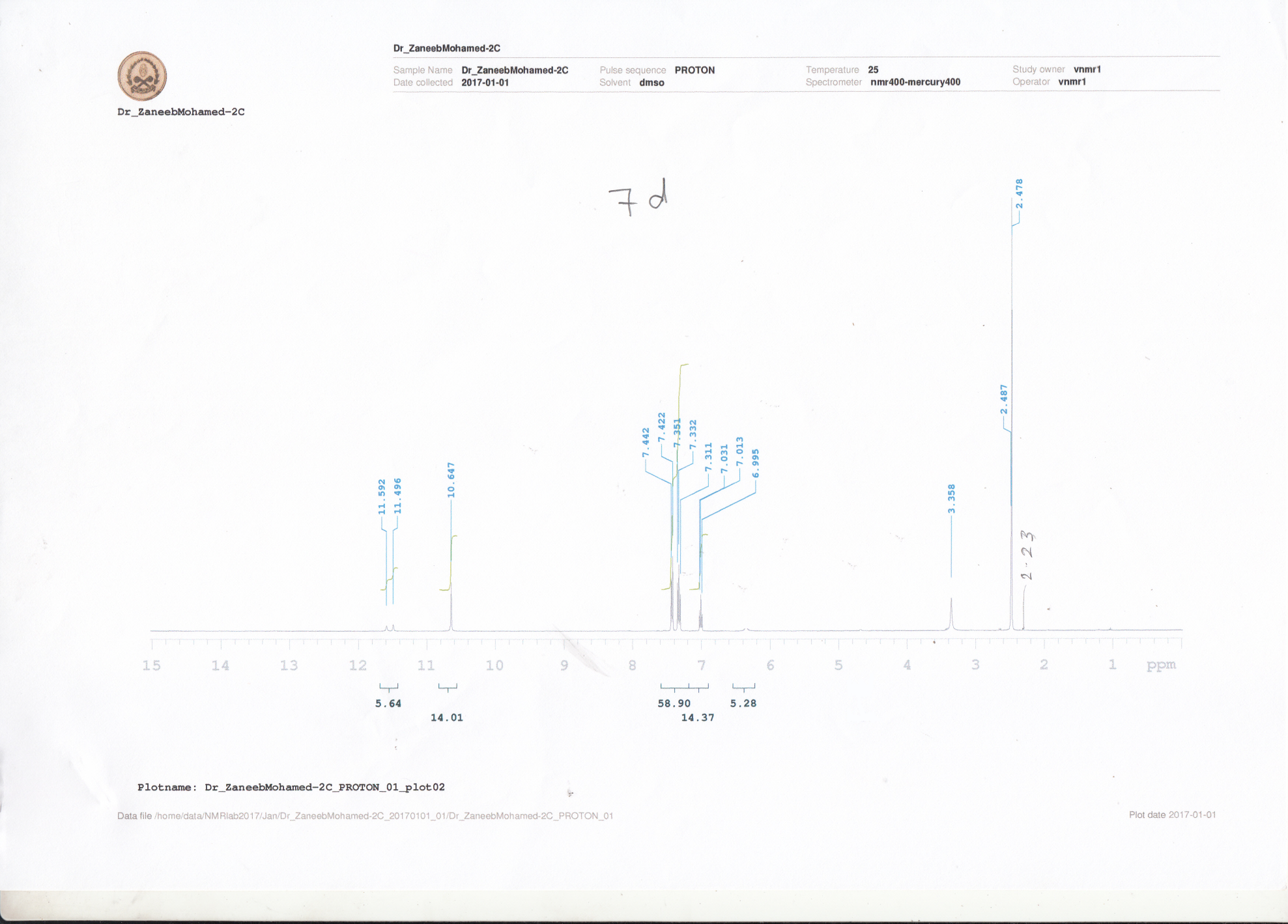


Compound **7d** (^1^HNMR)


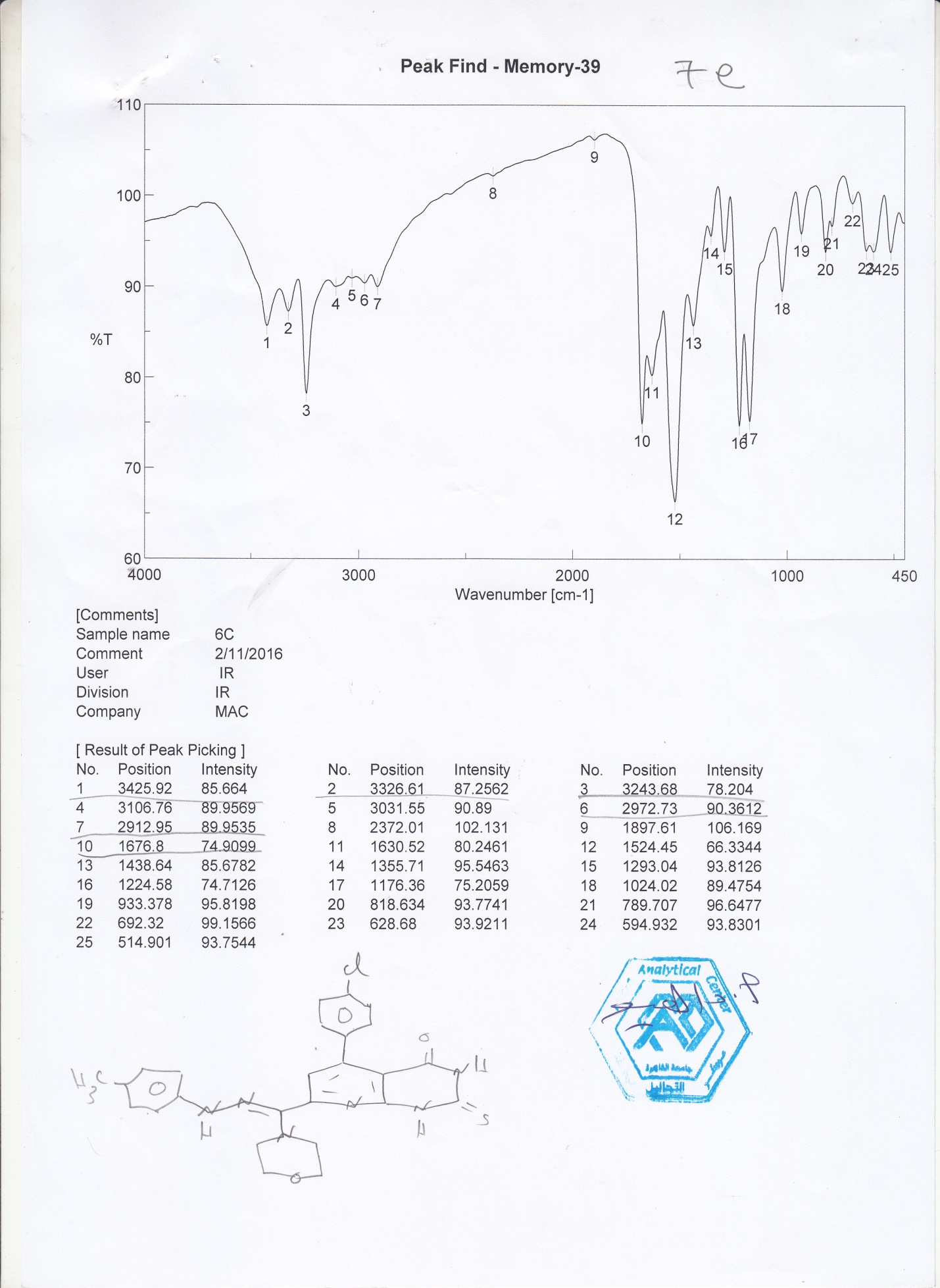


Compound **7e** (IR)


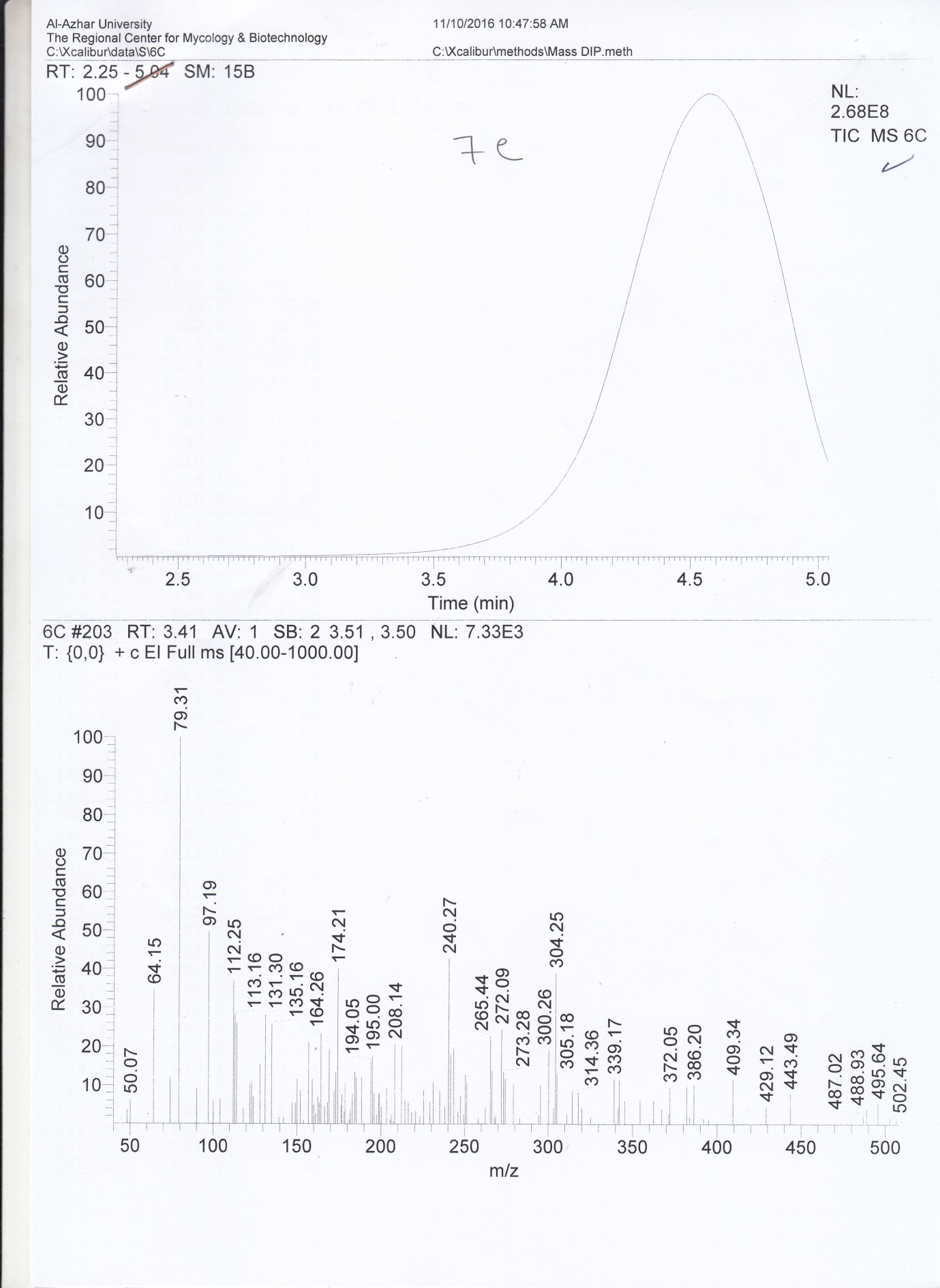


Compound **7e** (Mass)


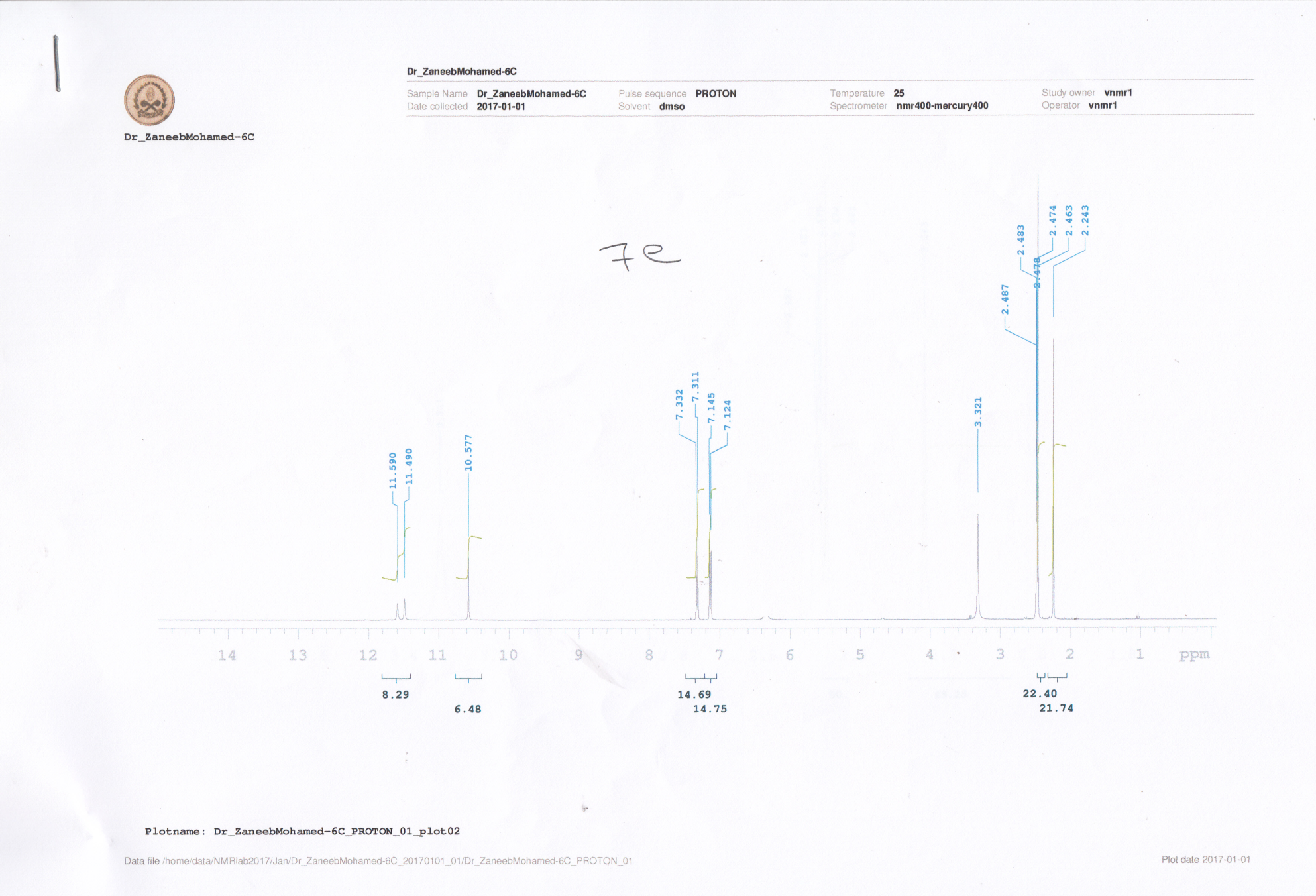


Compound **7e** (^1^H NMR)


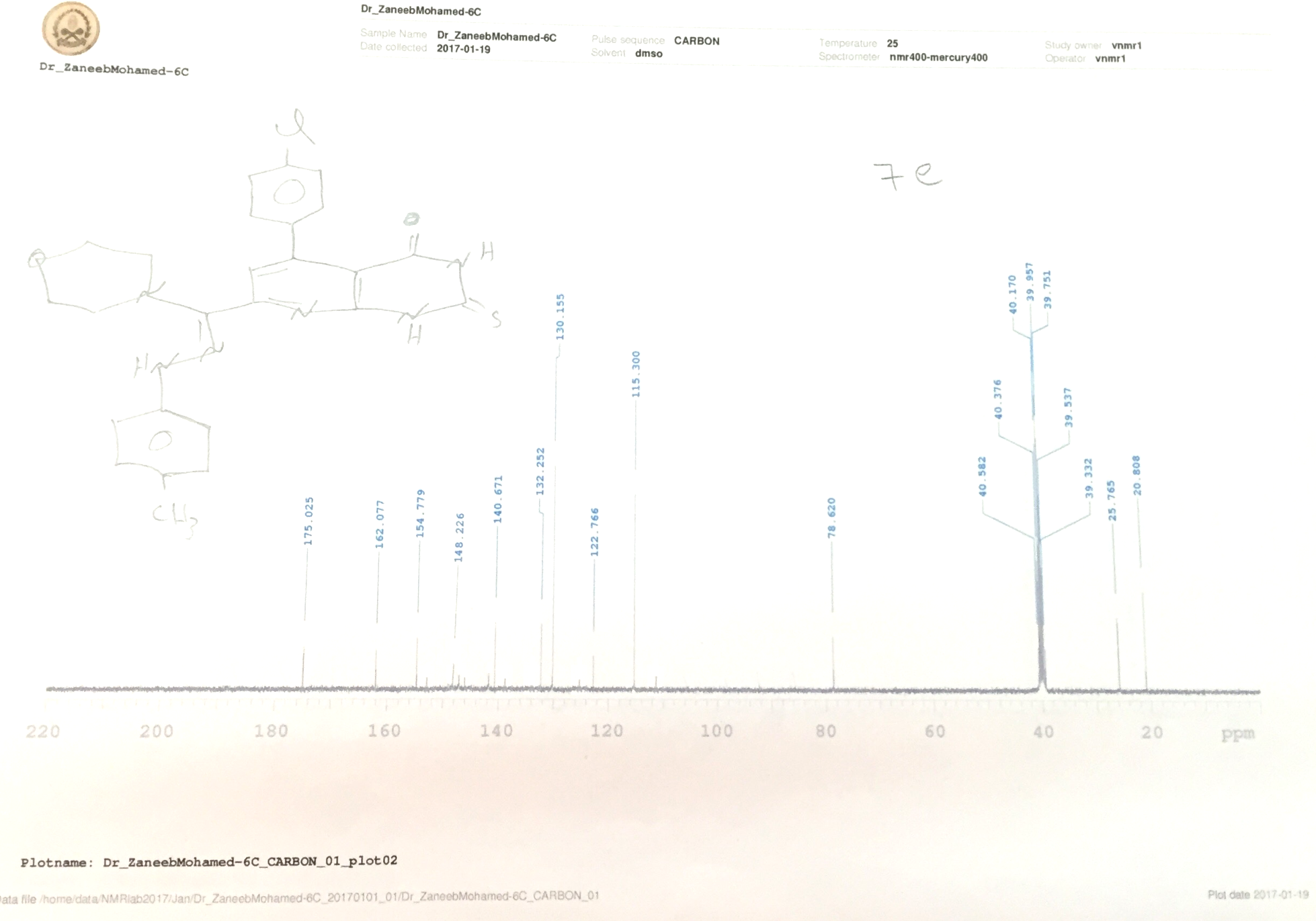


Compound **7e** (^13^C- NMR)


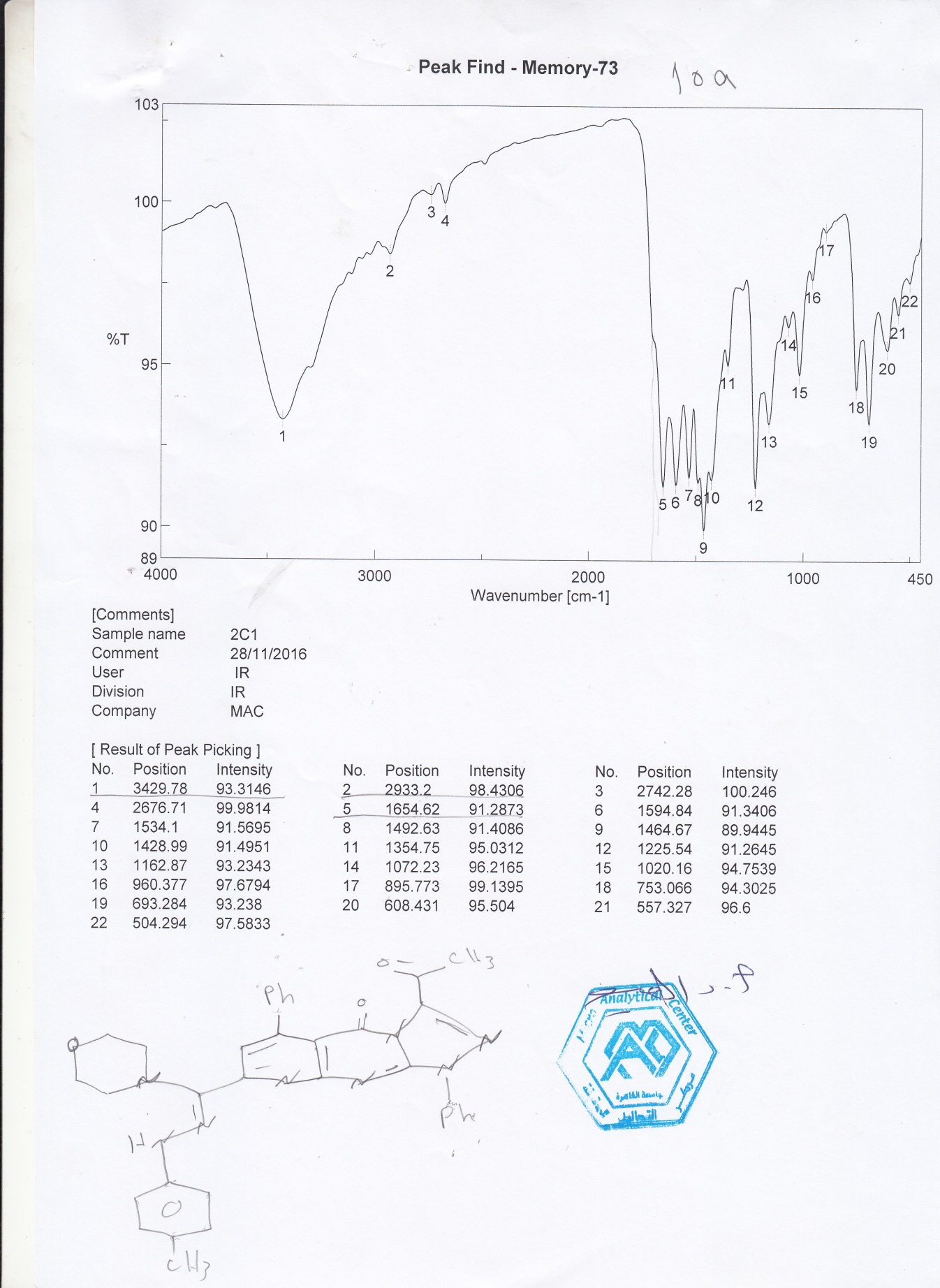


Compound **10a** (IR)


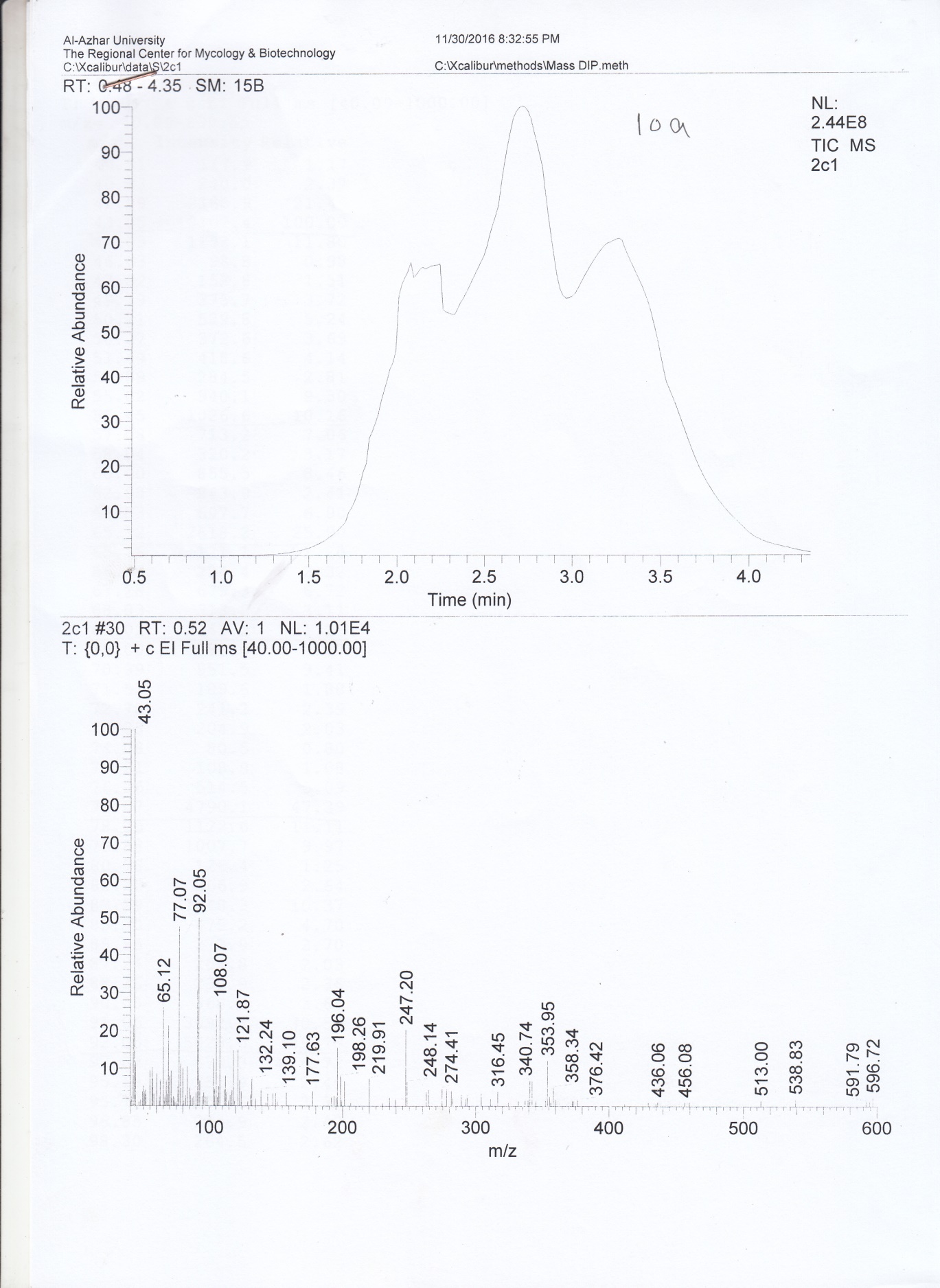


Compound **10a** (mass)


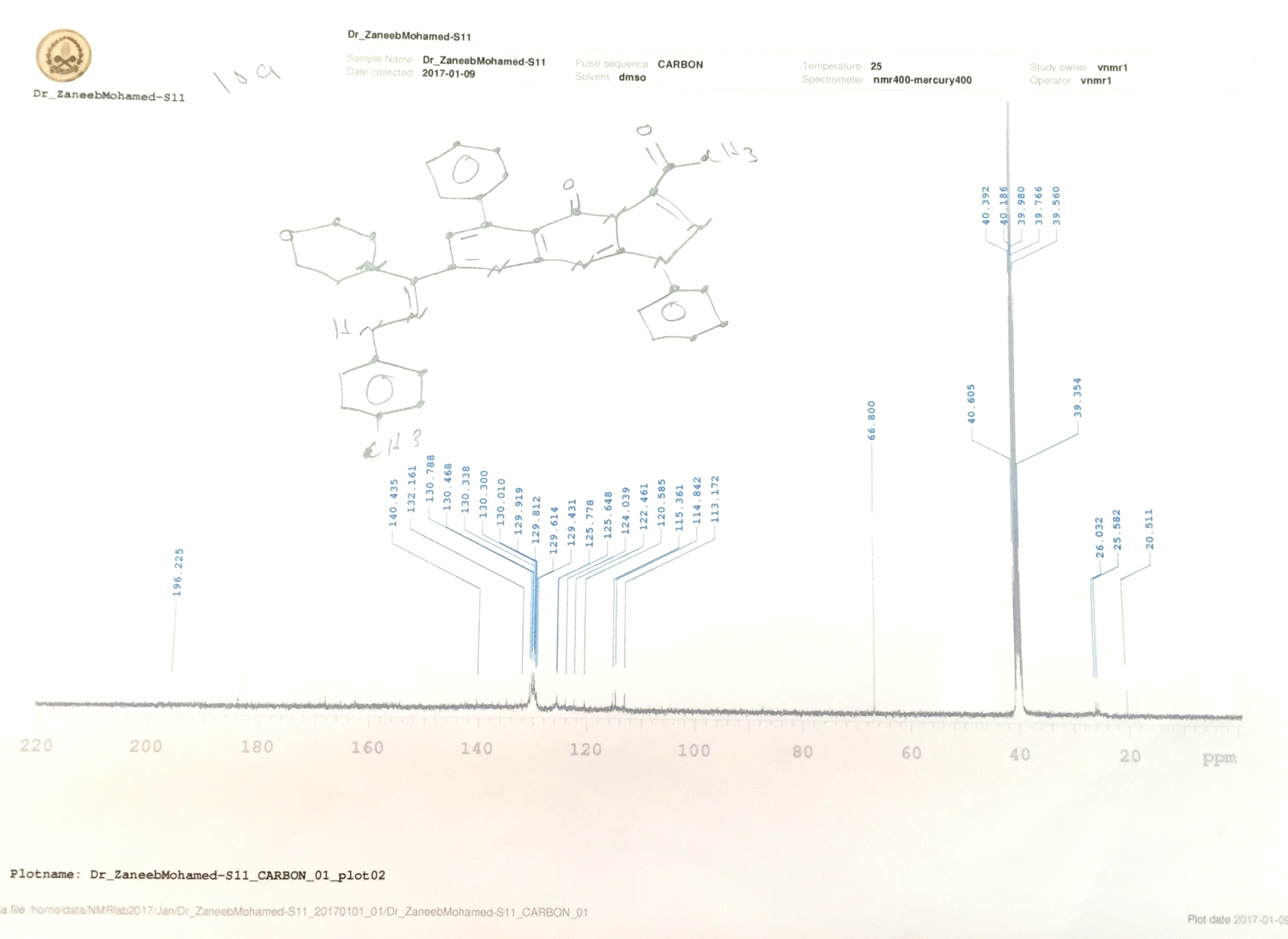


Compound **10a** (^13^C-NMR)


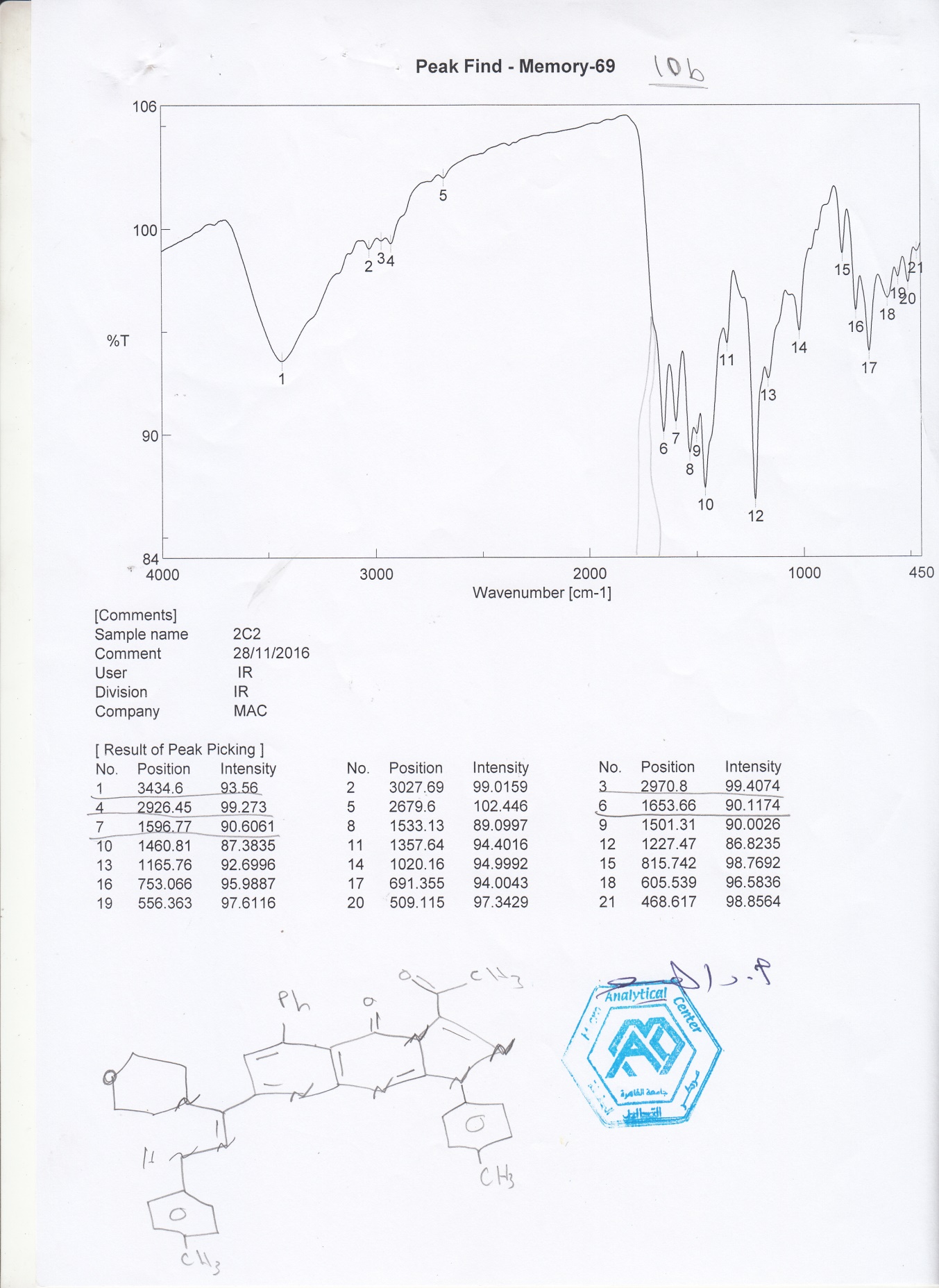


Compound **10b** ( IR)


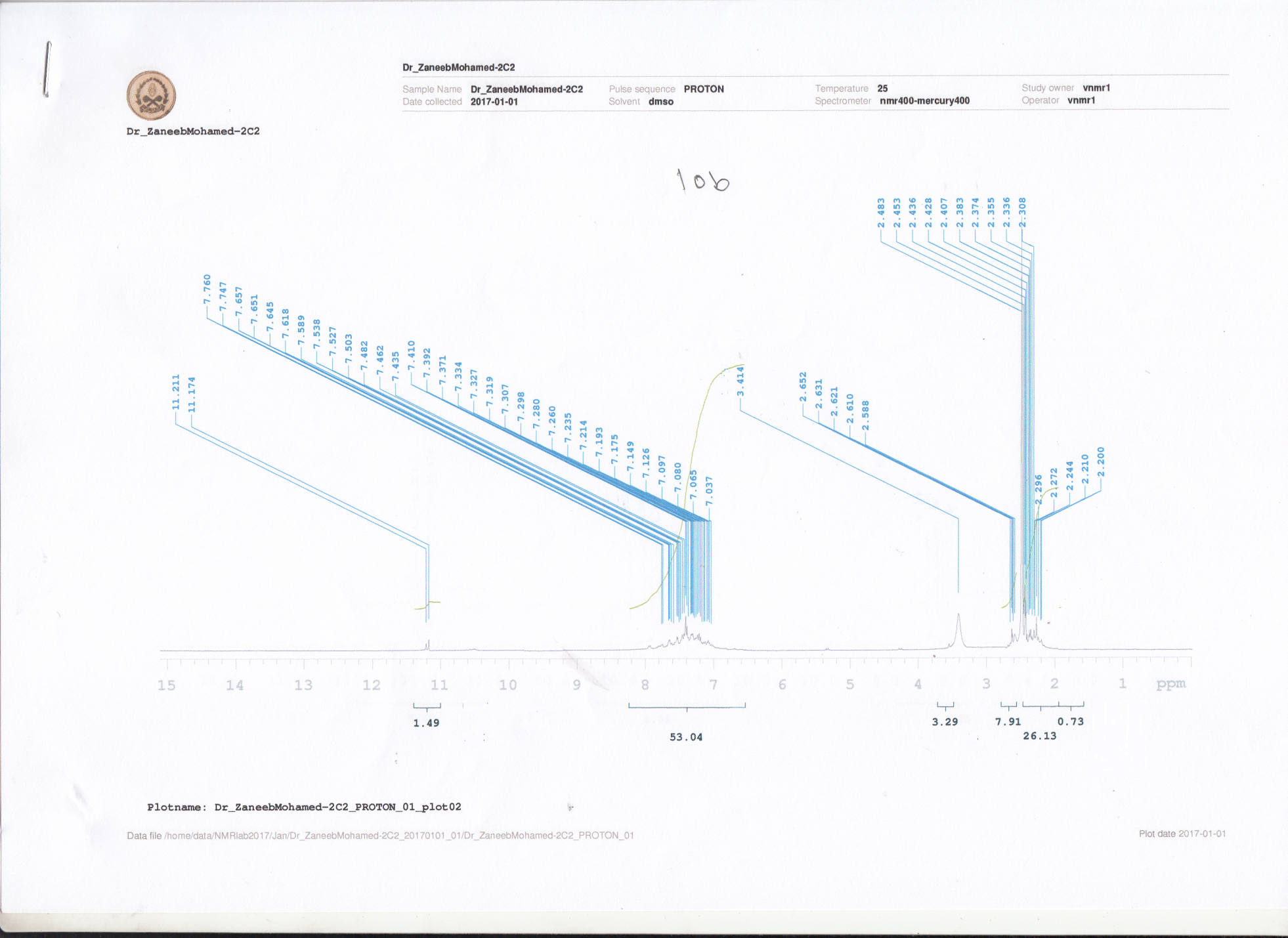


Compound **10b** (^1^H NMR)


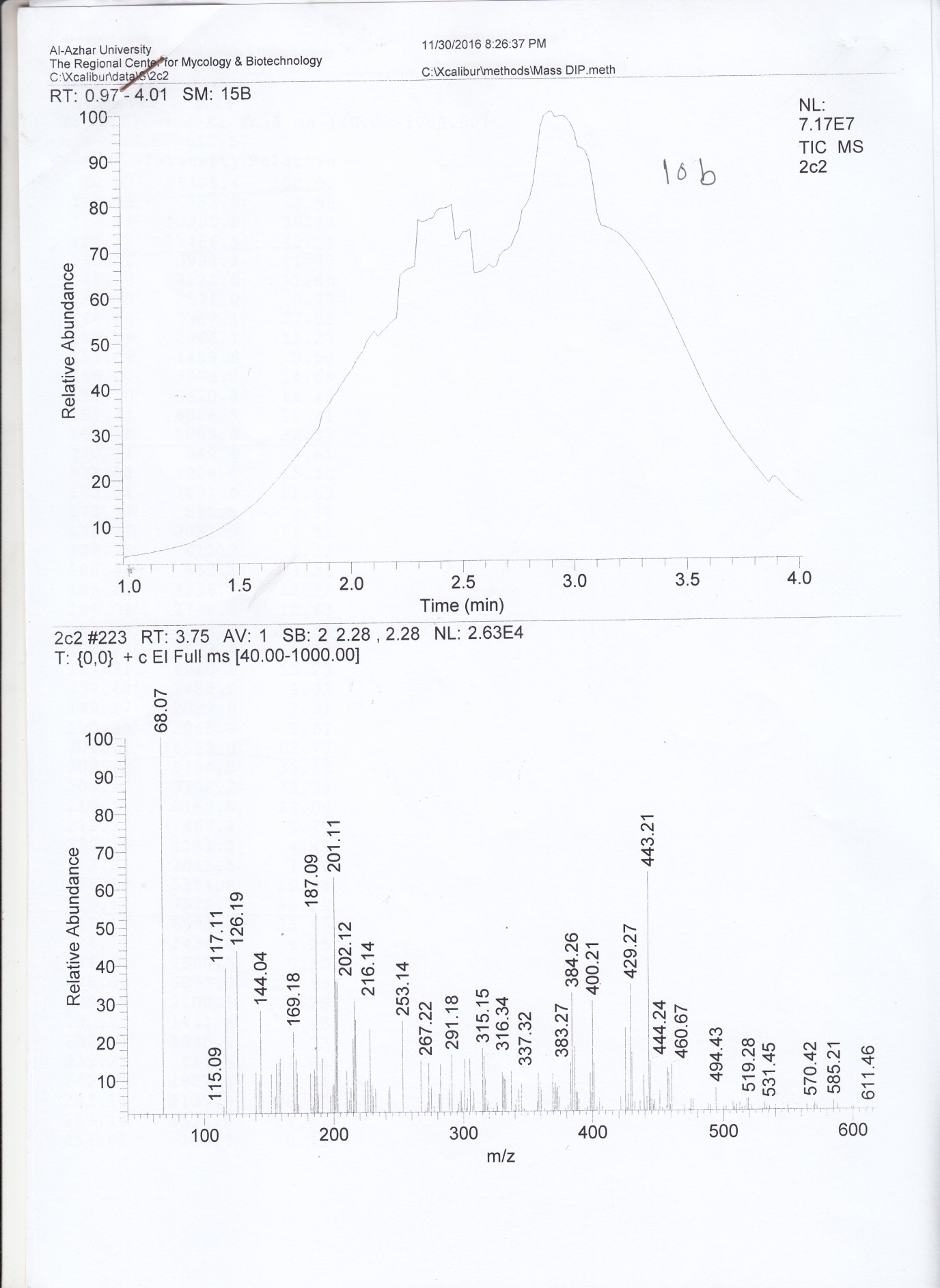


Compound **10b** (mass)


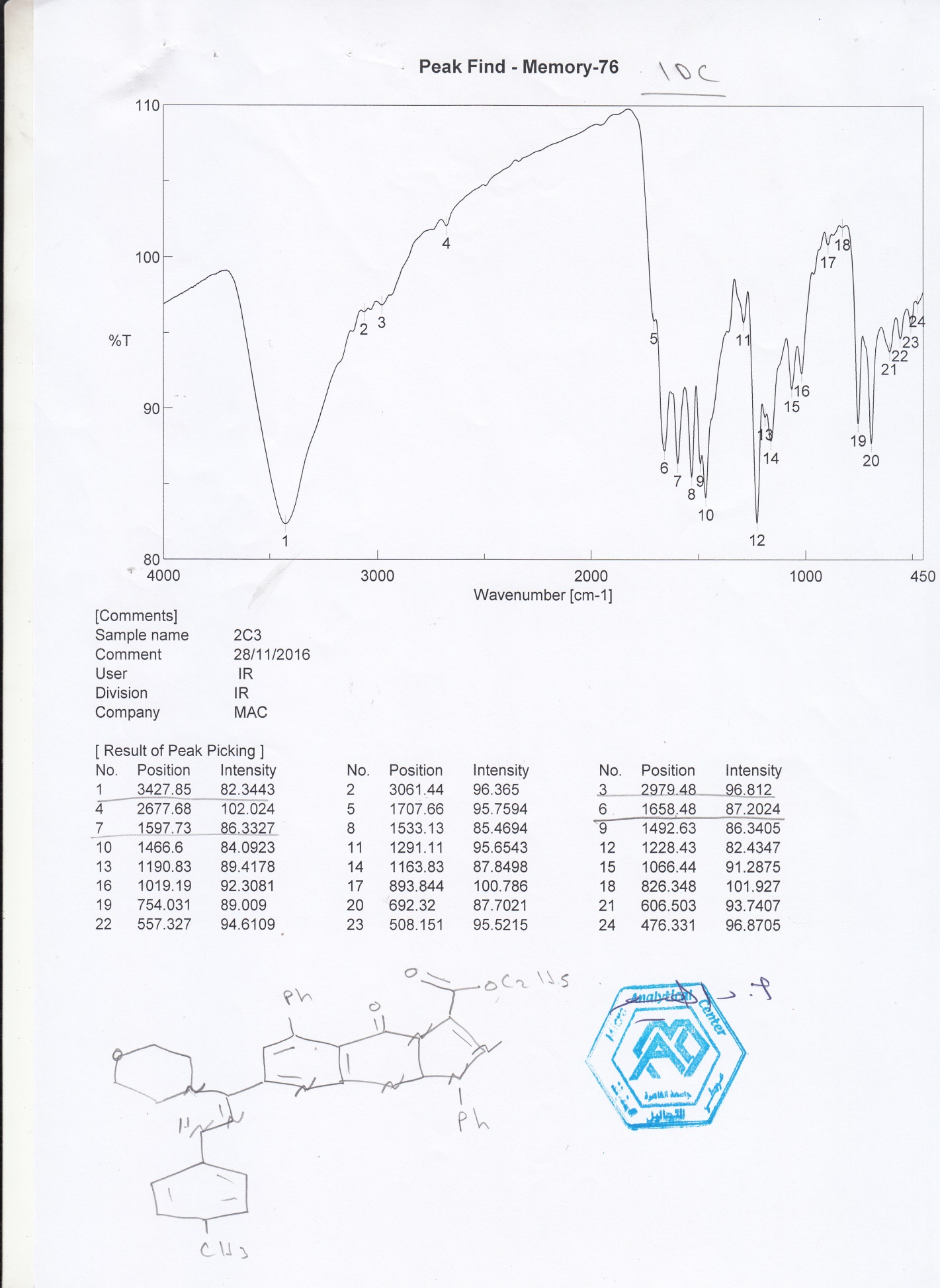


Compound **10c** (IR)


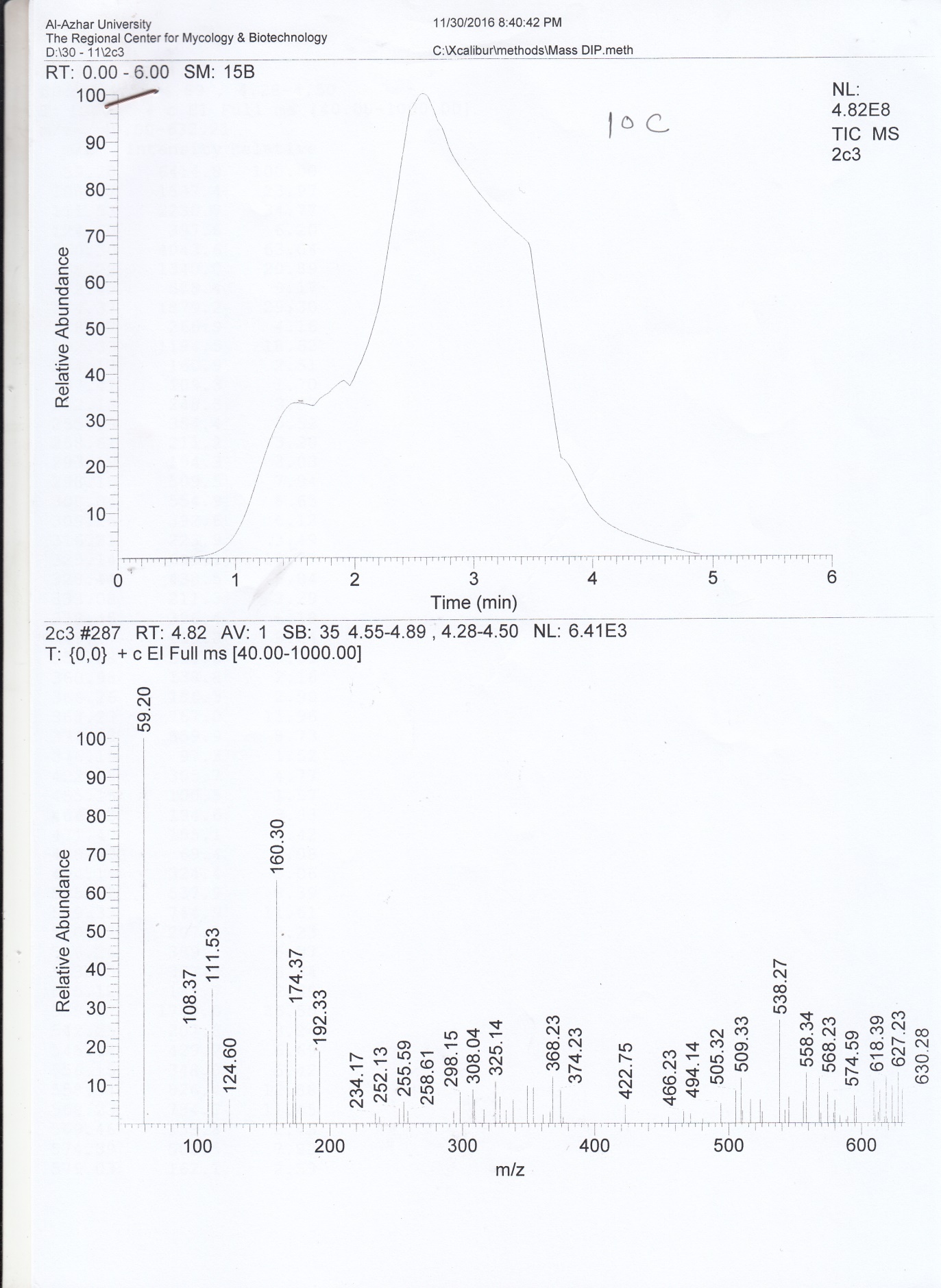


Compound **10c** (Mass)


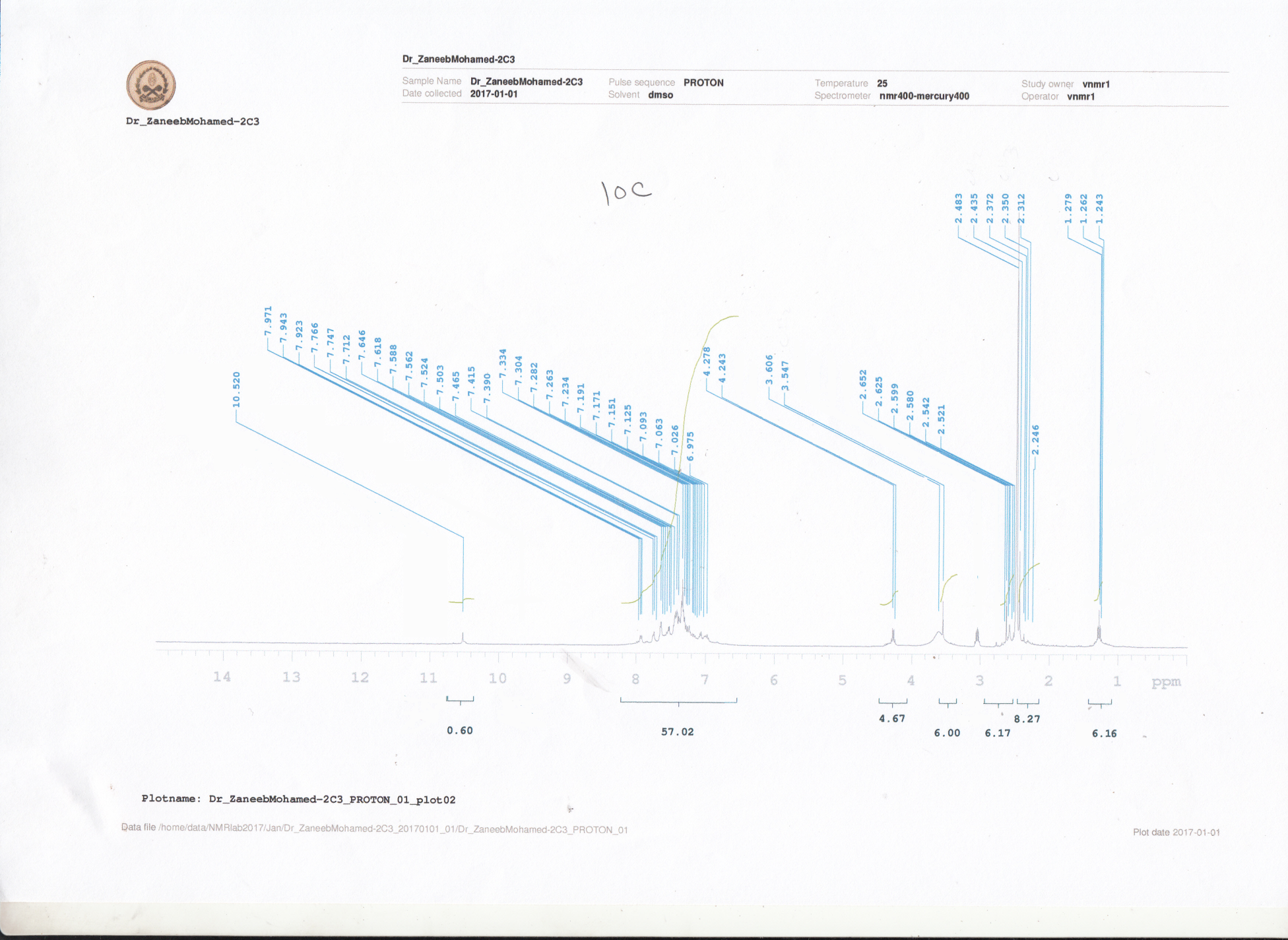


Compound **10c** (^1^HNMR)


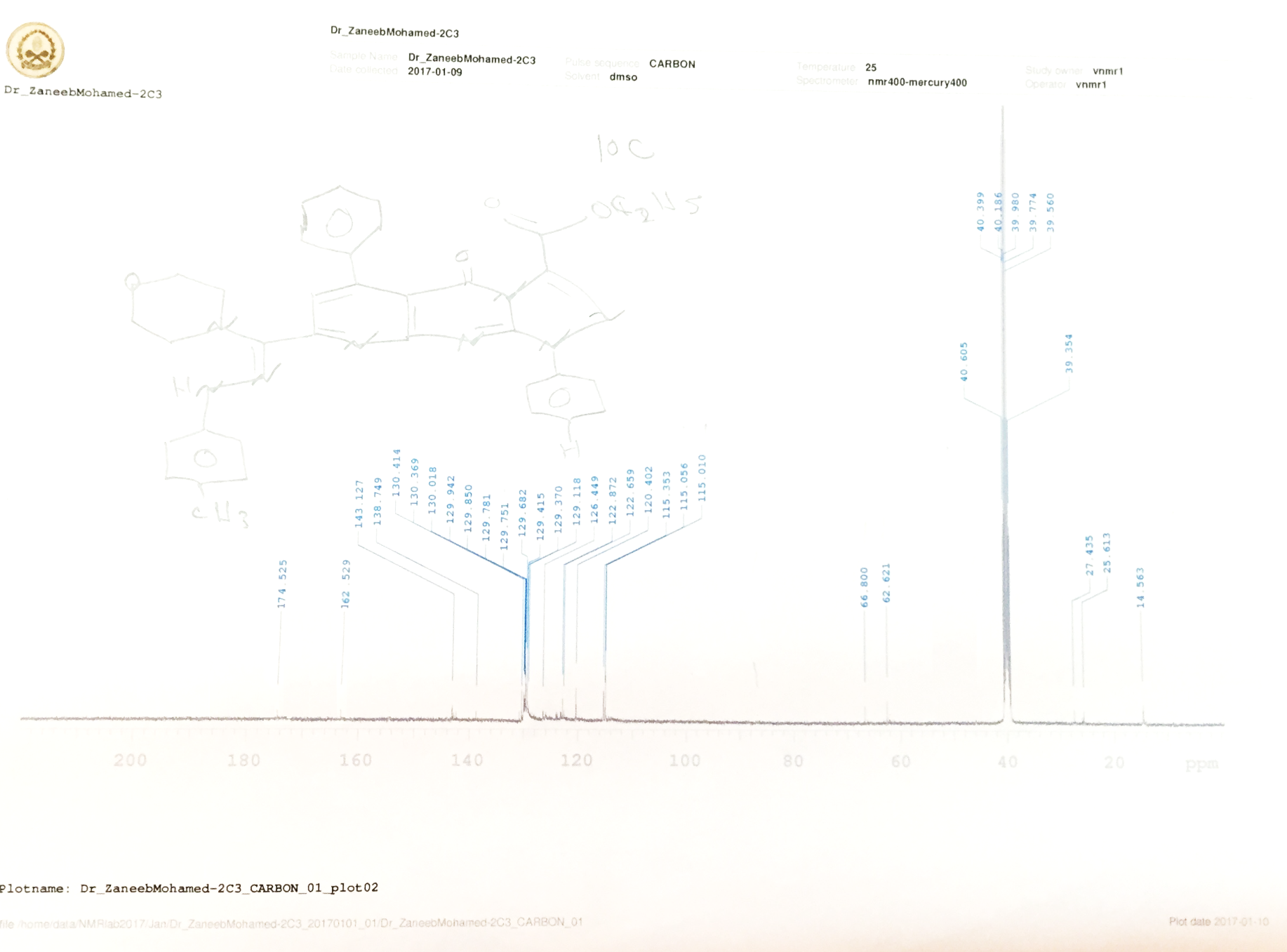


Compound **10c** (^13^C-NMR)


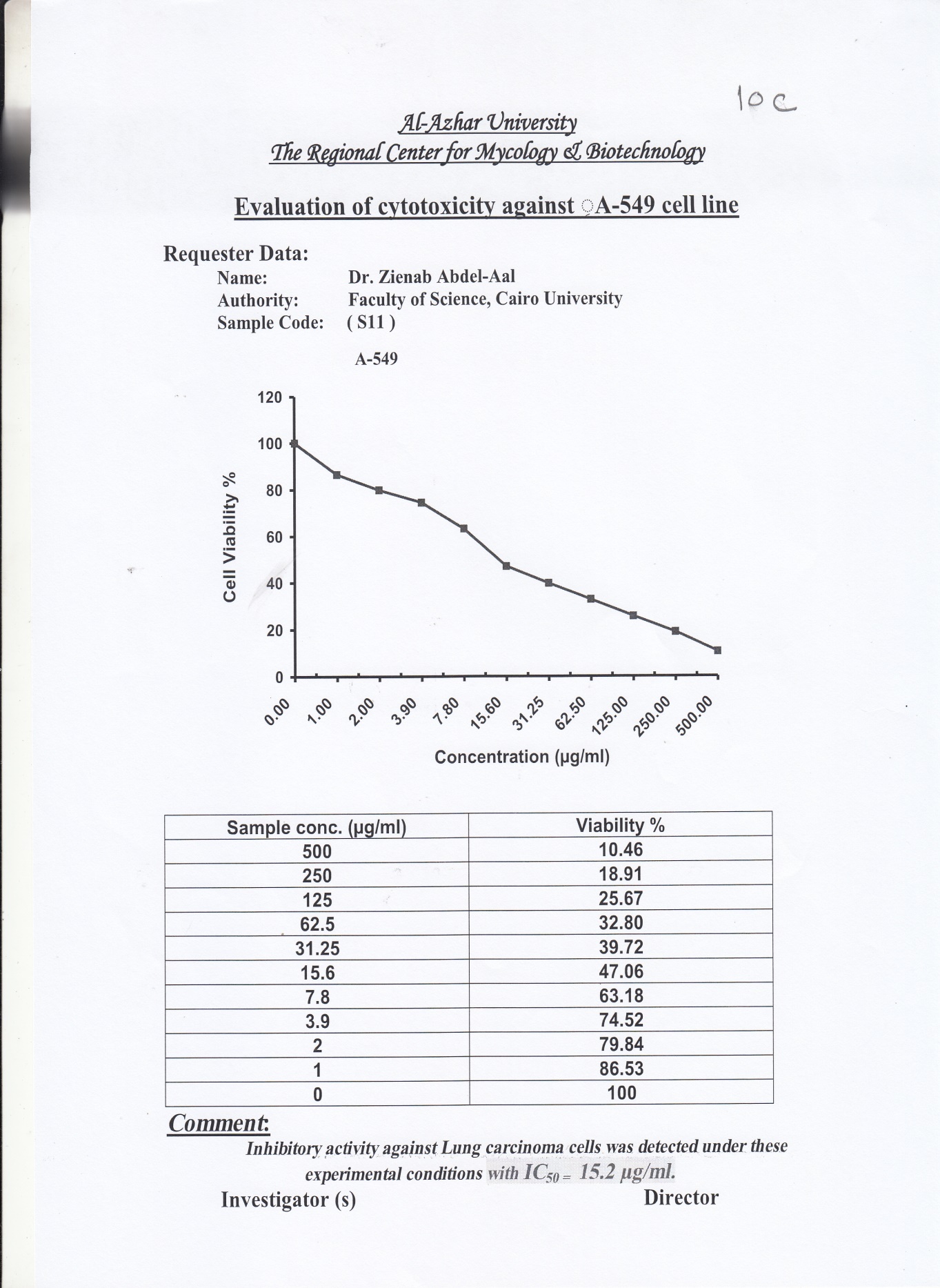


Compound **10c** (Cytotoxic activity against A-549)


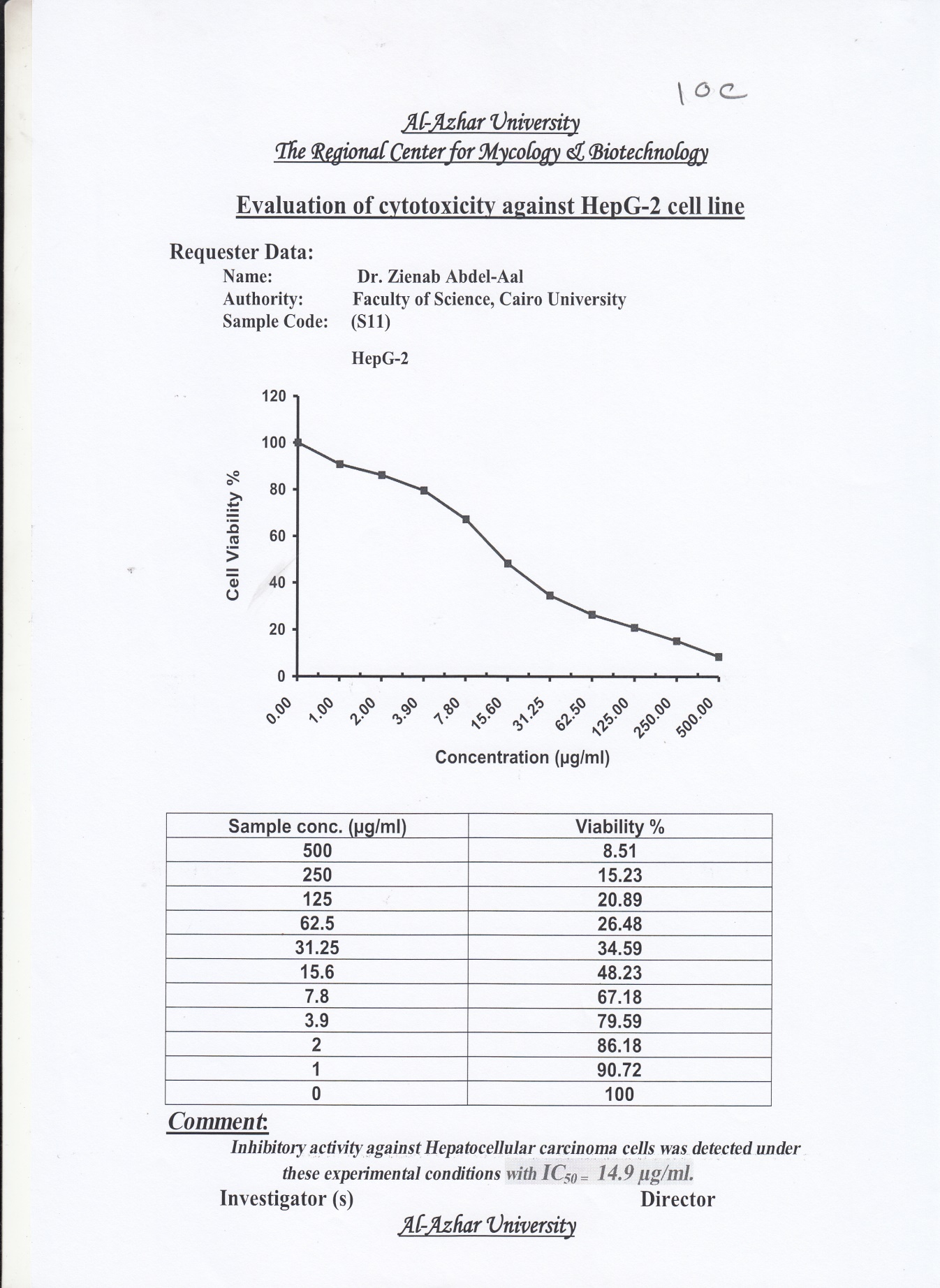


Compound **10c** (Cytotoxic activity against HepG-2)


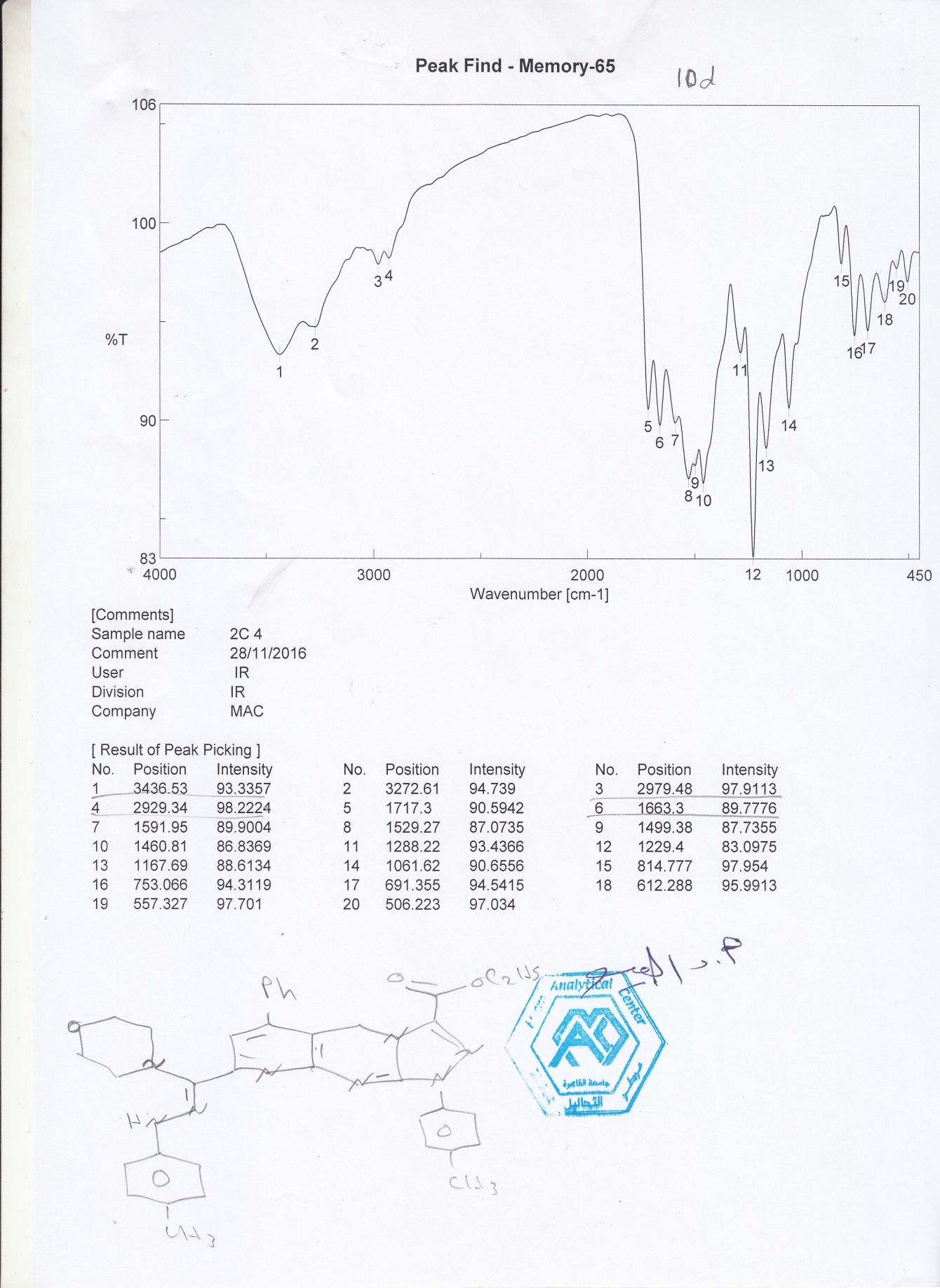


Compound **10d** (IR)


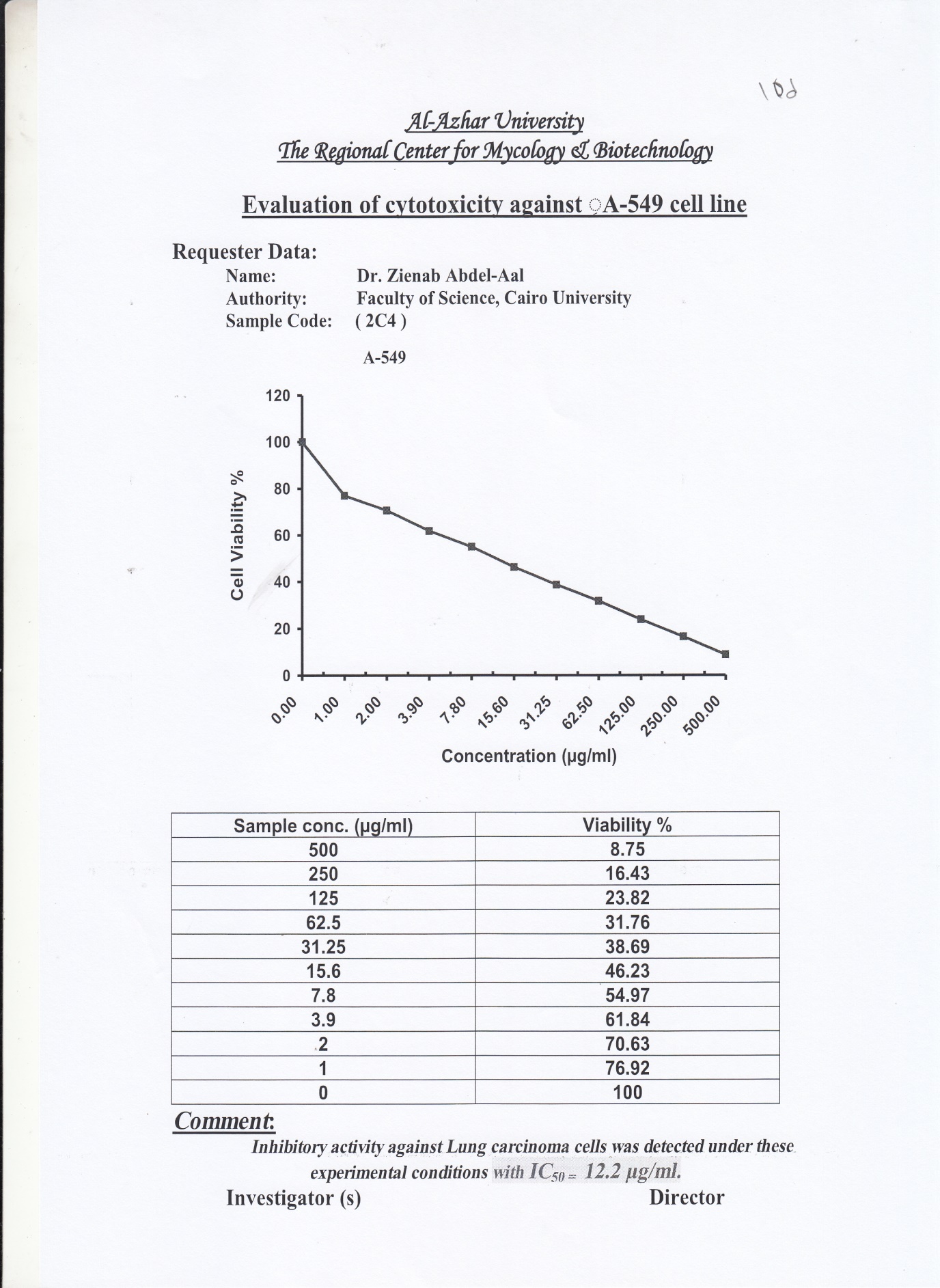


Compound **10d** (Cytotoxic activity against A-549)


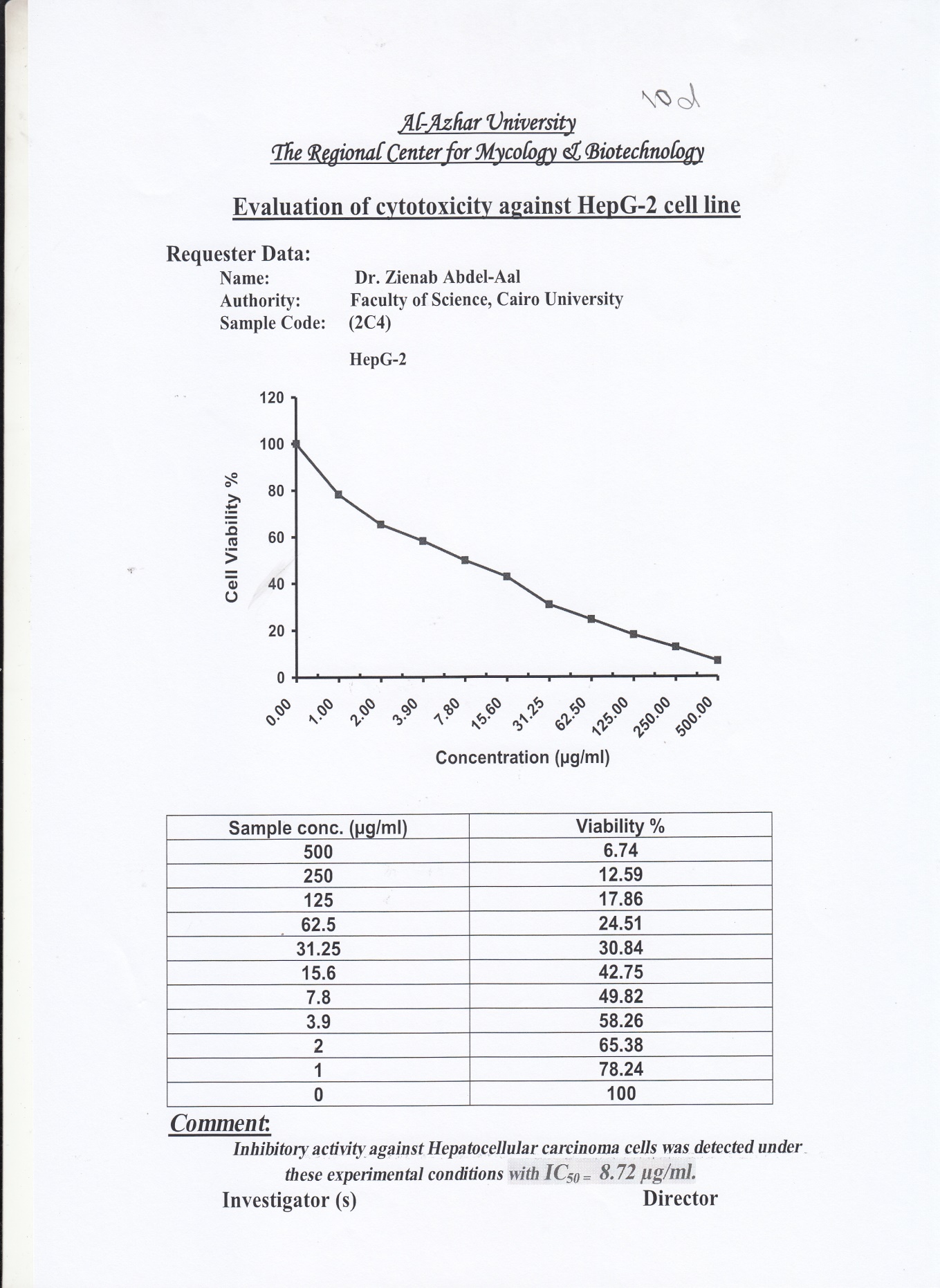


Compound **10d** (Cytotoxic activity against HepG-2)
